# ZBTB11 Promotes Breast Cancer Progression by Activating FBXO28‐Mediated MST1 Degradation and Suppressing Hippo Signaling

**DOI:** 10.1002/advs.76618

**Published:** 2026-07-20

**Authors:** An Xu, Xiang‐Nan Xu, Xiao Huang, Zhou Luo, Chun‐Lian Li, Yang Du, Cheng Yan, Xiao‐Jie Yu, Jian‐Wen Wang, Long‐Di Yao, De‐Yuan Fu

**Affiliations:** ^1^ Department of General Surgery Northern Jiangsu People's Hospital Affiliated to Yangzhou University Yangzhou Jiangsu China; ^2^ Department of Thyroid and Breast Surgery Xinghua People's Hospital Affiliated to Yangzhou University Xinghua Jiangsu China; ^3^ Department of Thyroid and Breast Surgery Changxing Hospital of Traditional Chinese Medicine Huzhou Zhejiang China

**Keywords:** breast cancer, epithelial‐mesenchymal transition, Hippo signaling, ubiquitin‐proteasome system

## Abstract

Epithelial‐mesenchymal transition (EMT) is a key driver of breast cancer progression, yet the upstream transcriptional and ubiquitin‐mediated mechanisms that modulate Hippo signaling remain incompletely defined. In this study, we identify a regulatory axis in which the transcription factor ZBTB11 promotes breast cancer aggressiveness by enhancing the expression of the F‐box protein FBXO28. ZBTB11 directly binds to the FBXO28 promoter and increases its transcription, leading to elevated FBXO28 protein levels. We show that FBXO28 functions as an E3 ubiquitin ligase that targets the core Hippo kinase MST1 for K48‐linked ubiquitination and proteasomal degradation. Reduction of MST1 diminishes Hippo pathway activity, resulting in decreased phosphorylation and enhanced nuclear accumulation of Yes‐associated protein (YAP) and transcriptional coactivator with PDZ‐binding motif (TAZ), which subsequently activates EMT‐related gene expression. Functionally, disruption of the ZBTB11‐FBXO28‐MST1 axis suppresses EMT, migration, invasion, and tumor growth in vitro and in vivo, whereas reintroduction of FBXO28 or depletion of MST1 reverses these effects. Together, our findings reveal a previously unrecognized transcription‐ubiquitination cascade that modulates Hippo signaling and contributes to breast cancer progression, highlighting this axis as a candidate therapeutic vulnerability that warrants further validation for limiting metastasis.

## Graphical abstract

ZBTB11 is identified as an oncogenic transcription factor that activates FBXO28 in breast cancer. FBXO28 promotes K48‐linked ubiquitination and degradation of MST1, suppressing Hippo signaling and enhancing epithelial–mesenchymal transition and metastasis. This transcription‐to‐ubiquitination cascade defines a prognostic biomarker axis and highlights a candidate therapeutic vulnerability in aggressive breast cancer.

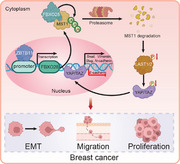

## 1 Introduction

Breast cancer remains the most commonly diagnosed malignancy and one of the leading causes of cancer‐related mortality among women worldwide [1, 2, 3]. Most breast cancer‐related deaths are attributable to distant metastasis, underscoring the importance of understanding the molecular mechanisms that drive metastatic progression [4, 5, 6]. Epithelial‐mesenchymal transition (EMT) is a key biological program through which carcinoma cells acquire enhanced motility, invasiveness, and metastatic competence [7, 8]. Although multiple signaling pathways, including transforming growth factor‐β (TGF‐β), Wnt, and Notch, contribute to EMT regulation, how upstream transcriptional programs are integrated with ubiquitin‐dependent post‐translational mechanisms to control EMT‐associated signaling remains incompletely understood [9, 10, 11, 12, 13].

The Hippo pathway is a central tumor‐suppressive network that regulates tissue homeostasis, cell proliferation, stemness, and cancer progression [14, 15]. Its core kinase cascade, composed of MST1/2 and LATS1/2, restrains the transcriptional coactivators Yes‐associated protein (YAP) and transcriptional coactivator with PDZ‐binding motif (TAZ) by promoting their phosphorylation, cytoplasmic retention, and degradation [15, 16, 17, 18, 19, 20]. When Hippo signaling is suppressed, YAP/TAZ accumulate in the nucleus, interact with TEA domain (TEAD) transcription factors, and activate genes involved in proliferation, survival, EMT, and metastasis [20, 21, 22]. Among these transcriptional outputs, CCN1 (CYR61) and CCN2 (CTGF) are widely used representative YAP/TAZ‐TEAD target genes and have been implicated in extracellular matrix remodeling, migration, invasion, and EMT‐associated malignant phenotypes [23, 24, 25]. Although the downstream regulation of YAP/TAZ has been extensively studied, the mechanisms controlling the stability of upstream Hippo kinases, particularly MST1, remain less well defined. Previous work has shown that MST1 can undergo ubiquitin‐dependent degradation, as exemplified by RNF6‐mediated MST1 ubiquitination in breast cancer [26]. However, whether F‐box proteins directly regulate MST1 stability and thereby suppress Hippo signaling remains largely unknown. In particular, no previous study has reported that FBXO28 targets MST1 for ubiquitination or connects FBXO28 to MST1‐dependent Hippo pathway suppression in breast cancer.

FBXO28, a member of the F‐box protein family that serves as the substrate‐recognition subunit of SCF (SKP1‐CUL1‐F‐box) ubiquitin ligase complexes, has recently emerged as a potential oncogenic regulator [27, 28]. Like other F‐box proteins, FBXO28 can promote substrate ubiquitination and proteasomal degradation. Recent studies have linked FBXO28 to pancreatic cancer proliferation, invasion, and metastasis through SMARCC2 ubiquitination, ovarian cancer progression through TGF‐β1/SMAD2/3 signaling, glioblastoma hypoxia‐inducible factor‐1α (HIF‐1α) signaling through PFKFB4, and breast cancer tumorigenesis through MYC proto‐oncogene, bHLH transcription factor (MYC)‐driven transcription [27, 28, 29, 30]. These findings support the broader relevance of FBXO28 in tumor biology. However, despite these observations, the upstream transcriptional cues that regulate FBXO28 expression and the downstream substrates through which FBXO28 controls major oncogenic signaling pathways remain incompletely defined. In particular, prior studies did not identify MST1 as an FBXO28 substrate, nor did they establish FBXO28 as a regulator of Hippo kinase signaling. Therefore, whether an oncogenic transcription factor can activate FBXO28 to induce MST1 degradation and thereby suppress Hippo signaling in breast cancer remains unknown—a gap that this study addresses by defining the ZBTB11–FBXO28–MST1 axis.

ZBTB11 is a broad‐complex, tramtrack, and bric‐à‐brac (BTB)/poxvirus and zinc finger (POZ)‐type zinc‐finger transcription factor involved in diverse biological processes, including neutrophil differentiation, mitochondrial function, pluripotency control, and hematopoietic regulation [31, 32, 33, 34]. Emerging evidence suggests that ZBTB11 may also contribute to tumor progression in specific cancer contexts [34, 35, 36, 37]. In lung cancer, ZBTB11 has been reported to cooperate with SET nuclear proto‐oncogene (SET) to promote metastasis through transcriptional activation of matrix metallopeptidase 9 (MMP9) [37]. Nevertheless, the direct transcriptional targets and downstream signaling pathways controlled by ZBTB11 in breast cancer remain largely unknown. Whether ZBTB11 drives EMT and metastatic progression in breast cancer, and whether it connects oncogenic transcriptional regulation with ubiquitin‐mediated Hippo pathway suppression, remains an unresolved question.

Based on these considerations, we hypothesized that ZBTB11 promotes breast cancer progression by transcriptionally activating downstream effectors that regulate Hippo signaling. In this study, we identify FBXO28 as a direct transcriptional target of ZBTB11 and demonstrate that FBXO28 promotes K48‐linked ubiquitination and proteasomal degradation of MST1, thereby suppressing Hippo signaling and enhancing YAP/TAZ‐associated EMT and malignant phenotypes. By integrating transcriptomic screening, promoter‐binding assays, ubiquitination analyses, functional rescue experiments, non‐triple‐negative breast cancer validation, and clinical correlation analyses, we define a previously unrecognized ZBTB11‐FBXO28‐MST1 transcription‐to‐ubiquitination cascade that links oncogenic transcriptional activation to Hippo pathway suppression and breast cancer progression.

## 2 Results

### 2.1 ZBTB11 is Upregulated in Metastatic Breast Cancer and Elevated in Primary Tumors With Prognostic Relevance

**FIGURE 1:**
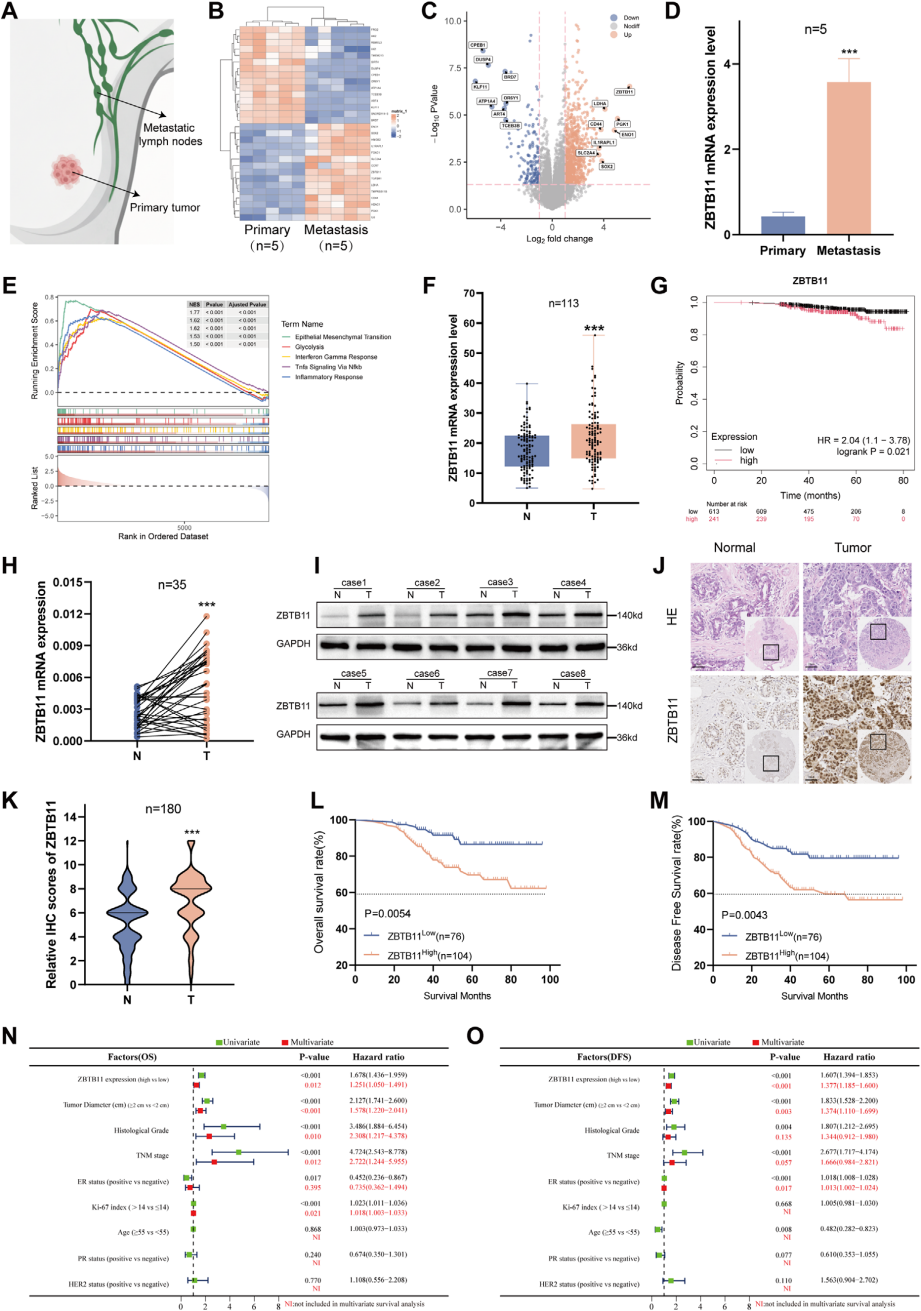
Expression profile and clinical relevance of ZBTB11 in metastatic and primary breast cancer. (A) Collection of paired primary breast tumors and matched metastatic lymph nodes for RNA sequencing. (B) Heatmap of the top 30 differentially expressed genes between primary tumors and metastatic lymph nodes. (C) Volcano plot of differentially expressed genes highlighting ZBTB11. (D) RNA‐seq expression of ZBTB11 in five paired primary and metastatic tissues. (E) Gene set enrichment analysis (GSEA) analysis of metastatic versus primary tumors. (F) ZBTB11 expression in 113 paired breast cancer and adjacent normal tissues from TCGA. (G) Kaplan–Meier overall survival curves from the KM‐plotter database. (H) RT‐qPCR analysis of ZBTB11 mRNA levels in 35 paired breast cancer and adjacent tissues. (I) Western blot analysis of ZBTB11 in eight paired breast cancer and adjacent tissues. (J) Representative immunohistochemistry (IHC) staining of ZBTB11 in a tissue microarray (TMA) of 180 paired breast cancer and adjacent tissues. (K) Quantification of ZBTB11 IHC scores in the TMA cohort. (L, M) Kaplan–Meier curves for overall survival (L) and disease‐free survival (M) according to ZBTB11 expression. (N, O) Univariate and multivariate Cox regression analyses for overall survival (N) and disease‐free survival (O). ^*^ *p* < 0.05, ^**^ *p* < 0.01, ^***^ *p* < 0.001, ns, not significant.

To identify metastasis‐associated molecular alterations in breast cancer, we collected paired primary breast tumors and matched metastatic lymph nodes from five patients for RNA sequencing (RNA‐seq) (Figure 1A). Differential expression analysis revealed a distinct transcriptional pattern between primary and metastatic lesions, and unsupervised clustering of the top 30 differentially expressed genes clearly separated the two tissue types (Figure 1B and Data S1). Volcano plot analysis further identified ZBTB11 as the most significantly upregulated gene in metastatic lesions (Figure 1C). Consistently, RNA‐seq quantification confirmed markedly elevated ZBTB11 expression in all five metastatic samples compared with their paired primary tumors (Figure 1D). Gene set enrichment analysis (GSEA) indicated that the HALLMARK_Epithelial_Mesenchymal_Transition pathway was the most significantly enriched in metastatic lesions (Figure 1E), suggesting a potential role of ZBTB11 in EMT‐related metastatic progression.

To determine whether ZBTB11 upregulation observed in metastatic lesions also occurs broadly in breast cancer, we examined ZBTB11 expression in large public datasets and independent clinical cohorts. Analysis of 113 paired breast cancer and adjacent tissues from The Cancer Genome Atlas (TCGA) demonstrated significantly higher ZBTB11 transcript levels in tumors (Figure 1F). Using the Kaplan‐Meier plotter (KM‐plotter) platform, patients with high ZBTB11 expression exhibited significantly worse overall survival (Figure 1G). Validation in our independent patient cohort further supported these findings. Reverse transcription quantitative PCR (RT‐qPCR) analysis of 35 paired breast cancer and adjacent tissues confirmed consistently increased ZBTB11 mRNA levels in tumors (Figure 1H). Western blotting of eight paired samples demonstrated concordant protein‐level overexpression (Figure 1I). Immunohistochemistry (IHC) performed on a tissue microarray (TMA) comprising 180 paired breast cancer and adjacent tissues revealed markedly stronger ZBTB11 protein expression in tumor tissues (Figure 1J), with quantitative analysis confirming significant elevation (Figure 1K).

Based on ZBTB11 expression in the tissue microarray (TMA) cohort, patients were stratified into high‐ and low‐expression groups according to the median immunoreactivity score (IRS) value. Comparison of baseline clinical characteristics between these groups is summarized in Table S1, showing that ZBTB11‐high expression was significantly associated with larger tumor diameter, higher tumor‐node‐metastasis (TNM) stage, and elevated Ki‐67 index. Kaplan–Meier survival analysis demonstrated that high ZBTB11 expression was associated with significantly poorer overall survival (OS) and disease‐free survival (DFS) (Figure 1L,M).

To further evaluate the prognostic value of ZBTB11, univariate and multivariate Cox proportional hazards regression analyses were performed. Variables examined in univariate analyses included ZBTB11 expression, tumor diameter, histological grade, TNM stage, age, estrogen receptor (ER) status, progesterone receptor (PR) status, human epidermal growth factor receptor 2 (HER2) status, and Ki‐67 index. The proportional hazards assumption was assessed using Schoenfeld residuals, and cases with missing clinicopathological or follow‐up information were excluded from the corresponding analyses. Detailed hazard ratios (HRs), 95% confidence intervals (CIs), and *p* values for OS and DFS are provided in Table S2.

Univariate Cox regression identified ZBTB11, tumor diameter, grade, stage, ER status, and Ki‐67 as significant prognostic factors for OS, and multivariate analysis confirmed that ZBTB11, tumor diameter, grade, stage, and Ki‐67 were independent predictors. Similarly, ZBTB11, tumor diameter, grade, stage, and Ki‐67 were significantly associated with DFS in univariate analysis, and ZBTB11, tumor diameter, and Ki‐67 remained independent predictors in multivariate analysis (Figure 1N,O). Collectively, these findings demonstrate that ZBTB11 is the most significantly upregulated gene in metastatic breast cancer, is broadly overexpressed in primary breast tumors, and functions as an independent prognostic biomarker for patient survival.

### 2.2 ZBTB11 Enhances the Proliferative, Invasive, and Metastatic Capacities of Breast Cancer Cells In Vitro and In Vivo

Since ZBTB11 was markedly elevated in metastatic and primary breast tumors and correlated with patient prognosis, we next investigated whether ZBTB11 plays a functional role in breast cancer progression. To identify suitable models, we first evaluated ZBTB11 expression across a panel of breast cancer cell lines. RT‐qPCR analysis revealed that, among the cell lines examined in our experimental system, ZBTB11 mRNA levels were relatively higher in several triple‐negative breast cancer (TNBC) cell lines, including MDA‐MB‐231, CAL51, HCC1937, and SUM159PT, whereas lower levels were observed in the ERBB2‐positive SK‐BR‐3 and MDA‐MB‐453 cells and the ER‐positive MCF7 and T47D cells (Figure S1A). Western blotting showed a generally consistent pattern at the protein level in our cell line panel (Figure S1B,C). This pattern differs from the RNA‐seq/normalized transcripts per million (nTPM) and mass spectrometry (MS)‐based normalized relative protein expression (nRPX) profiles reported in the Human Protein Atlas cell line resource, likely reflecting platform‐ and normalization‐related differences between RNA‐seq and qPCR, as well as between MS‐based proteomics and immunoblotting [38]. Therefore, model selection was based on qPCR and immunoblot validation performed in the same experimental system used for functional assays.

Based on these results, MDA‐MB‐231 and CAL51 cells, which showed relatively high endogenous ZBTB11 levels in our experimental system, were selected for both knockdown and overexpression studies. Stable knockdown lines were generated using three short hairpin RNAs (shRNAs), and RT‐qPCR and Western blotting confirmed that sh#2 and sh#3 efficiently reduced ZBTB11 expression in both cell lines (Figure S1D–F). To determine whether further elevation of ZBTB11 could enhance malignant phenotypes in high‐ZBTB11 backgrounds, FLAG‐tagged ZBTB11 was ectopically expressed in MDA‐MB‐231 and CAL51 cells and validated at the mRNA and protein levels (Figure S1G,H). In parallel, MCF7 and SK‐BR‐3 cells, which displayed relatively low endogenous ZBTB11 expression in our panel, were included as complementary low‐ZBTB11 gain‐of‐function models, and ZBTB11 overexpression was also confirmed in these cells (Figure S1G,I).

**FIGURE 2:**
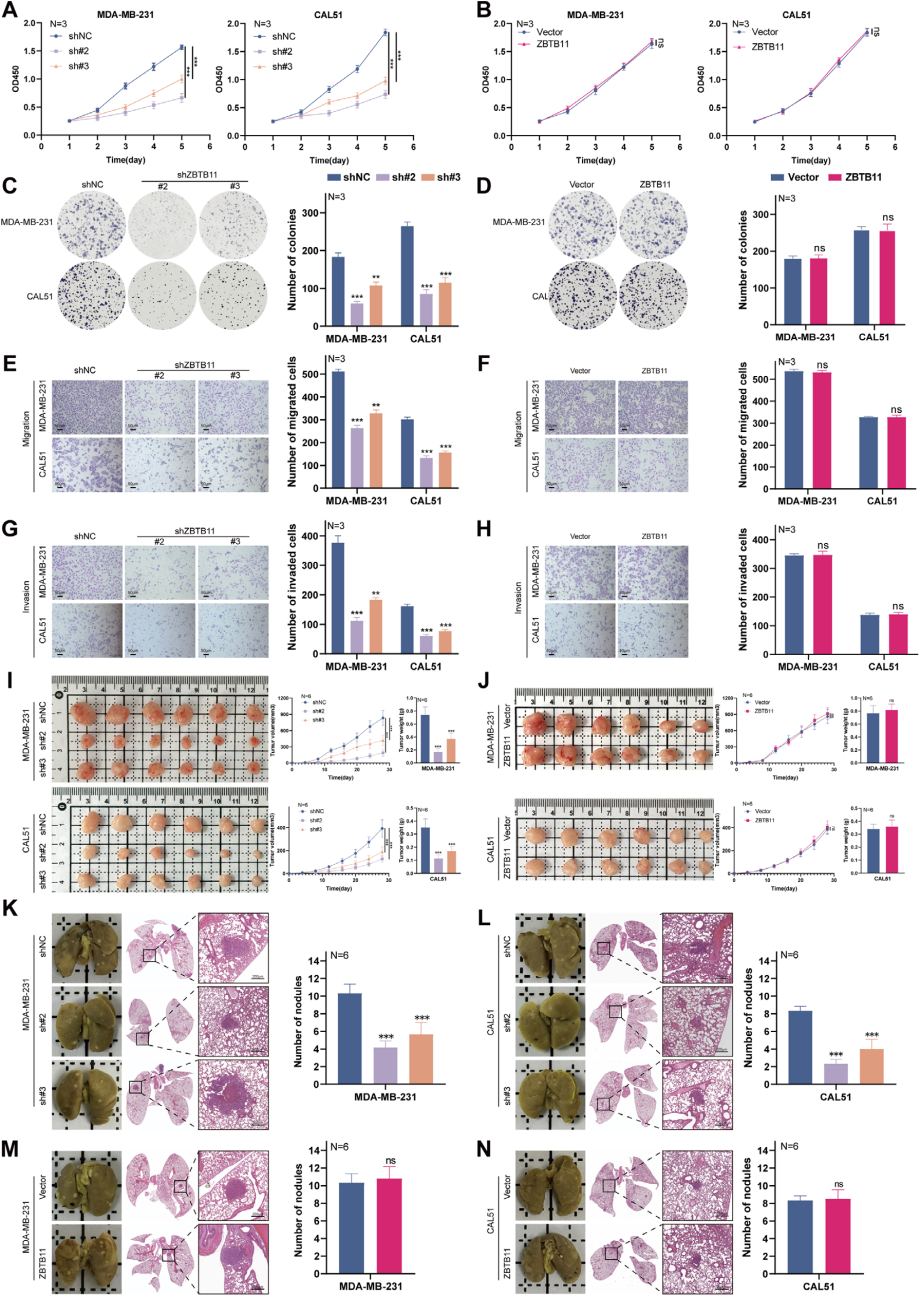
Effects of ZBTB11 knockdown and overexpression on breast cancer cell proliferation, migration, invasion, tumor growth, and metastasis. (A,B) CCK‐8 assays of ZBTB11 knockdown (A) and overexpression (B) cells. (C,D) Colony formation assays of knockdown (C) and overexpression (D) groups. (E,F) Transwell migration assays of knockdown (E) and overexpression (F) groups. (G,H) Transwell invasion assays of knockdown (G) and overexpression (H) groups. (I) Orthotopic mammary fat pad xenograft images images, tumor growth curves, and tumor weights from ZBTB11‐knockdown models. (J) Orthotopic mammary fat pad xenograft of ZBTB11‐overexpression models. (K,L) Lung metastasis assays following tail‐vein injection of knockdown MDA‐MB‐231 (K) and CAL51 (L) cells. (M, N) Lung metastasis assays of ZBTB11‐overexpression MDA‐MB‐231 (M) and CAL51 (N) cells. ^*^ *p* < 0.05, ^**^ *p* < 0.01, ^***^ *p* < 0.001, ns, not significant.

We next examined the functional impact of ZBTB11. Cell Counting Kit‐8 (CCK‐8) assays showed that ZBTB11 knockdown significantly reduced proliferation in MDA‐MB‐231 and CAL51 cells, with sh#2 exhibiting the strongest inhibitory effect (Figure 2A). In contrast, ZBTB11 overexpression did not significantly alter proliferation in either cell line (Figure 2B). Consistently, ZBTB11 knockdown markedly decreased colony formation, whereas overexpression did not increase clonogenic potential (Figure 2C,D). Transwell assays further showed that ZBTB11 depletion impaired migration and invasion, whereas ectopic ZBTB11 overexpression did not further enhance these phenotypes (Figure 2E–H). These results indicate that endogenous ZBTB11 is required to maintain aggressive phenotypes in MDA‐MB‐231 and CAL51 cells, while additional ZBTB11 expression does not confer further advantage in these high‐ZBTB11 TNBC models.

Because MDA‐MB‐231 and CAL51 cells already exhibited high basal ZBTB11 expression, the lack of additional phenotypic enhancement upon ZBTB11 overexpression may reflect a ceiling effect. To test this possibility, we performed gain‐of‐function assays in relatively low‐ZBTB11 MCF7 and SK‐BR‐3 cells. In contrast to the results observed in MDA‐MB‐231 and CAL51 cells, ZBTB11 overexpression significantly enhanced proliferation and colony formation in both MCF7 and SK‐BR‐3 cells (Figure S1J–M). In SK‐BR‐3 cells, ZBTB11 overexpression also promoted migration and invasion (Figure S1N,O). Because MCF7 cells displayed intrinsically weak migratory and invasive ability under our experimental conditions, Transwell assays were not included for this cell line. These findings support a threshold‐ or ceiling‐effect model in which ZBTB11 gain‐of‐function effects are more evident under a relatively low endogenous ZBTB11 background.

The in vitro observations were corroborated by in vivo studies. In orthotopic tumor formation assays, ZBTB11 knockdown significantly reduced tumor growth in both MDA‐MB‐231 and CAL51 models, as shown by smaller tumor volumes and lower tumor weights (Figure 2I). In contrast, ZBTB11 overexpression did not affect tumorigenicity in either model (Figure 2J). Tail‐vein injection assays showed that ZBTB11 knockdown markedly decreased lung metastatic colonization, as evidenced by fewer Bouin's‐stained nodules and metastatic lesions on hematoxylin and eosin (H&E) staining (Figure 2K,L), whereas ZBTB11 overexpression did not increase lung metastasis (Figure 2M,N). Together, these data demonstrate that ZBTB11 is required to sustain malignant phenotypes in high‐ZBTB11 TNBC models, whereas its gain‐of‐function effects become evident in relatively low‐ZBTB11 MCF7 and SK‐BR‐3 cells, supporting a context‐dependent, expression‐threshold model for ZBTB11‐mediated phenotypic regulation.

### 2.3 ZBTB11 Regulates Epithelial‐Mesenchymal Transition in Breast Cancer

**FIGURE 3:**
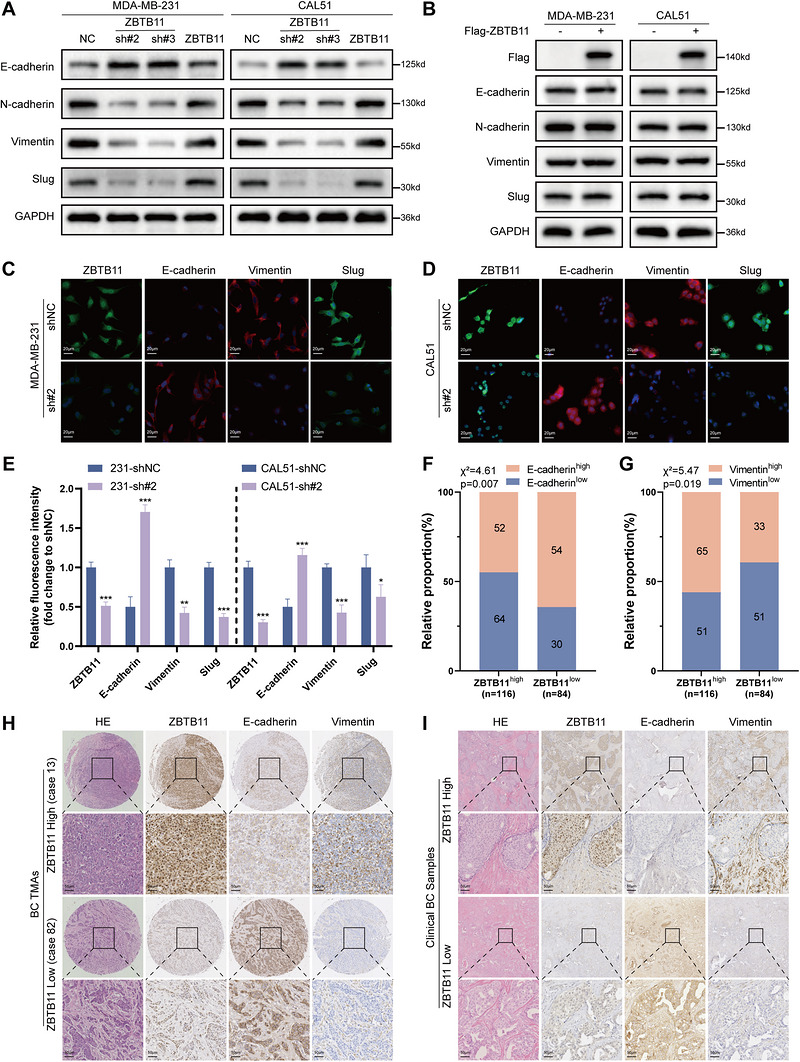
Effects of ZBTB11 on EMT markers in breast cancer cells and tissues. (A,B) Western blot analysis of EMT‐related proteins in ZBTB11‐knockdown (A) and ZBTB11‐overexpression (B) MDA‐MB‐231 and CAL51 cells. (C, D) Immunofluorescence staining of ZBTB11, E‐cadherin, Vimentin, and Slug in ZBTB11‐knockdown MDA‐MB‐231 (C) and CAL51 (D) cells. (E) Quantification of relative fluorescence intensity. (F,G) Correlation between ZBTB11 and E‐cadherin (F) or Vimentin (G) expression in breast cancer tissues (*n* = 200). (H) Representative IHC images of ZBTB11, E‐cadherin, and Vimentin in breast cancer TMAs. (I) Representative IHC images from an independent cohort of 20 clinical breast cancer samples. ^*^ *p* < 0.05, ^**^ *p* < 0.01, ^***^ *p* < 0.001, ns, not significant.

Given that EMT was the most significantly enriched pathway in metastatic breast cancer based on RNA‐seq analysis, we examined whether ZBTB11 functionally modulates EMT in breast cancer cells. Western blot analysis revealed that knockdown of ZBTB11 in MDA‐MB‐231 and CAL51 cells increased the epithelial marker E‐cadherin while reducing the mesenchymal markers N‐cadherin, Vimentin, and Slug, and re‐expression of ZBTB11 in sh#2 cells restored these protein levels (Figure 3A). In contrast, ectopic overexpression of ZBTB11 did not substantially alter EMT marker expression in MDA‐MB‐231 or CAL51 cells, both of which have high basal ZBTB11 expression (Figure 3B). However, in the relatively low‐ZBTB11 MCF7 and SK‐BR‐3 cells, ZBTB11 overexpression decreased the epithelial marker E‐cadherin and increased the mesenchymal markers N‐cadherin, Vimentin, and Slug (Figure S1P). These data indicate that ZBTB11 is sufficient to induce an EMT‐like shift under a low endogenous ZBTB11 expression background, whereas further ZBTB11 overexpression has a limited effect in high‐ZBTB11 TNBC cells. Immunofluorescence staining further confirmed that ZBTB11 depletion enhanced epithelial features and reduced mesenchymal marker signals in both cell lines (Figure 3C,D), with quantification showing consistent changes (Figure 3E). Clinically, ZBTB11‐high tumors showed weaker E‐cadherin and stronger Vimentin staining in the 180‐case TMA cohort and in an independent 20‐case validation cohort (Figure 3H,I). Combined analysis of 200 cases confirmed that high ZBTB11 expression was significantly associated with low E‐cadherin (Figure 3F; χ^2^ = 4.61, *p* = 0.007) and high Vimentin expression (Figure 3G; χ^2^ = 5.47, *p* = 0.019). Collectively, these findings indicate that ZBTB11 promotes an EMT‐like phenotype in an endogenous expression level‐dependent manner and is clinically associated with EMT marker expression.

### 2.4 ZBTB11 Transcriptionally Activates FBXO28 by Binding to its Promoter

**FIGURE 4:**
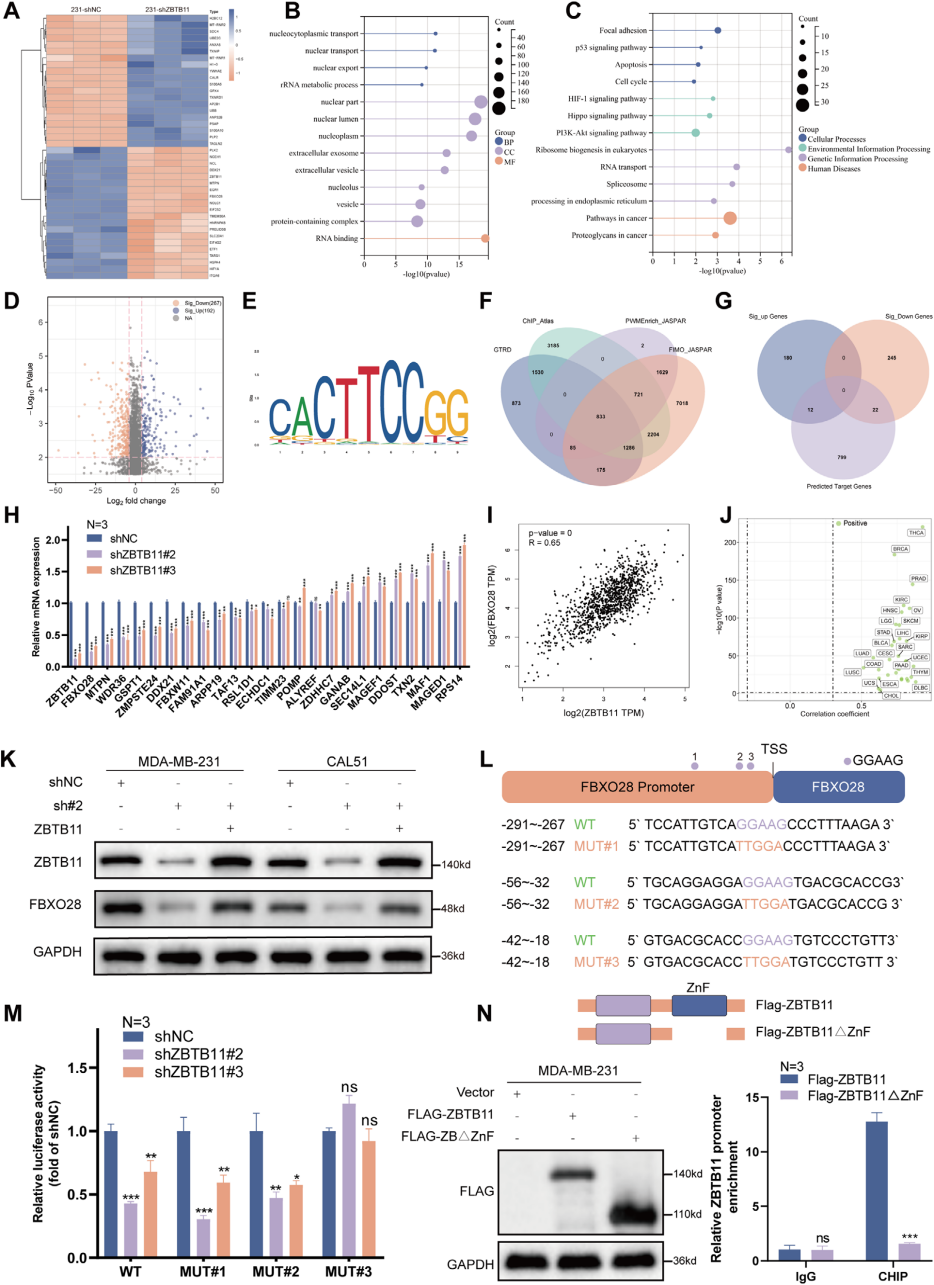
Transcriptomic analysis and identification of FBXO28 as a downstream target of ZBTB11. (A) Heatmap of the top 40 differentially expressed genes after ZBTB11 knockdown in MDA‐MB‐231 cells. (B) Gene Ontology (GO) enrichment analysis of differentially expressed genes. (C) Kyoto Encyclopedia of Genes and Genomes (KEGG) pathway enrichment analysis. (D) Volcano plot showing upregulated and downregulated genes. (E) JASPAR motif and sequence logo of predicted ZBTB11 binding sites. (F) Venn diagram of predicted ZBTB11 target genes from four databases. (G) Intersection of predicted targets with RNA‐seq–derived differentially expressed genes. (H) RT‐qPCR validation of selected candidate genes in MDA‐MB‐231 cells. (I) Correlation between ZBTB11 and FBXO28 expression in TCGA breast cancer samples. (J) Pan‐cancer correlation analysis of ZBTB11 and FBXO28. (K) Western blot analysis of FBXO28 expression in ZBTB11‐knockdown and rescue cells. (L) Schematic of wild‐type and mutant FBXO28 promoter constructs. (M) Dual‐luciferase assays assessing promoter activity of wild‐type and mutant FBXO28 constructs. (N) Schematic of ZBTB11 and ZBTB11‐ΔZnF constructs (top), Western blot validation (bottom left), and ChIP analysis of FBXO28 promoter binding (bottom right). ^*^ *p* < 0.05, ^**^ *p* < 0.01, ^***^ *p* < 0.001, ns, not significant.

To identify downstream effectors of ZBTB11, we performed RNA‐seq in MDA‐MB‐231 cells after ZBTB11 knockdown. A total of 459 genes were differentially expressed, including 267 downregulated and 192 upregulated genes, with enrichment in nuclear transport, RNA metabolism, apoptosis, cell cycle, hypoxia‐inducible factor‐1 (HIF‐1), Hippo signaling, and cancer‐related pathways (Figure 4A–D and Data S2). Integration of ZBTB11‐dependent differentially expressed genes (DEGs) with predicted ZBTB11 targets from FIMO_JASPAR, PWMEnrich_JASPAR, GTRD, and ChIP‐Atlas identified 34 overlapping candidates, including 12 upregulated and 22 downregulated genes (Figure 4E–G and Data S3). RT‐qPCR validation in MDA‐MB‐231 cells identified 24 reliably expressed candidates, among which FBXO28 exhibited one of the most consistent and pronounced reductions following ZBTB11 knockdown (Figure 4H). Given that FBXO28 encodes an F‐box E3 ligase, functionally compatible with regulating protein stability, we prioritized FBXO28 as the most biologically plausible downstream effector of ZBTB11 for further mechanistic investigation. Correlation analysis of TCGA breast cancer samples showed a positive association between ZBTB11 and FBXO28 transcript levels (Figure 4I), which was further supported by pan‐cancer analysis demonstrating a positive correlation across multiple cancer types (Figure 4J). Consistently, Western blot analysis in MDA‐MB‐231 and CAL51 cells showed that FBXO28 protein expression decreased upon ZBTB11 knockdown and was restored upon ZBTB11 re‐expression (Figure 4K). In the additionally included MCF7 and SK‐BR‐3 cells, ZBTB11 overexpression also increased FBXO28 protein levels, further supporting the positive regulation of FBXO28 by ZBTB11 in non‐TNBC breast cancer cell backgrounds (Figure S3G). To assess whether ZBTB11 directly regulates FBXO28 transcription, we cloned the wild‐type FBXO28 promoter and three promoter mutant (MUT) constructs harboring substitutions in the predicted “GGAAG” motif into pGL3 luciferase vectors (Figure 4L). Dual‐luciferase assays revealed that ZBTB11 knockdown reduced promoter activity in the wild‐type, MUT#1, and MUT#2 constructs; however, MUT#3 abolished this effect (Figure 4M), indicating that this motif is critical for ZBTB11‐mediated transcriptional activation. Because ZBTB11 contains a C2HC‐type zinc finger deoxyribonucleic acid (DNA)‐binding region (amino acids 569–937), we generated FLAG‐tagged full‐length ZBTB11 and a zinc finger‐deleted mutant (FLAG‐ZBTB11‐ΔZnF). Western blot analysis confirmed the expected molecular weights of the two constructs (Figure 4N, **left**). Chromatin immunoprecipitation (ChIP) assays demonstrated that full‐length ZBTB11, but not ZBTB11‐ΔZnF, was enriched at the FBXO28 promoter region (Figure 4N, **right**). Immunoglobulin G (IgG) controls showed minimal background binding. Collectively, these findings indicate that ZBTB11 directly binds to the FBXO28 promoter through its zinc finger domain and transcriptionally activates FBXO28, thereby identifying FBXO28 as a key downstream effector of ZBTB11.

### 2.5 FBXO28 Promotes Proliferation, Migration, Invasion, and Tumor Growth in Breast Cancer

To further investigate the downstream functional role of FBXO28, we generated stable FBXO28 knockdown and overexpression models in breast cancer cells (Figure S2). Three shRNAs were used to silence FBXO28, and RT‐qPCR analysis showed that all three constructs reduced FBXO28 expression, with shFBXO28#3 achieving the highest knockdown efficiency in both MDA‐MB‐231 and CAL51 cells (Figure S2A). Ectopic overexpression of FBXO28 markedly increased FBXO28 mRNA in both cell lines (Figure S2B). Western blotting confirmed corresponding changes at the protein level, with shFBXO28#3 yielding the most efficient suppression (Figure S2C,D). Based on these results, shFBXO28#3 was selected for loss‐of‐function studies in MDA‐MB‐231 cells, whereas CAL51 cells were used for gain‐of‐function experiments. FBXO28 silencing significantly impaired the proliferative capacity of MDA‐MB‐231 cells, as shown by reduced colony formation and lower CCK‐8 absorbance, whereas FBXO28 overexpression increased proliferation in CAL51 cells (Figure S2E–G). Similar trends were observed in cell motility assays: Transwell migration and invasion were reduced upon FBXO28 knockdown and increased upon FBXO28 overexpression (Figure S2H–K). In vivo, orthotopic mammary fat pad xenograft assays showed that FBXO28 knockdown markedly suppressed tumor growth in MDA‐MB‐231 xenografts, whereas FBXO28 overexpression enhanced tumor growth in CAL51 xenografts (Figure S2L–N). We next examined the effects of FBXO28 on EMT marker expression. Western blot analysis revealed that FBXO28 knockdown increased E‐cadherin levels and reduced N‐cadherin, Vimentin, and Slug expression in both MDA‐MB‐231 and CAL51 cells (Figure S2O). Conversely, FBXO28 overexpression decreased E‐cadherin and increased mesenchymal marker expression (Figure S2P), supporting a role for FBXO28 in promoting EMT‐like phenotypes. To determine whether the tumor‐promoting effect of FBXO28 extends beyond TNBC models, we further performed FBXO28 gain‐of‐function assays in MCF7 and SK‐BR‐3 cells. Successful FBXO28 overexpression was confirmed at both the protein and mRNA levels (Figure S3A,B). FBXO28 overexpression significantly enhanced cell proliferation in both MCF7 and SK‐BR‐3 cells, as shown by CCK‐8 assays (Figure S3C,D). Consistently, FBXO28 overexpression increased colony formation in both cell lines (Figure S3E,F). In SK‐BR‐3 cells, FBXO28 overexpression also promoted migratory and invasive capacities in Transwell assays (Figure S3H,I). Together, these results support a tumor‐promoting role for FBXO28 in TNBC cells and in the examined non‐TNBC breast cancer cell backgrounds.

### 2.6 FBXO28 Mediates ZBTB11‐Dependent EMT and Malignant Phenotypes in Breast Cancer Cells

**FIGURE 5:**
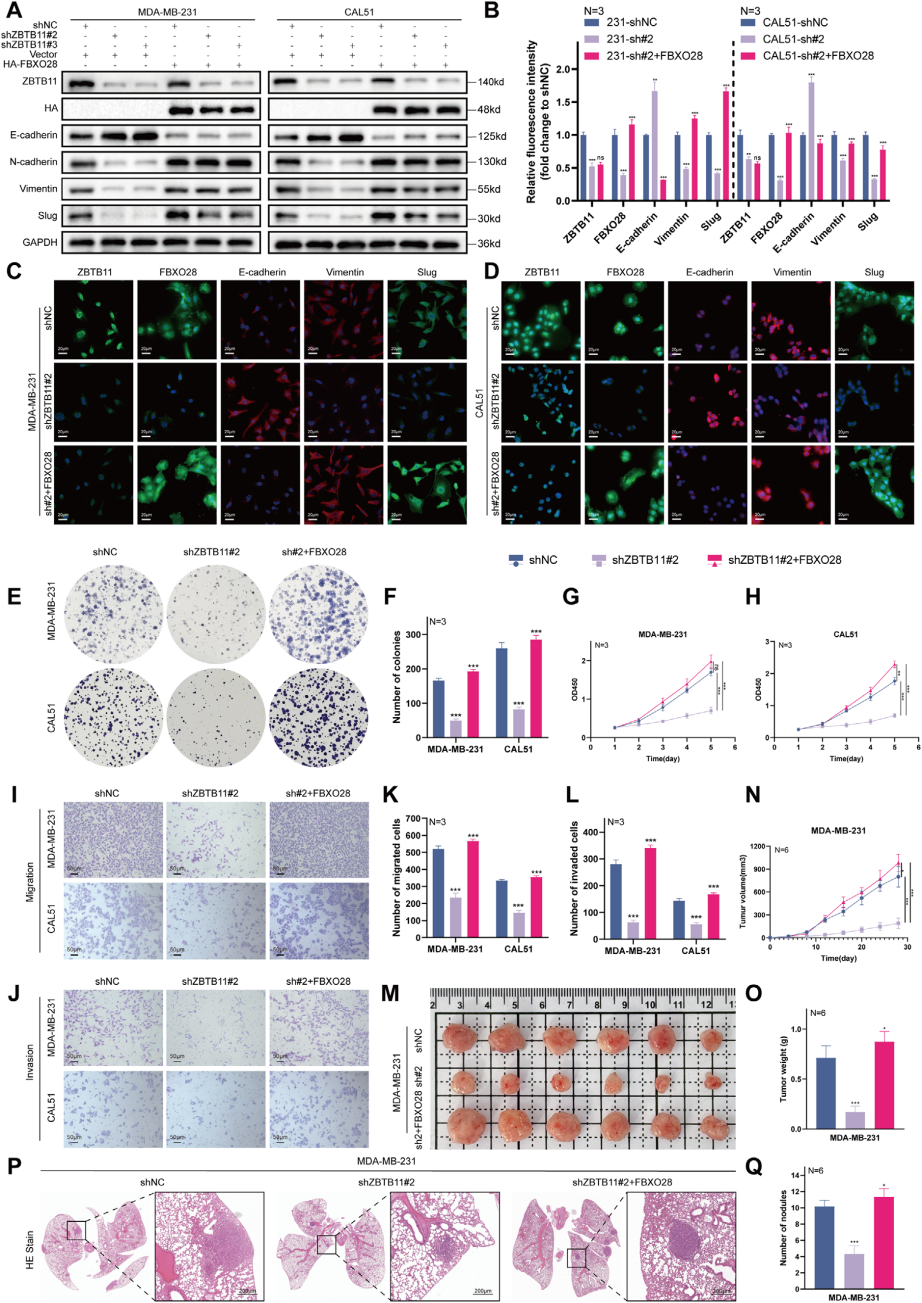
Effects of FBXO28 re‐expression on ZBTB11‐knockdown breast cancer cells. (A) Western blot analysis of ZBTB11, FBXO28, and EMT‐related proteins in MDA‐MB‐231 and CAL51 cells expressing shNC, shZBTB11, or shZBTB11 plus FBXO28. (B) Quantification of immunofluorescence intensity. (C,D) Immunofluorescence staining of ZBTB11, FBXO28, E‐cadherin, Vimentin, and Slug in MDA‐MB‐231 (C) and CAL51 (D) cells. (E) Colony formation assays in cells expressing shNC, shZBTB11, or shZBTB11 plus FBXO28. (F) Quantification of colony formation. (G,H) CCK‐8 proliferation assays of MDA‐MB‐231 (G) and CAL51 (H) cells. (I) Transwell migration assays in rescue models. (J) Transwell invasion assays in rescue models. (K) Quantification of migration assays. (L) Quantification of invasion assays. (M) Orthotopic mammary fat pad xenograft images from rescue experiments. (N) Tumor growth curves. (O) Tumor weight measurements. (P) Representative lung metastasis hematoxylin and eosin (H&E) images from tail‐vein injection models. (Q) Quantification of lung metastatic nodules. ^*^ *p* < 0.05, ^**^ *p* < 0.01, ^***^ *p* < 0.001, ns, not significant.

To determine whether FBXO28 mediates the functional effects of ZBTB11, we performed rescue experiments in MDA‐MB‐231 and CAL51 cells following ZBTB11 knockdown (Figure 5). Western blotting showed that ZBTB11 depletion increased E‐cadherin and reduced N‐cadherin, Vimentin, and Slug, whereas HA‐FBXO28 re‐expression substantially reversed these EMT marker changes in both MDA‐MB‐231 and CAL51 cells (Figure 5A). Immunofluorescence staining and quantification further confirmed that FBXO28 re‐expression restored the ZBTB11 knockdown‐induced changes in E‐cadherin, Vimentin, and Slug signals (Figures 5B–D). We next assessed whether FBXO28 restored the malignant phenotypes suppressed by ZBTB11 knockdown. In colony formation assays, ZBTB11 knockdown significantly reduced clonogenicity in MDA‐MB‐231 cells, whereas FBXO28 overexpression restored and even enhanced colony numbers relative to controls (Figure 5E,F). CCK‐8 proliferation assays yielded similar results in both cell lines (Figure 5G,H). Transwell assays showed that ZBTB11 depletion reduced migration and invasion, while FBXO28 overexpression rescued these phenotypes (Figure 5I–L). In vivo, orthotopic mammary fat pad xenograft assays demonstrated smaller tumors in the ZBTB11‐knockdown group and markedly larger tumors in the ZBTB11‐knockdown + FBXO28 group (Figure 5M–O). Tail‐vein injection assays further showed reduced lung metastatic burden upon ZBTB11 knockdown, which was restored by FBXO28 overexpression (Figure 5P,Q). To further validate the requirement of FBXO28 for ZBTB11‐driven phenotypes in low endogenous ZBTB11 and non‐TNBC cell backgrounds, we performed complementary rescue experiments in MCF7 and SK‐BR‐3 cells by combining ZBTB11 overexpression with FBXO28 knockdown. In both cell lines, ZBTB11 overexpression increased FBXO28 expression and promoted an EMT‐like shift, as shown by decreased E‐cadherin and increased N‐cadherin, Vimentin, and Slug. However, FBXO28 knockdown markedly attenuated these ZBTB11‐induced EMT changes (Figure S3G). Functionally, ZBTB11 overexpression enhanced cell proliferation in both MCF7 and SK‐BR‐3 cells, whereas FBXO28 knockdown reversed this effect, as shown by CCK‐8 assays (Figure S3J,K). Similarly, FBXO28 knockdown reduced the increased colony formation induced by ZBTB11 overexpression in both cell lines (Figure S3L,M). In SK‐BR‐3 cells, FBXO28 knockdown also attenuated the ZBTB11‐overexpression‐induced increases in migration and invasion (Figure S3N,O). These findings indicate that FBXO28 is required for ZBTB11‐induced EMT and malignant phenotypes in breast cancer cells with relatively low endogenous ZBTB11 expression. Collectively, these complementary rescue experiments demonstrate that FBXO28 is a key functional downstream effector of ZBTB11, restoring ZBTB11 knockdown‐suppressed malignant phenotypes in TNBC cells and mediating ZBTB11 overexpression‐driven EMT and tumor‐promoting effects in MCF7 and SK‐BR‐3 cells.

### 2.7 FBXO28 Directly Interacts With MST1 and Regulates its Protein Stability

**FIGURE 6:**
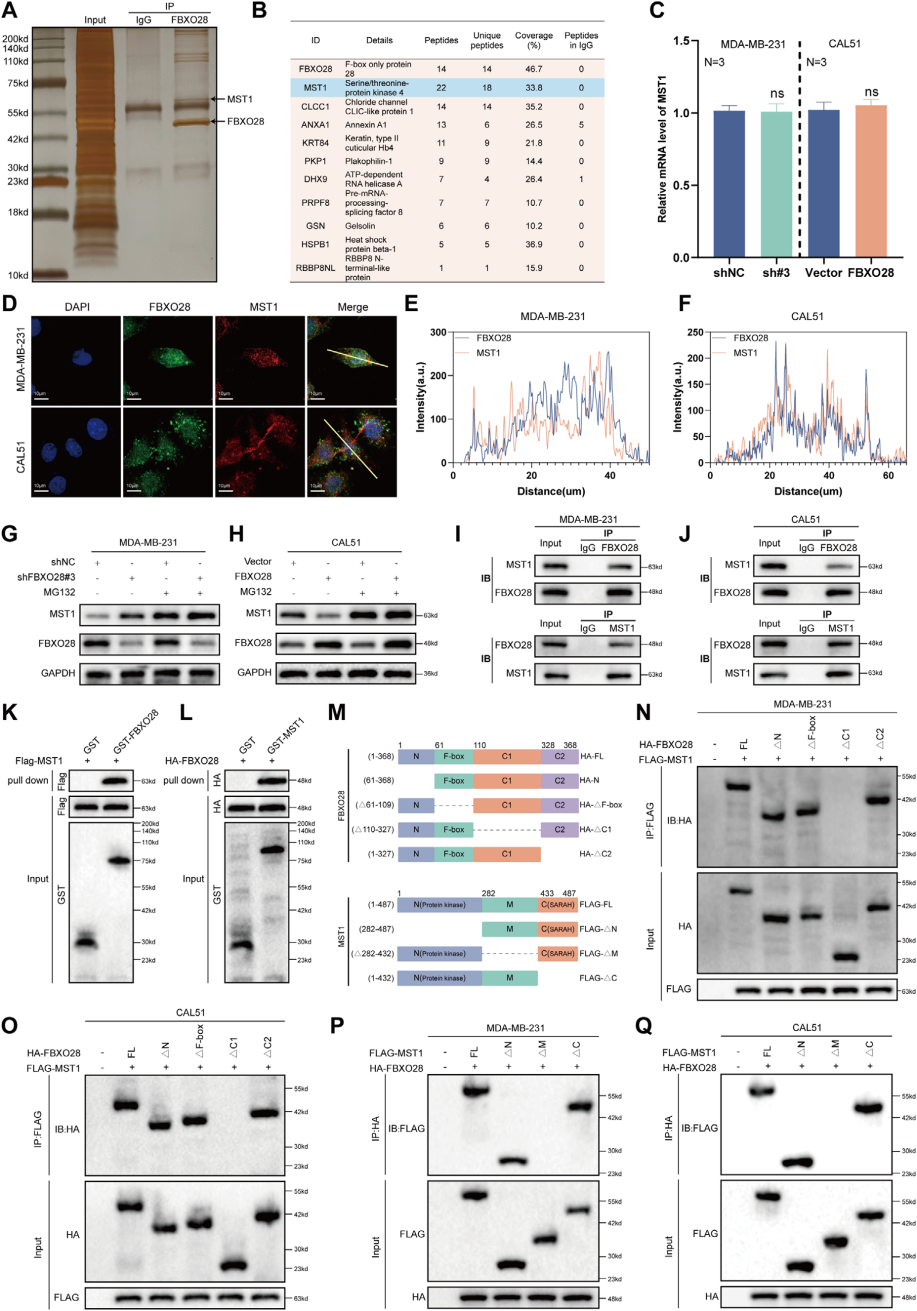
Identification and characterization of MST1 as an FBXO28‐interacting protein. (A) Silver staining of immunoprecipitated proteins from HA‐FBXO28 complexes. (B) Representative candidate FBXO28‐interacting proteins identified by HA‐FBXO28 immunoprecipitation and LC‐MS/MS. (C) RT‐qPCR analysis of MST1 mRNA levels following FBXO28 knockdown or overexpression. (D) Immunofluorescence images showing FBXO28 and MST1 localization in MDA‐MB‐231 and CAL51 cells. Yellow lines in the merged images indicate the regions used for fluorescence intensity profiling. (E, F) Line‐scan fluorescence intensity profiles of FBXO28 and MST1 across the indicated regions in MDA‐MB‐231 (E) and CAL51 (F) cells. (G) Western blot analysis of MST1 protein after FBXO28 knockdown with or without MG132 treatment. (H) Western blot analysis of MST1 protein after FBXO28 overexpression with or without MG132 treatment. (I,J) Co‐immunoprecipitation analysis of FBXO28 and MST1 in MDA‐MB‐231 (I) and CAL51 (J) cells. (K,L) GST pull‐down assays showing direct interaction between FBXO28 and MST1. (M) Schematic of FBXO28 and MST1 deletion mutants. (N,O) Co‐IP analysis of interactions between MST1‐FLAG and FBXO28 deletion mutants. (P,Q) Co‐IP analysis of interactions between FBXO28‐HA and MST1 deletion mutants. ^*^ *p* < 0.05, ^**^ *p* < 0.01, ^***^ *p* < 0.001, ns, not significant.

To identify proteins that interact with FBXO28, we performed immunoprecipitation followed by silver staining in MDA‐MB‐231 cells. The HA‐FBXO28 immunoprecipitated fraction displayed distinct bands compared with the IgG control (Figure 6A). Liquid chromatography‐tandem mass spectrometry (LC‐MS/MS) analysis of the differential bands identified MST1 as a candidate FBXO28‐interacting protein (Figure 6B), a kinase central to Hippo signaling. MST1 was detected with 22 peptides, including 18 unique peptides, corresponding to 35.8% sequence coverage. No MST1 peptides were detected in the IgG control. The label‐free quantification (LFQ) intensity of MST1 was 4,834,864 in HA‐FBXO28 immunoprecipitates, whereas MST1 was not detected in the IgG control. Full identification of all 657 candidate FBXO28‐interacting proteins is provided in Data S4. RT‐qPCR analysis indicated that FBXO28 knockdown or overexpression did not alter MST1 mRNA levels (Figure 6C), suggesting post‐transcriptional regulation. Immunofluorescence staining revealed that although FBXO28 predominantly localized to the nucleus, FBXO28 and MST1 displayed partial cytoplasmic overlap in both MDA‐MB‐231 and CAL51 cells, as illustrated by line‐scan fluorescence intensity profiling across the indicated regions (Figure 6D–F). Consistently, MG132 treatment abolished FBXO28‐dependent changes in MST1 protein abundance, supporting a proteasome‐dependent mechanism (Figure 6G,H). Multiple complementary assays further supported the interaction between FBXO28 and MST1. Bimolecular fluorescence complementation (BiFC) assays showed FBXO28‐MST1 interaction signals in both MDA‐MB‐231 and CAL51 cells (Figure S4A,B), reciprocal endogenous co‐immunoprecipitation (Co‐IP) confirmed FBXO28‐MST1 binding in both cell lines (Figure 6I,J), and in vitro glutathione S‐transferase (GST) pull‐down assays using purified recombinant proteins demonstrated a direct interaction between FBXO28 and MST1 (Figure 6K,L). To determine whether FBXO28 broadly affects Hippo pathway core kinases or preferentially regulates MST1, we examined MST1, MST2, LATS1, and LATS2 after FBXO28 manipulation. FBXO28 knockdown increased MST1 protein levels in MDA‐MB‐231 cells, whereas FBXO28 overexpression reduced MST1 protein levels in CAL51 cells; in contrast, MST2, LATS1, and LATS2 were not substantially altered (Figure S4C). Consistently, endogenous FBXO28 immunoprecipitates specifically contained MST1, but not MST2, LATS1, or LATS2, although all four proteins were readily detected in the input fractions; IgG controls showed no detectable binding (Figure S4D,E). These data suggest that FBXO28 preferentially regulates and interacts with MST1 among the examined Hippo pathway core kinases. To delineate the interaction domains, we generated a series of FBXO28 deletion mutants based on Simple Modular Architecture Research Tool (SMART) and UniProt annotations (Figure 6M). Co‐IP experiments revealed that deletion of the C1 region abolished the interaction with full‐length MST1, whereas other truncations retained binding (Figure 6N,O). Similarly, MST1 deletion constructs showed that the M domain of MST1 was required for binding to FBXO28 (Figure 6P,Q). Together, these data indicate that the FBXO28 C1 region and the MST1 M region are required for FBXO28‐MST1 binding and that FBXO28 modulates MST1 protein stability in a proteasome‐dependent manner.

### 2.8 FBXO28 Promotes K48‐Linked Ubiquitination and Proteasomal Degradation of MST1

**FIGURE 7:**
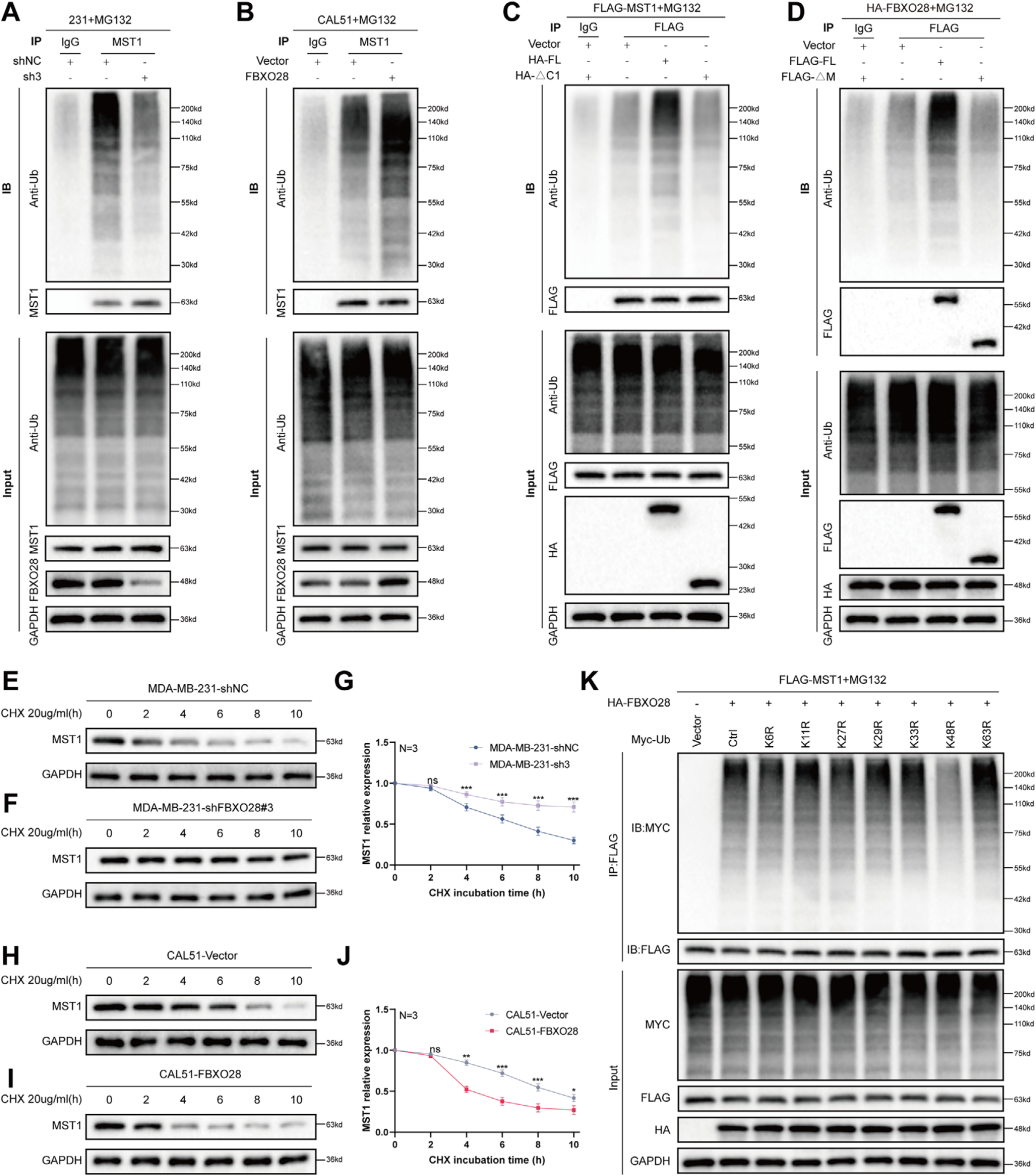
FBXO28 promotes K48‐linked ubiquitination and degradation of MST1. (A) MST1 ubiquitination in FBXO28‐knockdown MDA‐MB‐231 cells. (B) MST1 ubiquitination in FBXO28‐overexpressing CAL51 cells. (C) MST1 ubiquitination in cells expressing FBXO28 full‐length or deletion mutants. (D) MST1 ubiquitination in cells expressing MST1 full‐length or deletion mutants. (E–F) CHX chase assays in MDA‐MB‐231 shNC (E) and shFBXO28#3 (F) cells. (G) Quantification of MST1 protein stability in MDA‐MB‐231 cells. (H–I) CHX chase assays in CAL51 vector (H) and FBXO28‐overexpressing (I) cells. (J) Quantification of MST1 protein stability in CAL51 cells. (K) MST1 ubiquitination using lysine‐mutant ubiquitin constructs. ^*^ *p* < 0.05, ^**^ *p* < 0.01, ^***^ *p* < 0.001, ns, not significant.

To determine whether FBXO28 directly regulates MST1 ubiquitination, we first examined MST1 ubiquitination levels after manipulating FBXO28 expression (Figure 7). In MDA‐MB‐231 cells, FBXO28 knockdown markedly reduced MST1 ubiquitination (Figure 7A), whereas FBXO28 overexpression in CAL51 cells increased MST1 ubiquitination (Figure 7B). To further assess substrate specificity within the Hippo pathway, we examined whether FBXO28 knockdown affected the ubiquitination of MST2, LATS1, or LATS2. In contrast to the marked reduction in MST1 ubiquitination, FBXO28 knockdown did not substantially alter the ubiquitination levels of MST2, LATS1, or LATS2 in MDA‐MB‐231 cells (Figure S4F–H). These results further support that MST1 is the primary Hippo pathway kinase targeted by FBXO28‐mediated ubiquitination under our experimental conditions. Mapping analysis using FBXO28 and MST1 deletion mutants revealed that loss of the FBXO28 C1 domain or the MST1 M domain substantially impaired MST1 ubiquitination (Figure 7C,D), indicating that these regions are required for substrate recognition. To assess whether FBXO28 affects MST1 protein stability, cycloheximide (CHX) chase assays were performed. MST1 was more stable in FBXO28‐knockdown MDA‐MB‐231 cells compared with control cells (Figure 7E–G), whereas MST1 exhibited a shorter half‐life in FBXO28‐overexpressing CAL51 cells (Figure 7H–J). To determine the ubiquitin linkage type, MST1 ubiquitination was examined using lysine‐mutant ubiquitin constructs. Mutation of K48—but not other lysines—significantly reduced MST1 ubiquitination (Figure 7K and Figure S4I), indicating that FBXO28 catalyzes K48‐linked ubiquitin chains. Because FBXO proteins typically require the F‐box domain to bind SKP1 and assemble into the SCF E3 ligase complex, we next examined whether this interaction is preserved in FBXO28. Co‐immunoprecipitation analysis confirmed that FBXO28 interacted with SKP1 in an F‐box‐dependent manner: SKP1 was detected in full‐length FBXO28 (FBXO28‐FL) immunoprecipitates but not in the ΔF‐box mutant (Figure S4J), supporting its role as a functional SCF‐type E3 ligase. Complementary rescue assays further supported the ZBTB11–FBXO28–MST1 hierarchy. FBXO28 overexpression restored MST1 reduction in ZBTB11‐knockdown MDA‐MB‐231 and CAL51 cells, whereas FBXO28 knockdown restored MST1 expression in ZBTB11‐overexpressing cells (Figure S4K,L). In high‐ZBTB11 MDA‐MB‐231 and CAL51 cells, ectopic ZBTB11 did not further increase FBXO28 or reduce MST1, consistent with the limited gain‐of‐function phenotype. In contrast, ZBTB11 overexpression increased FBXO28 and reduced MST1 in low‐ZBTB11 MCF7 and SK‐BR‐3 cells, and FBXO28 knockdown reversed this effect. Together, these data demonstrate that FBXO28 acts downstream of ZBTB11 to promote K48‐linked MST1 ubiquitination and proteasomal degradation.

### 2.9 FBXO28‐MST1 Signaling Axis Modulates Hippo Pathway Activation, YAP/TAZ Nuclear Accumulation, and Transcriptional Output

**FIGURE 8:**
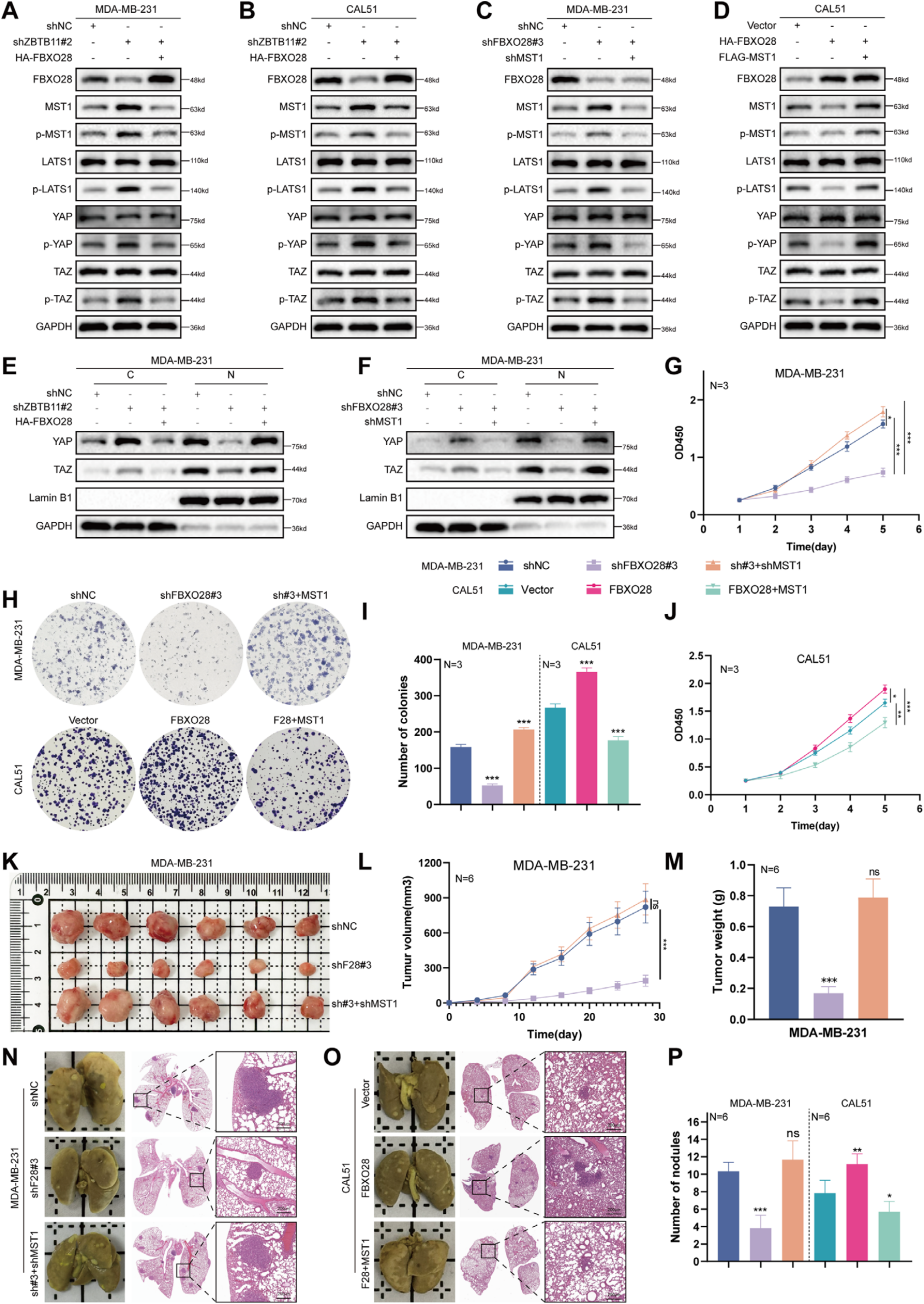
The FBXO28–MST1 axis regulates Hippo pathway activation and YAP/TAZ localization. (A‐B) Western blot analysis of Hippo pathway components in ZBTB11‐knockdown cells with or without FBXO28 re‐expression. (C) Western blot analysis of Hippo signaling in FBXO28‐knockdown cells with or without MST1 co‐silencing. (D) Western blot analysis of Hippo signaling in FBXO28‐overexpressing cells with or without MST1 co‐expression. (E) Nuclear–cytoplasmic distribution of YAP/TAZ in ZBTB11‐knockdown cells with or without FBXO28 re‐expression. (F) Nuclear–cytoplasmic distribution of YAP/TAZ in FBXO28‐knockdown cells with or without MST1 co‐silencing. (G–P) Proliferation, colony formation, migration, invasion, xenograft growth, and lung metastasis assays in FBXO28‐knockdown cells with MST1 co‐silencing or FBXO28‐overexpressing cells with MST1 re‐expression. ^*^ *p* < 0.05, ^**^ *p* < 0.01, ^***^ *p* < 0.001, ns, not significant.

To define how the ZBTB11‐FBXO28‐MST1 axis regulates Hippo pathway activity, we first examined core Hippo components under targeted perturbation of these molecules. In both MDA‐MB‐231 and CAL51 cells, ZBTB11 knockdown decreased FBXO28 expression and elevated MST1 and phospho‐MST1 levels, accompanied by increased phosphorylation of LATS1, YAP, and TAZ, whereas total LATS1/YAP/TAZ levels remained unchanged (Figure 8A,B). Re‐expression of FBXO28 reversed these changes, indicating that ZBTB11 suppresses Hippo signaling through FBXO28‐dependent MST1 regulation. Consistently, FBXO28 knockdown enhanced MST1 and phospho‐MST1 expression together with increased LATS1/YAP/TAZ phosphorylation, whereas simultaneous MST1 silencing restored Hippo pathway readouts toward near‐baseline levels (Figure 8C). Conversely, FBXO28 overexpression in CAL51 cells reduced MST1 and phospho‐MST1 while decreasing LATS1/YAP/TAZ phosphorylation, and MST1 co‐expression abolished these effects (Figure 8D). The efficiency of MST1 knockdown and overexpression was validated by RT‐qPCR and Western blotting (Figure S5A–D). In MCF7 and SK‐BR‐3 cells, ZBTB11 overexpression increased FBXO28 expression, reduced MST1 and phospho‐MST1 levels, and decreased LATS1/YAP/TAZ phosphorylation without markedly altering total LATS1/YAP/TAZ levels; these changes were reversed by FBXO28 knockdown (Figure S5G). These results indicate that ZBTB11‐mediated Hippo pathway suppression through FBXO28‐dependent MST1 regulation is not restricted to TNBC cells and is also observed in the examined non‐TNBC breast cancer cell backgrounds. To determine whether these biochemical changes influence YAP/TAZ transcriptional output, we evaluated their subcellular distribution. Nuclear‐cytoplasmic fractionation revealed that ZBTB11 or FBXO28 knockdown markedly reduced nuclear YAP/TAZ, while FBXO28 or MST1 re‐expression restored their nuclear accumulation (Figure 8E,F). The same regulatory pattern was confirmed in CAL51 cells (Figure S5E,F), demonstrating that FBXO28‐MST1 signaling governs YAP/TAZ nuclear localization downstream of ZBTB11. Consistent with altered YAP/TAZ localization, RT‐qPCR showed that FBXO28 knockdown reduced the expression of two representative YAP/TAZ‐TEAD target genes, CCN2 (CTGF) and CCN1 (CYR61), whereas MST1 co‐silencing restored their expression. Conversely, FBXO28 overexpression increased CCN2 and CCN1 expression, and MST1 re‐expression attenuated this induction (Figure S5H). In MCF7 and SK‐BR‐3 cells, ZBTB11 overexpression similarly induced CCN2 and CCN1 in an FBXO28‐dependent manner (Figure S5I). These results indicate that the ZBTB11‐FBXO28‐MST1 axis regulates not only Hippo pathway phosphorylation and YAP/TAZ nuclear accumulation, but also downstream YAP/TAZ transcriptional output. We next assessed the functional consequences of MST1 rescue on FBXO28‐driven tumor phenotypes. In MDA‐MB‐231 cells, FBXO28 depletion suppressed proliferation and colony formation, whereas MST1 co‐silencing restored these malignant phenotypes (Figure 8G–I). Conversely, FBXO28 overexpression in CAL51 cells enhanced proliferation, and MST1 co‐expression abrogated these effects (Figure 8I,J). In vivo, FBXO28 knockdown markedly reduced tumor growth and lung metastasis, while MST1 co‐silencing restored both tumorigenic and metastatic capacity (Figure 8K–N). Similarly, the protumorigenic effects of FBXO28 overexpression were suppressed by MST1 re‐expression (Figure 8O,P). Collectively, these findings establish MST1 as a key downstream effector mediating FBXO28‐driven Hippo pathway suppression and tumor progression.

### 2.10 The ZBTB11‐FBXO28‐MST1 Axis Predicts Patient Prognosis and Reveals Downstream YAP Dependency

**FIGURE 9:**
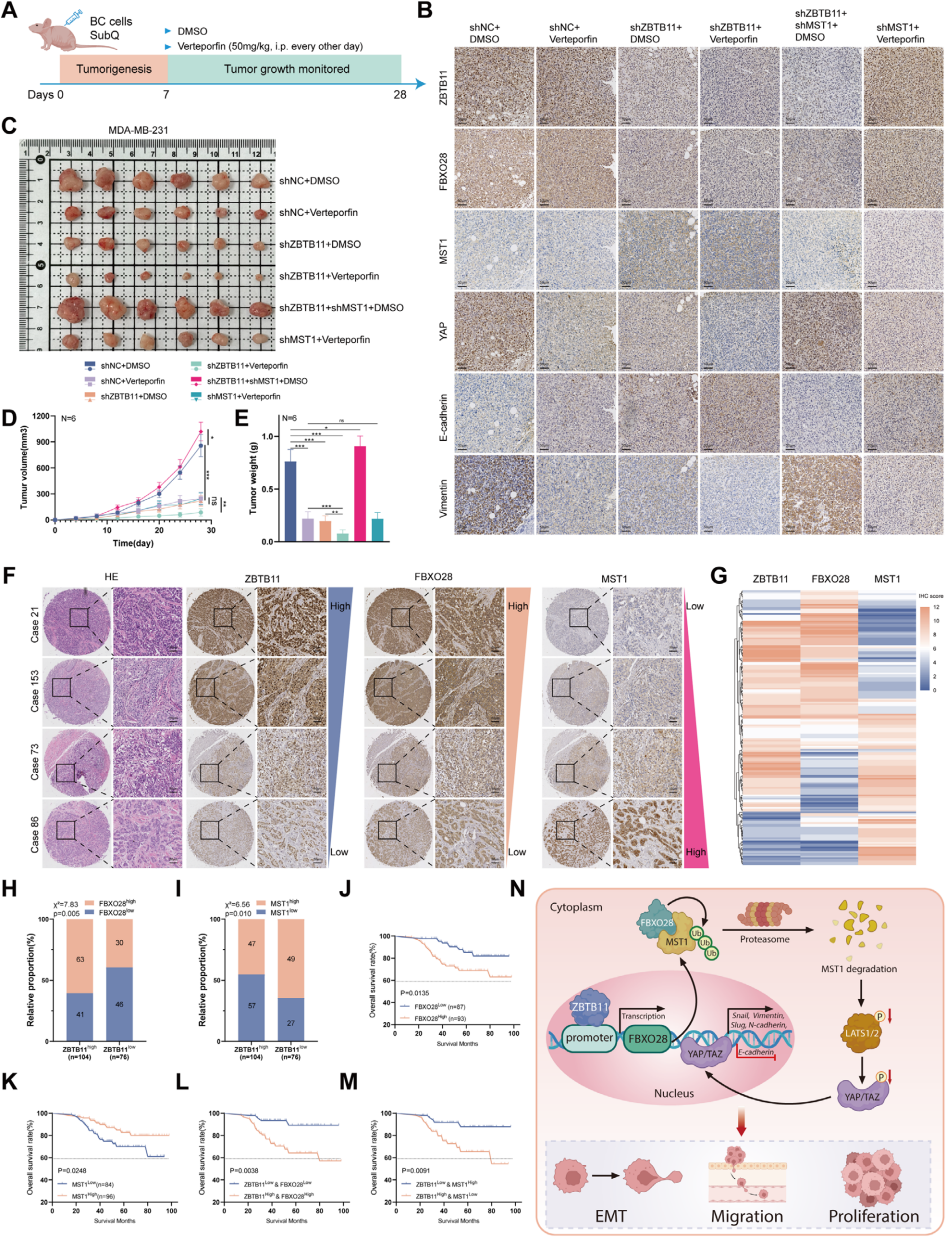
The ZBTB11–FBXO28–MST1 axis reveals downstream YAP dependency and predicts patient prognosis. (A) Schematic of xenograft treatment groups with or without Verteporfin. (B) Representative IHC staining of xenograft tissues for ZBTB11, FBXO28, MST1, YAP, E‐cadherin, and Vimentin. (C–E) Tumor images, growth curves, and final tumor weights in the indicated groups. (F) Representative TMA images showing ZBTB11, FBXO28, and MST1 expression. (G) Heatmap of protein expression across 180 breast cancer samples. (H–I) Associations of ZBTB11 with FBXO28 and MST1 based on IHC scoring. (J–M) Kaplan–Meier OS analyses of FBXO28, MST1, and combined biomarker signatures. (N) Schematic model of the ZBTB11–FBXO28–MST1–Hippo regulatory axis. ^*^ *p* < 0.05, ^**^ *p* < 0.01, ^***^ *p* < 0.001, ns, not significant.

To evaluate the functional relevance of the ZBTB11‐FBXO28‐MST1 axis in vivo, we performed xenograft experiments using MDA‐MB‐231 cells treated with Verteporfin, a commonly used inhibitor of YAP/TAZ‐TEAD transcriptional activity [39, 40, 41]. Both ZBTB11 knockdown and Verteporfin monotherapy significantly reduced tumor growth, and combined treatment exhibited the strongest inhibitory effect (Figure 9A–E). Silencing MST1 restored tumor growth in ZBTB11‐knockdown tumors, whereas Verteporfin suppressed tumor progression even in MST1‐deficient tumors, indicating that MST1 functions downstream of ZBTB11 and FBXO28, whereas Verteporfin acts downstream of MST1 within the Hippo‐YAP pathway. These experiments were designed to validate downstream YAP dependency of the ZBTB11‐FBXO28‐MST1 axis rather than to model direct therapeutic inhibition of ZBTB11 or FBXO28. IHC staining of xenograft tissues further confirmed the expected trends for ZBTB11, FBXO28, MST1, YAP, E‐cadherin, and Vimentin (Figure 9B). We next assessed the clinical relevance of this axis in a 180‐case breast cancer TMA cohort. Representative TMA images and IRS heatmap showed the expression patterns of ZBTB11, FBXO28, and MST1 (Figure 9F,G). Categorical analysis demonstrated that ZBTB11‐high tumors were significantly enriched for FBXO28‐high and MST1‐low cases (Figure 9H,I). Interobserver agreement for high/low classification was excellent, with Cohen's kappa values of 0.841, 0.867, and 0.811 for ZBTB11, FBXO28, and MST1, respectively (Table S3), and complete IRS data are provided in Data S5. Continuous IRS‐based scatter plots further confirmed a positive correlation between ZBTB11 and FBXO28 and negative correlations between ZBTB11 and MST1, as well as between FBXO28 and MST1 (Figure S5J–L).

Survival analyses showed that high FBXO28 expression predicted poorer OS, whereas high MST1 expression was associated with favorable OS (Figure 9J,K). Combined biomarker analysis further revealed that ZBTB11‐High/FBXO28‐High and ZBTB11‐High/MST1‐Low tumors had worse OS (Figure 9L,M). Consistently, DFS analyses showed that FBXO28‐High, MST1‐Low, and combined ZBTB11‐FBXO28 or ZBTB11‐MST1 signatures stratified patient risk more effectively than single markers alone (Figure S5M–T). Notably, the triple‐marker combination ZBTB11‐High/FBXO28‐High/MST1‐Low identified the subgroup with the poorest survival (Figure S5U,V). Together, these results support the clinical relevance of the ZBTB11‐FBXO28‐MST1 axis and suggest that this axis may serve as a prognostic biomarker and a candidate therapeutic vulnerability requiring further validation, particularly through future strategies directly targeting ZBTB11 or FBXO28.

## 3 Discussion

Metastasis remains the major cause of breast cancer‐related mortality, yet the transcriptional programs that connect metastatic progression with post‐translational pathway regulation remain incompletely understood [4, 5, 6]. In this study, we identified ZBTB11 as an oncogenic transcription factor upregulated in metastatic lymph node lesions and primary breast tumors and associated with poor patient outcomes. Although ZBTB11 was initially identified from metastatic lesions, its consistent elevation across paired tumor‐normal tissues and an independent TMA cohort suggests that ZBTB11 dysregulation is not restricted to late‐stage disease but may represent an early and sustained event during breast cancer progression. The mechanisms responsible for ZBTB11 upregulation remain unresolved. Potential contributors may include genomic amplification, enhancer activation, epigenetic remodeling, or oncogenic signaling. Given the role identified in this study for ZBTB11 in promoting EMT and regulating Hippo‐YAP/TAZ signaling through the FBXO28‐MST1 axis, future studies should determine whether ZBTB11‐high breast tumors exhibit altered sensitivity to chemotherapy, endocrine therapy, HER2‐targeted therapy, or YAP/TEAD‐directed interventions.

Functionally, ZBTB11 knockdown consistently suppressed proliferation, migration, invasion, tumor growth, and metastatic colonization in TNBC models. In contrast, further ZBTB11 overexpression did not enhance malignant phenotypes in MDA‐MB‐231 and CAL51 cells, likely reflecting a saturation or ceiling effect in high‐ZBTB11 backgrounds, a possibility consistent with previous observations that oncogenic transcription factor outputs can be dose‐dependent, threshold‐regulated, nonlinear, and context‐dependent [42, 43]. The gain‐of‐function assays in relatively low‐ZBTB11 MCF7 and SK‐BR‐3 cells support this interpretation, as ZBTB11 overexpression promoted proliferation, colony formation, EMT‐like marker changes, and migration/invasion in SK‐BR‐3 cells. Thus, the oncogenic output of ZBTB11 appears context dependent and influenced by basal activation of the downstream program. We also acknowledge that the ZBTB11 expression pattern observed in our cell line panel differs from the RNA‐seq‐ and MS‐based profiles reported in the Human Protein Atlas [38]. These differences may reflect platform‐ and normalization‐dependent differences between RNA‐seq/nTPM and qPCR, as well as between MS‐based nRPX and immunoblotting. Therefore, high‐ and low‐ZBTB11 models were selected based on RT‐qPCR and immunoblot validation in the same experimental system used for functional assays, while avoiding overgeneralization across all breast cancer cell lines.

Mechanistically, transcriptomic screening combined with transcription factor‐binding prediction identified FBXO28 as a key downstream effector of ZBTB11. Previous studies have implicated FBXO28 in cancer‐related processes through distinct molecular contexts, including SMARCC2 ubiquitination in pancreatic cancer, TGF‐β1/SMAD2/3 signaling in ovarian cancer, PFKFB4‐HIF‐1α signaling in glioblastoma, and MYC‐driven transcription in breast cancer [27, 28, 29, 30]. However, these studies did not identify MST1 as an FBXO28 substrate or connect FBXO28 to MST1‐dependent Hippo kinase regulation. Therefore, the conceptual advance of this study is not simply that FBXO28 participates in cancer progression, but that ZBTB11 transcriptionally activates FBXO28 to create a previously unrecognized regulatory route from oncogenic transcription to MST1 ubiquitination and Hippo pathway suppression. ZBTB11 bound the FBXO28 promoter and activated FBXO28 transcription, thereby linking transcriptional dysregulation to E3 ligase‐mediated control of Hippo signaling. Importantly, ZBTB11 overexpression increased FBXO28 expression in low‐ZBTB11 MCF7 and SK‐BR‐3 cells, and FBXO28 knockdown reversed ZBTB11‐induced EMT and malignant phenotypes. In addition, comprehensive loss‐ and gain‐of‐function experiments demonstrated that FBXO28 promoted proliferation, clonogenic growth, migration, invasion, and tumor growth, while FBXO28 re‐expression restored the malignant phenotypes suppressed by ZBTB11 knockdown. FBXO28 overexpression also enhanced proliferation and colony formation in MCF7 and SK‐BR‐3 cells and promoted migration and invasion in SK‐BR‐3 cells, supporting a tumor‐promoting role for FBXO28 in both TNBC cells and the examined non‐TNBC breast cancer cell backgrounds. These findings support FBXO28 as a critical functional effector of ZBTB11 and indicate that this regulatory relationship is not restricted to TNBC cells.

Our study further identifies MST1 as a previously unrecognized FBXO28 substrate. FBXO28 promoted K48‐linked MST1 ubiquitination and accelerated proteasomal degradation without altering MST1 mRNA levels. Mechanistically, FBXO28 directly interacted with MST1, and domain‐mapping experiments showed that the FBXO28 C1 region and the MST1 M region are required for FBXO28‐MST1 binding and MST1 ubiquitination. CHX chase assays confirmed that FBXO28 reduced MST1 protein stability, providing a post‐transcriptional mechanism for ZBTB11‐dependent MST1 suppression. Because the Hippo pathway contains closely related core kinases, we further examined whether FBXO28 broadly regulates MST2, LATS1, or LATS2. FBXO28 manipulation altered MST1 protein abundance but did not substantially affect MST2, LATS1, or LATS2 levels. Moreover, endogenous FBXO28 specifically co‐immunoprecipitated with MST1 but not MST2, LATS1, or LATS2, and FBXO28 knockdown did not markedly change the ubiquitination levels of MST2, LATS1, or LATS2. These results indicate that, under our experimental conditions, FBXO28 preferentially targets MST1 rather than broadly regulating multiple Hippo core kinases. While previous work has shown that MST1 can be ubiquitinated by RNF6 in breast cancer, our study demonstrates, to our knowledge for the first time, that an F‐box protein, FBXO28, regulates MST1 stability and Hippo signaling downstream of ZBTB11. This finding distinguishes our work from previous FBXO28 studies focused on other substrates or signaling contexts and from broader studies of Hippo ubiquitination that did not implicate FBXO28.

Consistent with MST1 destabilization, FBXO28 suppressed Hippo pathway activation, reduced phosphorylation of LATS1, YAP, and TAZ, and promoted YAP/TAZ nuclear accumulation. Rescue experiments using MST1 depletion or re‐expression further confirmed MST1 as a key downstream mediator of FBXO28‐driven Hippo pathway regulation and tumor phenotypes. Beyond YAP/TAZ localization, RT‐qPCR analysis of representative YAP/TAZ‐TEAD target genes demonstrated that this axis regulates YAP/TAZ transcriptional output. FBXO28‐MST1 manipulation altered the expression of CCN2 (CTGF) and CCN1 (CYR61), and ZBTB11 overexpression induced these targets in MCF7 and SK‐BR‐3 cells in an FBXO28‐dependent manner. Nevertheless, YAP/TAZ signaling can exert subtype‐dependent biological effects in breast cancer, particularly between ER‐positive and TNBC contexts. Therefore, although the ZBTB11‐FBXO28‐MST1 mechanism was observed in both TNBC and the examined non‐TNBC cell backgrounds, its phenotypic consequences should be interpreted as context dependent rather than universally identical across all breast cancer subtypes.

Pharmacologic inhibition with Verteporfin further supported downstream YAP/TAZ dependency of the ZBTB11‐FBXO28‐MST1 cascade. However, Verteporfin was originally developed as a photosensitizer and has reported YAP/TAZ‐independent activities, including proteotoxic stress [39, 40, 41]. Therefore, the Verteporfin experiments should be interpreted primarily as downstream pathway validation and as a potential strategy requiring further validation, rather than direct evidence of clinical efficacy or direct targeting of ZBTB11/FBXO28. Clinically, the 180‐case TMA cohort showed concordant ZBTB11 and FBXO28 expression, inverse MST1 expression, and poorer outcomes in patients with combined ZBTB11‐high/FBXO28‐high/MST1‐low signatures. These clinical data provide in situ evidence consistent with the proposed regulatory axis and suggest that combined marker assessment may improve prognostic stratification compared with single‐marker analysis.

Several limitations should be noted. Although validation was expanded beyond TNBC by including MCF7 and SK‐BR‐3 cells, additional ER‐positive, HER2‐positive, patient‐derived organoid, and patient‐derived xenograft models are needed to define subtype‐specific generalizability. Although truncation mutants mapped the FBXO28 C1 region and MST1 M region as required interaction regions, the precise amino acid residues mediating this interaction remain undefined and require future structure‐guided mutagenesis and high‐resolution structural studies. Moreover, we did not perform therapeutic‐style in vivo targeting of ZBTB11 or FBXO28 after tumor establishment using systemically delivered siRNA, antisense oligonucleotides, or selective small‐molecule inhibitors. Direct targeting of ZBTB11 transcriptional activity, FBXO28 E3 ligase function, or MST1 stabilization will require dedicated preclinical optimization. In summary, our findings define a ZBTB11‐FBXO28‐MST1 transcription‐to‐ubiquitination cascade that suppresses Hippo signaling, promotes YAP/TAZ transcriptional activity, and drives EMT and metastatic progression, highlighting this axis as a biomarker and candidate therapeutic vulnerability requiring further validation.

## 4 Conclusion

In conclusion, this study identifies ZBTB11 as a functionally important oncogenic transcription factor that drives breast cancer progression. Mechanistically, ZBTB11 directly activates FBXO28, which in turn induces K48‐linked ubiquitination and proteasomal degradation of MST1, resulting in Hippo pathway suppression and YAP/TAZ‐associated EMT and metastatic traits. Clinically, high ZBTB11 and FBXO28 expression combined with low MST1 levels define a patient subset with significantly worse survival outcomes. These findings uncover a previously unrecognized transcription‐to‐ubiquitination cascade controlling Hippo signaling and highlight the ZBTB11‐FBXO28‐MST1 axis as a prognostic biomarker and candidate therapeutic vulnerability that warrants further validation in aggressive breast cancer.

## 5 Experimental Section

### 5.1 Patients and Sample Collection

A total of 180 breast cancer tissue specimens were collected from Northern Jiangsu People's Hospital Affiliated to Yangzhou University between January 2016 and July 2022 and used to construct tissue microarrays (TMAs) containing primary tumor tissues and matched adjacent non‐tumorous tissues. TMAs were produced by Shanghai Xinchao Biotechnology Co., Ltd. An additional independent cohort of 20 breast cancer tissues was used for IHC validation. A total of 5 paired primary tumors and metastatic lymph nodes were collected for RNA sequencing, 35 paired breast cancer and adjacent normal tissues for RT‐qPCR, and eight paired samples for Western blotting.

### 5.2 Ethics Approval and Consent to Participate

All procedures involving human participants were performed in accordance with the Declaration of Helsinki. The collection and use of human breast cancer specimens were approved by the Ethics Committee of the Northern Jiangsu People's Hospital Affiliated to Yangzhou University (Approval No. 2024ky381). Written informed consent was obtained from all participants prior to sample collection. All animal experiments were conducted in accordance with the Animal Research: Reporting of In Vivo Experiments (ARRIVE) guidelines and were approved by the Yangzhou University Laboratory Animal Ethics Committee (Approval No. 202412022).

### 5.3 Cell Lines and Lentivirus Infection

Human breast cancer cell lines MDA‐MB‐231, CAL51, HCC1937, SUM159PT, SK‐BR‐3, MDA‐MB‐453, MCF7, and T47D were obtained from the Cell Bank of the Chinese Academy of Sciences. All cell lines were authenticated by short tandem repeat (STR) profiling and confirmed mycoplasma‐free. Cells were cultured in Dulbecco’s modified Eagle’s medium (DMEM) or Roswell Park Memorial Institute 1640 (RPMI‐1640) medium supplemented with 10% fetal bovine serum and 1% penicillin‐streptomycin at 37°C with 5% CO_2_. Lentiviral shRNA and overexpression vectors were generated by OBiO Technology (Shanghai) Corp., Ltd. Stable cell lines were established by lentiviral infection with 8 µg/mL polybrene followed by puromycin selection. Knockdown or overexpression efficiency was confirmed by RT‐qPCR and/or Western blotting. Relevant sequences are listed in Table S5.

### 5.4 Western Blotting and Reverse Transcription Quantitative PCR

Cells or tumor tissues were lysed in radio immunoprecipitation assay (RIPA) buffer supplemented with phenylmethylsulfonyl fluoride (PMSF) and phosphatase inhibitors. Equal amounts of protein were separated by sodium dodecyl sulfate‐polyacrylamide gel electrophoresis (SDS‐PAGE), transferred onto polyvinylidene difluoride (PVDF) membranes, incubated with primary and horseradish peroxidase (HRP)‐conjugated secondary antibodies, and detected using enhanced chemiluminescence (ECL). Band intensities were quantified using ImageJ. Antibody information is provided in Table S4.

For reverse transcription quantitative PCR (RT‐qPCR), total RNA was extracted using TRIzol reagent, reverse‐transcribed into complementary DNA (cDNA), and analyzed using SYBR Green Master Mix. Genes with cycle threshold (Ct) values >30 were excluded from analysis. Relative mRNA expression was calculated using the 2^−ΔΔCt method with GAPDH as the internal control. Primer sequences are listed in Table S6.

### 5.5 Cell Proliferation, Migration, and Invasion Assay

For the colony formation assay, 1000 cells were seeded into six‐well plates and cultured for 14 days. Colonies were fixed with 3% paraformaldehyde for 30 min, stained with 0.1% crystal violet for 30 min, photographed, and quantified. Cell proliferation was assessed using the CCK‐8 assay (Beyotime, C0041) following the manufacturer's instructions. Briefly, CCK‐8 solution was added to each well, incubated for the indicated time, and absorbance was measured at 450 nm. Cell migration and invasion were evaluated using Transwell chambers (24‐well format; Corning) with or without Matrigel coating, respectively. A total of 1 × 10^5 cells in serum‐free medium were added to the upper chamber, while 800 µL of complete medium was placed in the lower chamber as a chemoattractant. After 24 h (migration) or 36–48 h (invasion), cells on the lower membrane surface were fixed with 3% paraformaldehyde, stained with 0.1% crystal violet, imaged, and counted under a microscope.

### 5.6 RNA Sequencing and Data Analysis

To identify molecular determinants associated with breast cancer progression and metastasis, transcriptome sequencing (RNA‐seq) was performed on five paired primary breast tumors and matched metastatic lymph nodes (all T3N1). Total RNA was extracted and submitted to OBiO Technology (Shanghai) Corp., Ltd. for RNA‐seq and differential gene expression analysis. Similarly, MDA‐MB‐231‐shNC and MDA‐MB‐231‐shZBTB11 cells (three biological replicates each) were subjected to RNA‐seq to characterize transcriptional alterations associated with ZBTB11 depletion.

### 5.7 Immunohistochemistry (IHC)

IHC was performed on formalin‐fixed, paraffin‐embedded tissue sections or TMAs. Sections were deparaffinized, rehydrated, subjected to heat‐induced antigen retrieval, blocked, incubated with primary antibodies, and visualized using HRP‐conjugated secondary antibodies and 3,3′‐diaminobenzidine (DAB). Slides were counterstained with hematoxylin.

IHC staining was independently evaluated by two experienced pathologists blinded to clinical information. The immunoreactive score (IRS) was calculated as the product of the proportion score (P‐score: 0–4) and staining intensity score (I‐score: 0–3), ranging from 0 to 12. The median IRS value of 7 was used as the cut‐off, with IRS ≥ 7 defined as high expression and IRS < 7 as low expression. Discrepant scores were resolved by consensus discussion. Interobserver agreement was quantified using Cohen's kappa (Table S3). Complete IRS scores and high/low classifications for all 180 samples are provided in Data S5.

### 5.8 Nucleus‐Cytoplasmic Fractionation Assay

Nuclear and cytoplasmic proteins were isolated using a commercial extraction kit (Beyotime, P0027) according to the manufacturer's protocol. Briefly, cells were collected, resuspended in the supplied cytoplasmic extraction buffer, and centrifuged to separate the cytoplasmic fraction. The nuclear pellet was lysed in nuclear extraction buffer supplemented with dithiothreitol (DTT) and protease inhibitors, with brief sonication when necessary. After centrifugation at 14 000 × g for 10 min at 4°C, the supernatant containing nuclear proteins was collected for analysis. Fraction purity was verified by Western blotting using Lamin B1 and GAPDH as nuclear and cytoplasmic markers, respectively.

### 5.9 Chromatin Immunoprecipitation (ChIP) Assays

Chromatin immunoprecipitation was performed using the SimpleChIP Plus Sonication Chromatin IP Kit (Cell Signaling Technology, #56383S) according to the manufacturer's instructions. Briefly, approximately 1 × 10^7 breast cancer cells stably expressing FLAG‐ZBTB11 or FLAG‐ZBTB11‐ΔZnF were cross‐linked with 1% formaldehyde for 10 min and quenched with 0.125 m glycine. Cells were lysed, and chromatin was sheared by sonication to 200–1000 bp fragments. For each IP, 25 µg of chromatin was incubated overnight at 4°C with anti‐FLAG antibody or normal mouse IgG (Beyotime, A7028). Immune complexes were captured with protein G magnetic beads, washed, and eluted. Cross‐links were reversed at 65°C for 2 h, and DNA was purified using the provided spin columns. Enrichment of FLAG‐ZBTB11 at the FBXO28 promoter was quantified by ChIP‐qPCR using primers listed in Table S6.

### 5.10 Transcription Factor‐Target Gene Prediction

ZBTB11 targets were predicted using TF‐Target‐Finder [44], which integrates MotifMap, hTFtarget, KnockTF, TRRUST, Cistrome DB, ENCODE, and JASPAR. Additional predictions were obtained from GTRD [45] and ChIP‐Atlas [46], and overlapping genes were considered putative targets. The ZBTB11 motif and FBXO28 promoter binding sites were analyzed using the JASPAR database [47].

### 5.11 Dual Luciferase Reporter Assay

Wild‐type or mutant FBXO28 promoter fragments were cloned into the pGL3 firefly luciferase reporter vector. Cells were seeded in 24‐well plates at a density of 1 × 10^5 cells per well and co‐transfected with 0.7 µg of the pGL3 reporter plasmid and 0.6 µL of the thymidine kinase promoter‐driven Renilla luciferase control plasmid pRL‐TK using KeygenMAX 3000 transfection reagent (KeyGEN BioTECH, KGA9705‐1.5). After 48 h, cells were lysed, and luciferase activities were measured using the Dual‐Glo Luciferase Assay System (Promega, E2920) on a multimode microplate reader (Molecular Devices, M3). Firefly luciferase activity was normalized to Renilla luciferase activity, and relative luciferase activity was calculated as the Firefly/Renilla ratio.

### 5.12 Coimmunoprecipitation (CO‐IP) and LC‐MS/MS

Co‐immunoprecipitation was performed using the Co‐IP Kit (Beyotime, P2179S). Cell lysates were incubated with the indicated primary antibodies or control IgG, followed by protein A/G agarose beads. Immunoprecipitates were washed, eluted, and analyzed by Western blotting.

For protein identification, immunoprecipitated samples were analyzed by LC‐MS/MS at OBiO Technology (Shanghai) Corp., Ltd. using a timsTOF Pro mass spectrometer coupled with nanoElute high‐performance liquid chromatography (HPLC). Proteins were digested with trypsin with up to two missed cleavages allowed. Carbamidomethylation of cysteine was set as a fixed modification, and oxidation of methionine was set as a variable modification. Peptide mass tolerance was set at 20 ppm and fragment mass tolerance at 0.1 Da. Raw data were processed using MaxQuant (version 1.6.14) with the UniProt Homo sapiens database (version 20250111). Protein and peptide identifications were filtered at a false discovery rate (FDR) of <1%, and LFQ and intensity‐based absolute quantification (iBAQ) quantifications were enabled. Candidate FBXO28‐interacting proteins were prioritized based on enrichment over IgG control, peptide number, unique peptide number, sequence coverage, and LFQ/iBAQ intensity. Full LC‐MS/MS identification data are provided in Data S4.

### 5.13 Immunofluorescence

Cells were seeded on glass coverslips, fixed with 4% paraformaldehyde for 15 min, and permeabilized with 0.1% Triton X‐100 for 10 min. After blocking with 10% goat serum for 1 h, the cells were incubated with appropriate primary antibodies overnight at 4 °C, followed by species‐specific Alexa Fluor‐conjugated secondary antibodies (Alexa Fluor 488 or 555) for 1 h at room temperature in the dark. Nuclei were counterstained with 4′,6‐diamidino‐2‐phenylindole (DAPI) (Beyotime, C1006), and coverslips were mounted with antifade mounting medium. Images were captured using a confocal laser scanning microscope under identical exposure settings for all groups.

### 5.14 In Vivo Orthotopic Mammary Fat Pad Xenograft and Lung Metastasis Models

Nude mice (female, 4 weeks old, 15–21 g) were obtained from the Translational Medical Center of Yangzhou University and maintained under specific pathogen‐free conditions. Mice were randomly assigned to the indicated experimental and treatment groups as detailed in the Results section and figure legends. For the orthotopic mammary fat pad xenograft model, 5 × 10^6 breast cancer cells suspended in phosphate‐buffered saline (PBS) were injected into the right inguinal (fourth) mammary fat pad of each mouse (six mice per group). Tumor size was measured every 3 days using digital calipers, and tumor volume was calculated as (length × width^2)/2. After 4 weeks, mice were euthanized, and tumors were excised and weighed. For the experimental lung metastasis model, 1 × 10^6 cells suspended in 100–200 µL PBS were injected into the lateral tail vein. After 6 weeks, lungs were excised, briefly immersed in Bouin's solution to visualize metastatic nodules, photographed, and processed for paraffin embedding and haematoxylin‐eosin (H&E) staining. For drug‐treatment assays, Verteporfin (50 mg/kg in 150 µL vehicle; Shanghai Aladdin Bio‐Chem) was administered intraperitoneally every other day. Mice were monitored daily for general health, distress, and body weight. All animal studies were approved by the Animal Utilization Committee of Yangzhou University.

### 5.15 Bimolecular Fluorescence Complementation (BiFC) Assay

Bimolecular fluorescence complementation (BiFC) was performed to examine the interaction between FBXO28 and MST1 in breast cancer cells. FBXO28 was cloned into the VC155 vector, and MST1 was fused to the VN173 fragment using standard molecular cloning procedures. The indicated plasmids were transfected into MDA‐MB‐231 and CAL51 cells using Lipofectamine 2000 according to the manufacturer's instructions. After 24 h, fluorescence signals were assessed using a fluorescence microscope. Restored fluorescence indicated the reconstitution of the split fluorescent protein, reflecting a physical interaction between FBXO28 and MST1. Empty vectors and single‐plasmid transfections (FBXO28‐VC155 alone or MST1‐VN173 alone) served as negative controls to confirm signal specificity.

### 5.16 Construction of Domain Mutants

Full‐length coding sequences of ZBTB11, FBXO28 and MST1 were obtained from the UniProt database, and domain boundaries were determined using the Simple Modular Architecture Research Tool (SMART) database. Based on the predicted structural features, a series of domain‐deleted mutants were designed to map the regions responsible for transcriptional activity and protein‐protein interactions. For ZBTB11, a zinc finger‐deleted mutant (ZBTB11‐ΔZnF, deletion of amino acids (aa) 569–937) was generated to eliminate the C‐terminal zinc finger cluster. For FBXO28, four truncation mutants were constructed according to the functional domain organization: an N‐terminal deletion mutant lacking aa 1–60 (FBXO28‐ΔN), an F‐box‐deleted mutant lacking aa 61–109 (FBXO28‐ΔF‐box), and two C‐terminal truncation mutants corresponding to aa 110–327 (FBXO28‐ΔC1) and aa 328–368 (FBXO28‐ΔC2), respectively. For MST1, three domain mutants were generated based on its modular organization: an N‐terminal kinase‐domain deletion mutant (MST1‐ΔN, aa 1–281), a middle‐region deletion mutant (MST1‐ΔM, aa 282–432), and a C‐terminal SARAH‐domain deletion mutant (MST1‐ΔC, aa 433–487). All wild‐type and mutant fragments were synthesized and subcloned into mammalian expression vectors carrying Flag or HA epitope tags as indicated. Plasmid construction was performed by OBiO Technology (Shanghai) Corp., Ltd.

### 5.17 Glutathione S‐Transferase (GST) Pull‐Down Assay

The pGEX‐4T‐1‐MST1 and pET24a‐HA‐FBXO28 plasmids were transformed into Escherichia coli BL21 (DE3) to express GST‐tagged MST1 (GST‐MST1) and HA‐tagged FBXO28 (HA‐FBXO28), respectively. Bacterial cells were harvested, lysed by sonication, and centrifuged to obtain clarified lysates. GST‐MST1 was immobilized onto BeyoGoldTM GST‐tag Purification Resin (Beyotime, P2253) according to the manufacturer's protocol, whereas HA‐FBXO28 was purified using anti‐HA affinity resin under standard conditions. The GST‐MST1‐bound resin was washed with GST pull‐down binding buffer (50 mm Tris‐HCl, 200 mm NaCl, 10 mm MgCl_2_, 1 mm ethylenediaminetetraacetic acid (EDTA), 1% Nonidet P‐40 (NP‐40), 1 mm DTT, pH 8.0) and incubated with purified HA‐FBXO28 at 4°C for 4 h with rotation. After extensive washing, bound proteins were eluted and analyzed by SDS‐PAGE followed by immunoblotting. For the reciprocal assay, GST‐tagged FBXO28 (GST‐FBXO28) and FLAG‐tagged MST1 (FLAG‐MST1) were prepared using the same procedures. The pull‐down was performed symmetrically by incubating GST‐FBXO28 resin with purified FLAG‐MST1.

### 5.18 Ubiquitination Assay and Cycloheximide Chase Assay

For in vivo ubiquitination assays, cells were transfected with the indicated expression or shRNA constructs. Twenty‐four hours after transfection, cells were treated with MG132 (10 µM) for 6 h and then lysed in IP lysis buffer containing 1% SDS. Lysates were boiled for 10 min, diluted 10‐fold with non‐SDS lysis buffer, and subjected to immunoprecipitation with anti‐MST1 or anti‐FLAG/HA antibodies as indicated. The ubiquitination levels of MST1 were determined by Western blotting with anti‐ubiquitin or anti‐HA antibodies. To evaluate MST1 protein stability, cells were treated with cycloheximide (CHX, 20 µg/mL) for the indicated time periods (0, 2, 4, 6, 8, 10 h). At each time point, cells were harvested and lysed for Western blotting. MST1 band intensities were quantified using ImageJ and normalized to GAPDH, and the relative protein levels were plotted over time to estimate half‐life.

### 5.19 Statistics and Reproducibility

All experiments were performed independently at least three times unless otherwise specified. Data are presented as mean ± standard deviation (SD). Comparisons between two groups were performed using the unpaired two‐tailed Student's t‐test or Wilcoxon signed‐rank test, as appropriate. Multiple‐group comparisons were analyzed using one‐way analysis of variance (ANOVA) followed by post‐hoc pairwise testing. Pearson's correlation analysis was used for mRNA expression correlations. Spearman's correlation analysis was used to assess correlations among continuous IRS values of ZBTB11, FBXO28, and MST1 in the TMA cohort, and chi‐square tests were used for categorical IHC expression groups. Interobserver agreement for IHC high/low classification was assessed using Cohen's kappa.

OS and DFS were analyzed using the Kaplan–Meier method with log‐rank testing. In the TMA cohort, patients were stratified into high‐ and low‐expression groups according to the median IRS value. Univariate and multivariate Cox proportional hazards regression analyses were performed to evaluate prognostic factors. Variables examined in univariate analyses included ZBTB11 expression, age, tumor diameter, histological grade, TNM stage, ER status, PR status, HER2 status, and Ki‐67 index. Variables with p < 0.05 in univariate analysis were included in the multivariate Cox model. Molecular subtype was not included as a separate covariate to avoid collinearity with ER, PR, and HER2 status, and treatment regimen was not included because complete treatment information was not available for all cases in the TMA cohort. The proportional hazards assumption was assessed using Schoenfeld residuals. Cases with missing clinicopathological or follow‐up information were excluded from the corresponding analyses. HRs, 95% CIs, and *p* values are reported in Table S2. Statistical analyses were performed using GraphPad Prism 8.0.2, SPSS 21.0, and R 4.4.2. A two‐sided *p* < 0.05 was considered statistically significant. In figure legends, statistical significance is indicated as ^*^ *p* < 0.05, ^**^ *p* < 0.01, ^***^ *p* < 0.001, ns, not significant.

## Author Contributions

**An Xu**, **Xiang‐Nan Xu**, and **De‐Yuan Fu** conceived the study, designed and performed experiments, analyzed data, and wrote the manuscript. **Xiao Huang**, **Zhou Luo**, and **Chun‐Lian Li** performed experiments and analyzed data. **Yang Du**, **Cheng Yan**, **Xiao‐Jie Yu**, **Jian‐Wen Wang**, and **Long‐Di Yao** contributed to conceptualization and provided supervision. De‐Yuan Fu supervised the project, edited the manuscript, and provided final approval. All authors reviewed and approved the final manuscript.

## Funding

The National Natural Science Foundation of China (No.82072909); the Postgraduate Research and Practice Innovation Program of Jiangsu Province (No. SJCX24_2343).

## Conflicts of Interest

The authors declare no competing interests.

## Supporting information




**Supporting File 1**: advs76618‐sup‐0001‐SuppMat.docx.

**Supporting File 2**: advs76618‐sup‐0002‐data.zip.

## Data Availability

The data supporting the findings of this study are available from the corresponding author upon reasonable request. Available original Western blot source images corresponding to the immunoblots shown in the manuscript are provided in Supplementary Data S6. For experiments in which full‐membrane scans could not be retrieved, the available target‐protein strip or region source images are provided with annotations.
