# Supplementary material for: ZBTB11 Promotes Breast Cancer Progression by Activating FBXO28‐Mediated MST1 Degradation and Suppressing Hippo Signaling
Source: Adv Sci (Weinh). 2026 Jul 20:e76618. Online ahead of print. doi: 10.1002/advs.76618 (PMC13383149; doi:10.1002/advs.76618)
Supplement: Supplementary file 1 — Supporting File 1: advs76618‐sup‐0001‐SuppMat.docx. [file ADVS-9999-e76618-s002.docx]

Supplementary Materials for

ZBTB11 promotes breast cancer progression by activating FBXO28-mediated MST1 degradation and suppressing Hippo signaling

An Xu^1^†, Xiang-Nan Xu^1^†, Xiao Huang^1^, Zhou Luo^1^, Chun-Lian Li^1^, Yang Du^1^, Cheng Yan^1^, Xiao-Jie Yu^1^, Jian-Wen Wang^2^, Long-Di Yao^3^, De-Yuan Fu^1^*

*^1^**Department of General Surgery, Northern Jiangsu People’s Hospital Affiliated to Yangzhou University, Yangzhou, Jiangsu Province, China.*

*^2^Department of Thyroid and Breast Surgery, Xinghua People’s Hospital Affiliated to Yangzhou University, Xinghua, Jiangsu Province, China*

*^3^Department of Thyroid and Breast Surgery, Changxing Hospital of Traditional Chinese Medicine, Huzhou, Zhejiang Province, China.*

†These authors contributed equally to this work.

*Corresponding author: De-Yuan Fu ( fdy1003@163.com)

SUPPLEMENTARY MATERIALS

Supplementary Figures and Figure Legends


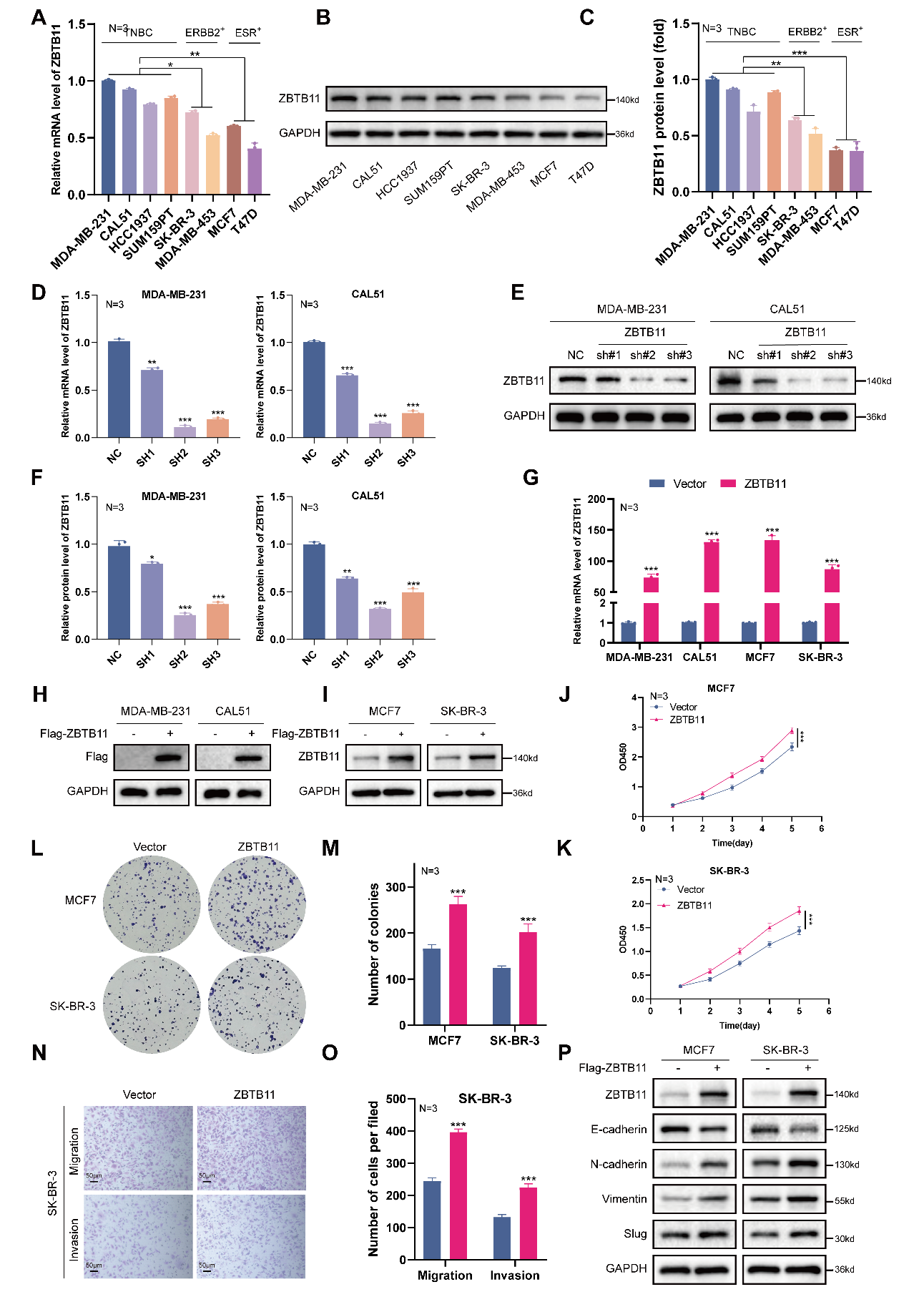


**Supplementary Figure S1**. ZBTB11 expression in the breast cancer cell line panel used in this study and functional validation of ZBTB11 knockdown and overexpression models.

(A) ZBTB11 mRNA expression across breast cancer cell lines. (B) Western blot detection of ZBTB11 protein levels in breast cancer cell lines. (C) Quantification of ZBTB11 protein expression. (D) qPCR analysis of ZBTB11 knockdown efficiency in MDA-MB-231 and CAL51 cells. (E) Western blot analysis of ZBTB11 knockdown in MDA-MB-231 and CAL51 cells. (F) Quantification of ZBTB11 protein levels after knockdown. (G) qPCR analysis of ZBTB11 mRNA levels in vector and ZBTB11-overexpression cells. (H) Western blot validation of FLAG-tagged ZBTB11 overexpression in MDA-MB-231 and CAL51 cells using an anti-FLAG antibody. (I) Western blot validation of ZBTB11 overexpression in MCF7 and SK-BR-3 cells using an anti-ZBTB11 antibody. (J, K) CCK-8 proliferation assays of ZBTB11-overexpressing MCF7 (J) and SK-BR-3 (K) cells. (L) Representative colony formation images of vector and ZBTB11-overexpression MCF7 and SK-BR-3 cells. (M) Quantification of colony formation assays. (N) Representative Transwell migration and invasion images of vector and ZBTB11-overexpression SK-BR-3 cells. (O) Quantification of Transwell migration and invasion assays. (P) Western blot analysis of EMT-related proteins in vector and ZBTB11-overexpression MCF7 and SK-BR-3 cells. *P < 0.05, **P < 0.01, ***P < 0.001, ns, not significant.


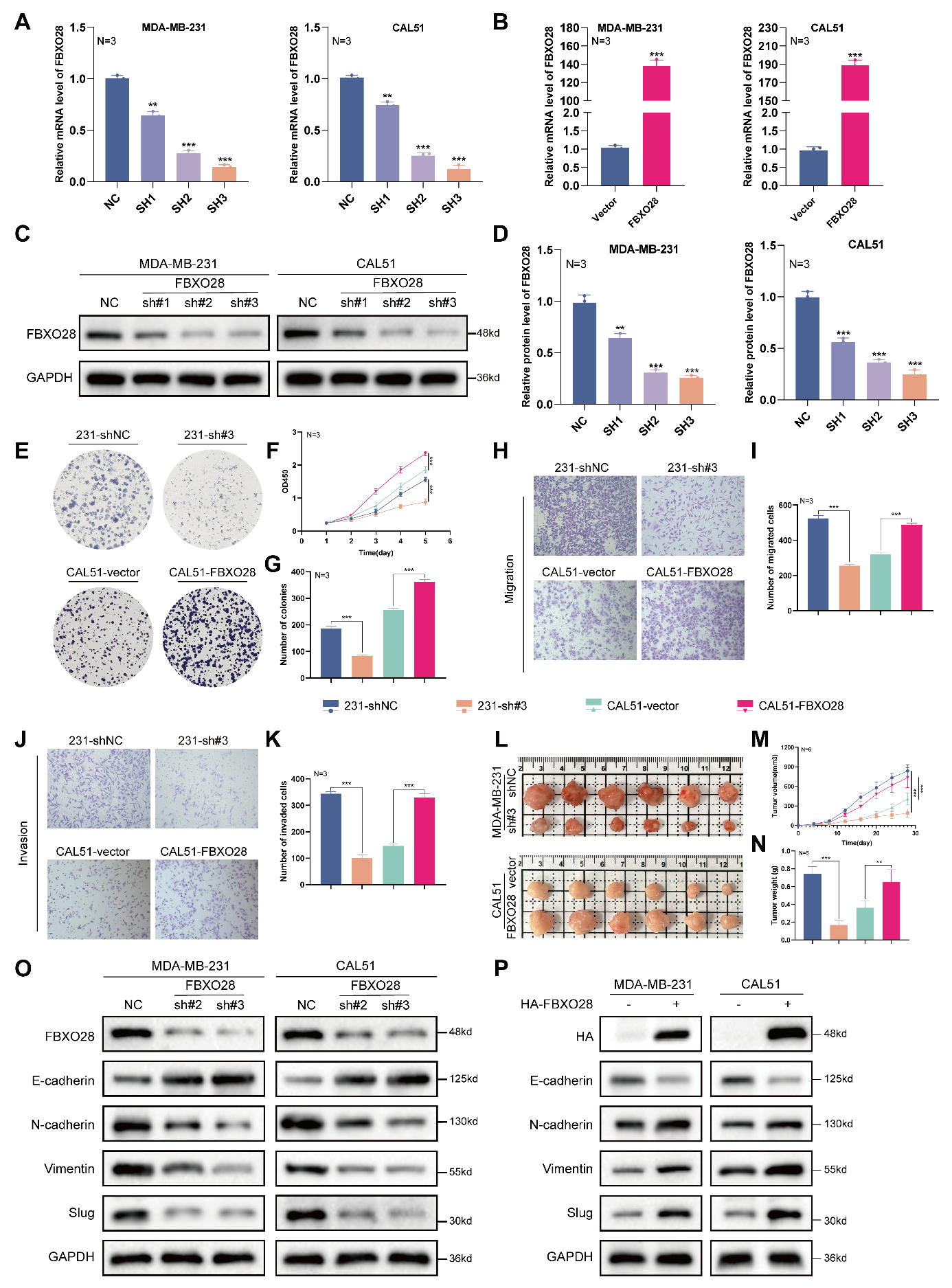


**Supplementary Figure S2**. Functional characterization of FBXO28 knockdown and overexpression in breast cancer cells.

(A) qPCR analysis of FBXO28 knockdown efficiency in MDA-MB-231 and CAL51 cells. (B) qPCR analysis of FBXO28 overexpression in MDA-MB-231 and CAL51 cells. (C) Western blot detection of FBXO28 protein levels after knockdown. (D) Quantification of FBXO28 protein levels. (E) Colony formation assays of FBXO28 knockdown in MDA-MB-231 and FBXO28 overexpression in CAL51 cells. (F) Quantification of colony formation. (G) CCK-8 proliferation assays of FBXO28 knockdown and overexpression cells. (H) Transwell migration assays of knockdown and overexpression groups. (I) Quantification of migration assays. (J) Transwell invasion assays of knockdown and overexpression groups. (K) Quantification of invasion assays. (L) Orthotopic mammary fat pad xenograft images of FBXO28 knockdown (MDA-MB-231) and overexpression (CAL51) models. (M) Tumor growth curves. (N) Tumor weight measurements. (O) Western blot analysis of EMT-related proteins after FBXO28 knockdown. (P) Western blot analysis of EMT-related proteins after FBXO28 overexpression. *P < 0.05, **P < 0.01, ***P < 0.001, ns, not significant.


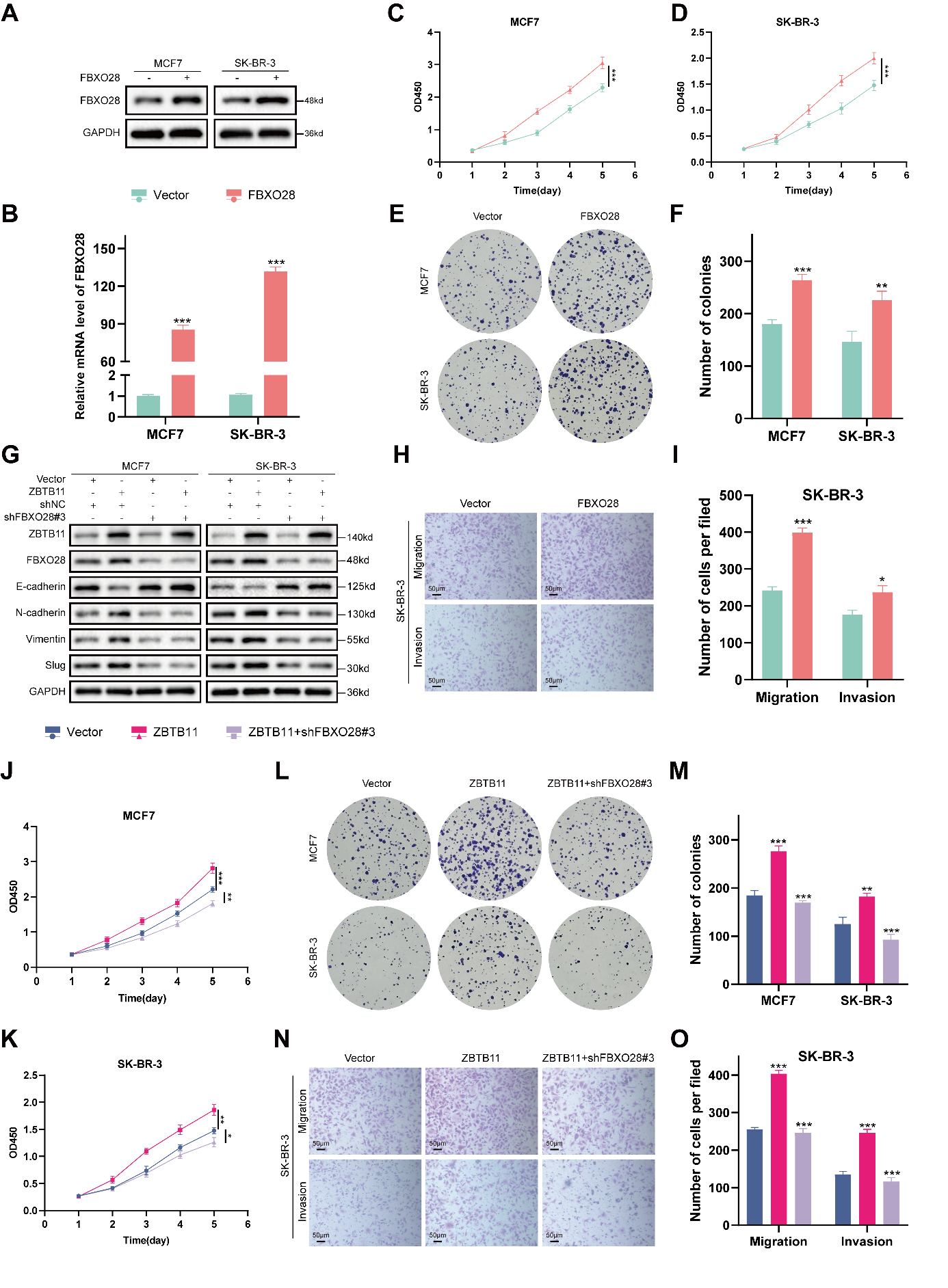


**Supplementary Figure S3.** Functional validation of FBXO28 overexpression and FBXO28-dependent rescue of ZBTB11-induced malignant phenotypes in MCF7 and SK-BR-3 cells.

(A) Western blot validation of FBXO28 overexpression in MCF7 and SK-BR-3 cells. (B) qPCR analysis of FBXO28 mRNA levels in vector and FBXO28-overexpression MCF7 and SK-BR-3 cells. (C, D) CCK-8 proliferation assays of FBXO28-overexpressing MCF7 (C) and SK-BR-3 (D) cells. (E) Representative colony formation images of vector and FBXO28-overexpression MCF7 and SK-BR-3 cells. (F) Quantification of colony formation assays. (G) Western blot analysis of FBXO28 and EMT-related proteins in MCF7 and SK-BR-3 cells expressing vector or ZBTB11 overexpression, with or without FBXO28 knockdown. (H) Representative Transwell migration and invasion images of vector and FBXO28-overexpression SK-BR-3 cells. (I) Quantification of Transwell migration and invasion assays. (J, K) CCK-8 proliferation assays of MCF7 (J) and SK-BR-3 (K) cells expressing vector, ZBTB11-OE, or ZBTB11-OE plus shFBXO28#3. (L) Representative colony formation images of MCF7 and SK-BR-3 cells expressing vector, ZBTB11-OE, or ZBTB11-OE plus shFBXO28#3. (M) Quantification of colony formation assays. (N) Representative Transwell migration and invasion images of SK-BR-3 cells expressing vector, ZBTB11-OE, or ZBTB11-OE plus shFBXO28#3. (O) Quantification of Transwell migration and invasion assays. *P < 0.05, **P < 0.01, ***P < 0.001, ns, not significant.


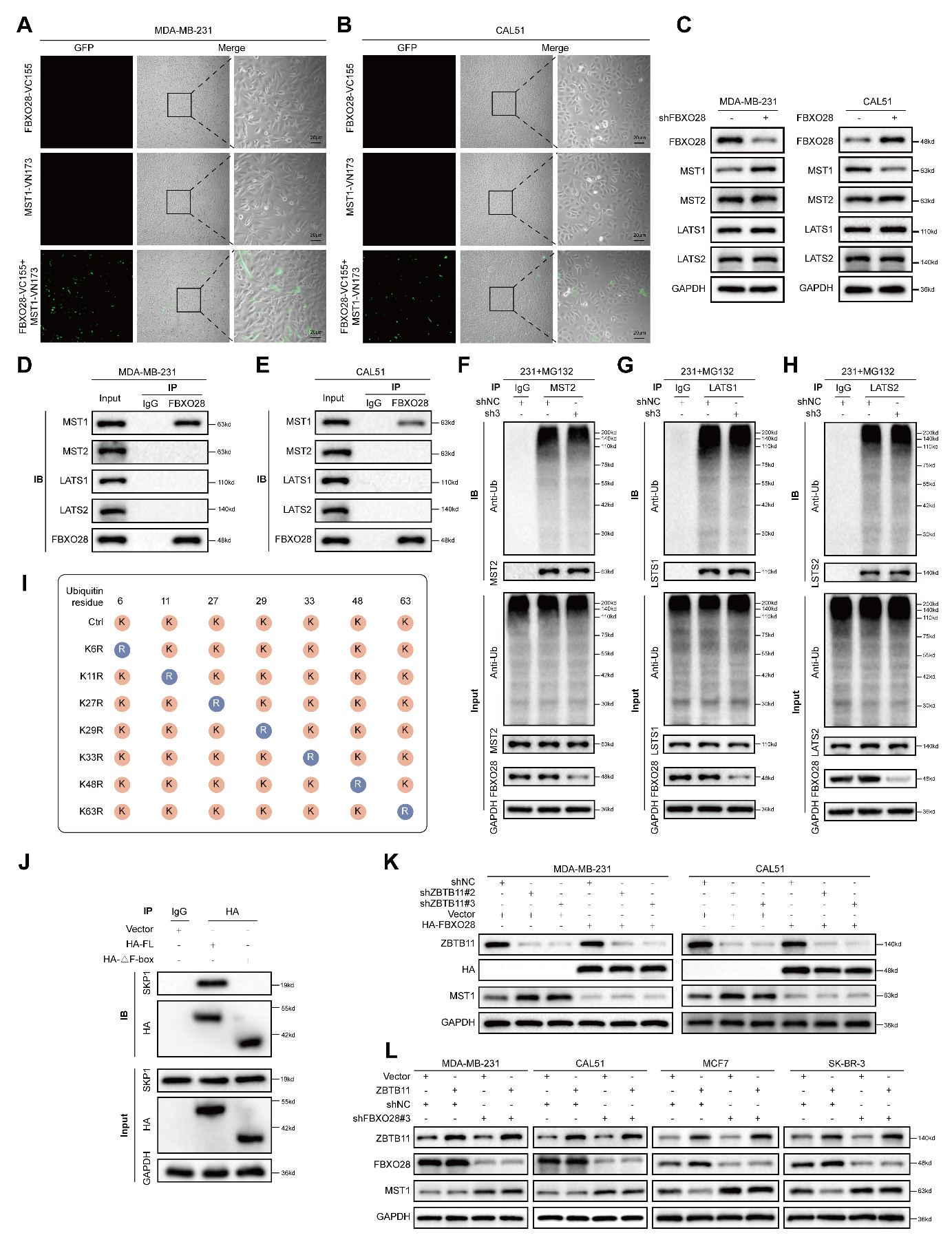


**Supplementary Figure S4**. Specific interaction and regulatory hierarchy among ZBTB11, FBXO28, and MST1.

(A, B) BiFC fluorescence imaging of FBXO28–MST1 interaction in MDA-MB-231 (A) and CAL51 (B) cells. (C) Western blot analysis of MST1, MST2, LATS1, and LATS2 protein levels after FBXO28 knockdown in MDA-MB-231 cells or FBXO28 overexpression in CAL51 cells. (D, E) Co-immunoprecipitation analysis of endogenous FBXO28 with MST1, MST2, LATS1, and LATS2 in MDA-MB-231 (D) and CAL51 (E) cells. (F–H) Ubiquitination assays of MST2 (F), LATS1 (G), and LATS2 (H) in MDA-MB-231 cells with or without FBXO28 knockdown. (I) Schematic of ubiquitin lysine K-to-R mutants used to assess the ubiquitin linkage type of FBXO28-mediated MST1 ubiquitination. (J) Co-immunoprecipitation analysis of FBXO28 interaction with SKP1. (K) Western blot analysis of MST1 expression in ZBTB11-knockdown cells with or without FBXO28 overexpression. (L) Western blot analysis of FBXO28 and MST1 expression in ZBTB11-overexpressing cells with or without FBXO28 knockdown in MDA-MB-231, CAL51, MCF7, and SK-BR-3 cells.

**
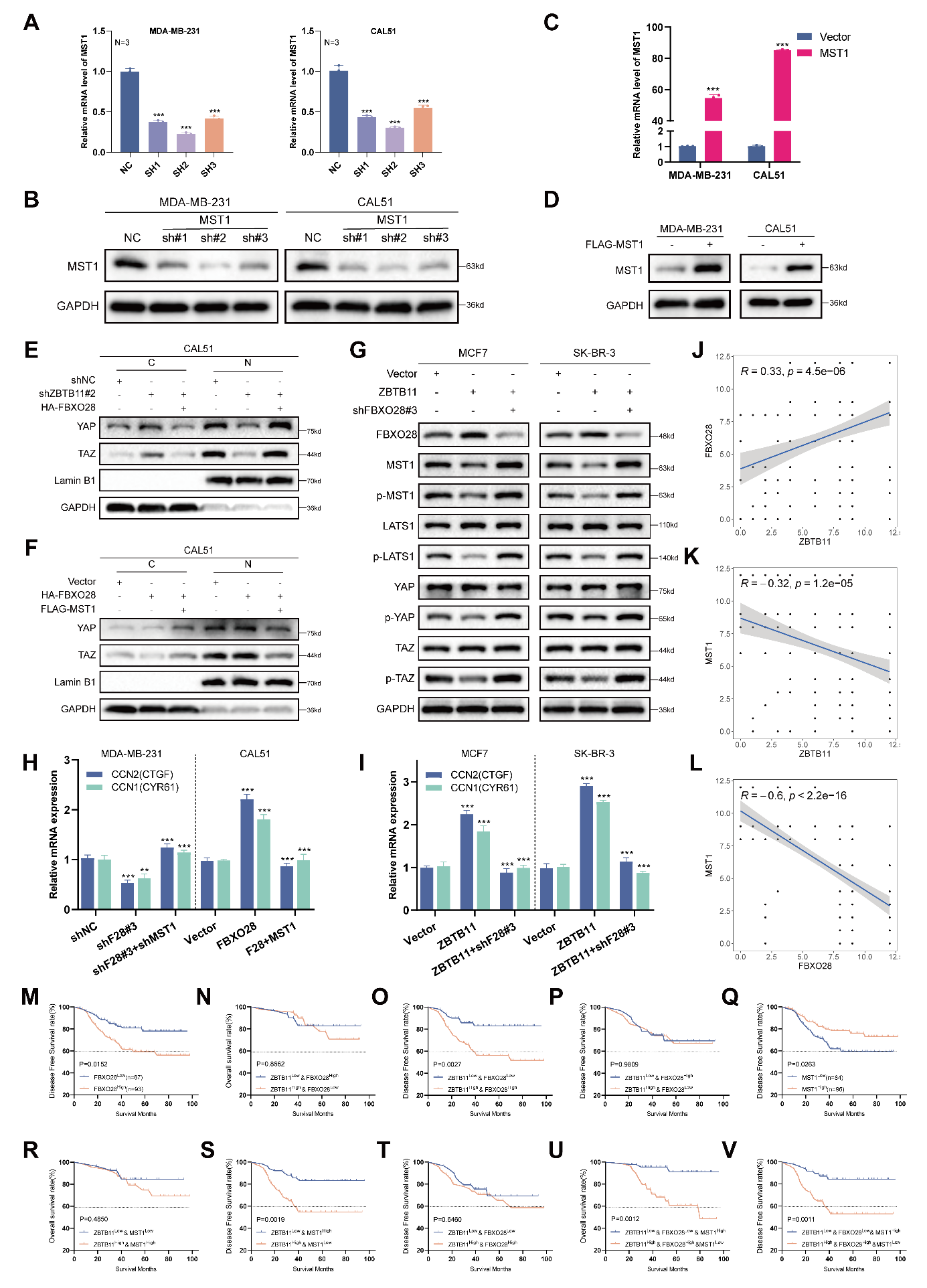
**

**Supplementary Figure S5**. Functional characterization and clinical relevance of MST1, FBXO28, and ZBTB11 expression patterns.

(A) qRT-PCR validation of MST1 knockdown efficiency in MDA-MB-231 and CAL51 cells. (B) Western blot validation of MST1 knockdown efficiency in MDA-MB-231 and CAL51 cells. (C) qRT-PCR validation of MST1 overexpression efficiency in MDA-MB-231 and CAL51 cells. (D) Western blot validation of MST1 overexpression efficiency in MDA-MB-231 and CAL51 cells. (E, F) Nuclear-cytoplasmic fractionation showing YAP/TAZ distribution following ZBTB11 knockdown with or without FBXO28 overexpression (E) or FBXO28 overexpression with or without MST1 co-expression (F). (G) Western blot analysis of Hippo pathway components in MCF7 and SK-BR-3 cells expressing vector, ZBTB11, or ZBTB11 plus shFBXO28#3. (H) qRT-PCR analysis of the representative YAP/TAZ-TEAD target genes CCN2 (CTGF) and CCN1 (CYR61) in MDA-MB-231 cells expressing shNC, shFBXO28#3, or shFBXO28#3 plus siMST1, and in CAL51 cells expressing vector, FBXO28, or FBXO28 plus MST1. (I) qRT-PCR analysis of CCN2 (CTGF) and CCN1 (CYR61) in MCF7 and SK-BR-3 cells expressing vector, ZBTB11, or ZBTB11 plus shFBXO28#3. (J-L) Scatter plots showing correlations between ZBTB11 and FBXO28 (J), ZBTB11 and MST1 (K), and FBXO28 and MST1 (L) based on continuous IRS values in the breast cancer TMA cohort. (M–T) DFS analyses according to FBXO28 expression, MST1 expression, and combined ZBTB11-FBXO28 or ZBTB11-MST1 signatures in the breast cancer TMA cohort. (U, V) OS and DFS analyses of the triple-marker combination ZBTB11-FBXO28-MST1. *P < 0.05, **P < 0.01, ***P < 0.001, ns, not significant.

**Supplementary Tables**

**Supplementary Table S1.** Comparison of baseline clinical characteristics based on ZBTB11 expression level.

| **Variables** |  | | **n** | **ZBTB11** | | **χ2 value** | **P value** |
| --- | --- | --- | --- | --- | --- | --- | --- |
|  |  |  |  | **High(%)** | **Low(%)** |  |  |
| Age | |  |  |  |  | 0.200 | 0.655 |
|  | ＜55 | | 110 | 65(62.5) | 45(59.21) |  |  |
|  | ≥55 | | 70 | 39(37.5) | 31(40.79) |  |  |
| Tumor diameter |  | |  |  |  | 7.940 | 0.005 |
|  | ＜2cm | | 49 | 20(19.23) | 29(38.16) |  |  |
|  | ≥2cm | | 131 | 84(80.77) | 47(61.84) |  |  |
| Grade |  | |  |  |  | 3.581 | 0.167 |
|  | Ⅰ | | 35 | 23(22.12) | 12(15.79) |  |  |
|  | Ⅱ | | 71 | 35(33.65) | 36(47.37) |  |  |
|  | Ⅲ | | 74 | 46(44.23) | 28(36.84) |  |  |
| Stage |  | |  |  |  | 7.811 | 0.020 |
|  | Ⅰ | | 30 | 12(11.54) | 18(23.68) |  |  |
|  | Ⅱ | | 98 | 55(52.88) | 43(56.58) |  |  |
|  | Ⅲ | | 52 | 37(35.58) | 15(19.74) |  |  |
| Molecular subtype |  | |  |  |  | 0.722 | 0.868 |
|  | Luminal A | | 38 | 21(20.19) | 17(22.37) |  |  |
|  | Luminal B | | 74 | 45(43.27) | 29(38.16) |  |  |
|  | Basal-like | | 43 | 25(24.04) | 18(23.68) |  |  |
|  | HER -positive+ | | 25 | 13(12.5) | 12(15.79) |  |  |
| ER Status |  | |  |  |  | 0.100 | 0.752 |
|  | Negative | | 71 | 40(38.46) | 31(40.79) |  |  |
|  | Positive | | 109 | 64(61.54) | 45(59.21) |  |  |
| PR Status |  | |  |  |  | 0.184 | 0.668 |
|  | Negative | | 91 | 54(51.92) | 37(48.68) |  |  |
|  | Positive | | 89 | 50(48.08) | 39(51.32) |  |  |
| HER2 status |  | |  |  |  | 0.339 | 0.560 |
|  | Negative | | 125 | 74(71.15) | 51(67.11) |  |  |
|  | Positive | | 55 | 30(28.85) | 25(32.89) |  |  |
| Ki-67 index |  | |  |  |  | 7.174 | 0.007 |
|  | ≤14 | | 52 | 22(21.15) | 30(39.47) |  |  |
|  | ＞14 | | 128 | 82(78.85) | 46(60.53) |  |  |

**Supplementary Table S2.** Cox proportional hazards regression analysis of OS and DFS in the TMA cohort

| **Endpoint** | **Variable** | **Comparison** | **Univariate HR** | **95% CI** | **P value** | **Multivariate HR** | **95% CI** | **P value** |
| --- | --- | --- | --- | --- | --- | --- | --- | --- |
| OS | ZBTB11 expression | High vs low | 1.678 | 1.436–1.959 | <0.001 | 1.251 | 1.050–1.491 | 0.012 |
| OS | Tumor diameter | ≥2 cm vs <2 cm | 2.127 | 1.741–2.600 | <0.001 | 1.578 | 1.220–2.041 | <0.001 |
| OS | Histological grade | Per grade increase | 3.486 | 1.884–6.454 | <0.001 | 2.308 | 1.217–4.378 | 0.01 |
| OS | TNM stage | Per stage increase | 4.724 | 2.543–8.778 | <0.001 | 2.722 | 1.244–5.955 | 0.012 |
| OS | ER status | Positive vs negative | 0.452 | 0.236–0.867 | 0.017 | 0.735 | 0.362–1.494 | 0.395 |
| OS | Ki-67 index | High vs low | 1.023 | 1.011–1.036 | <0.001 | 1.018 | 1.003–1.033 | 0.021 |
| OS | Age | ≥55 vs <55 years | 1.003 | 0.973–1.033 | 0.868 | - | - | NI |
| OS | PR status | Positive vs negative | 0.674 | 0.350–1.301 | 0.24 | - | - | NI |
| OS | HER2 status | Positive vs negative | 1.108 | 0.556–2.208 | 0.77 | - | - | NI |
| DFS | ZBTB11 expression | High vs low | 1.607 | 1.394–1.853 | <0.001 | 1.377 | 1.185–1.600 | <0.001 |
| DFS | Tumor diameter | ≥2 cm vs <2 cm | 1.833 | 1.528–2.200 | <0.001 | 1.374 | 1.110–1.699 | 0.003 |
| DFS | Histological grade | Per grade increase | 1.807 | 1.212–2.695 | 0.004 | 1.344 | 0.912–1.980 | 0.135 |
| DFS | TNM stage | Per stage increase | 2.677 | 1.717–4.174 | <0.001 | 1.666 | 0.984–2.821 | 0.057 |
| DFS | ER status | Positive vs negative | 1.018 | 1.008–1.028 | <0.001 | 1.013 | 1.002–1.024 | 0.017 |
| DFS | Ki-67 index | High vs low | 1.005 | 0.981–1.030 | 0.668 | - | - | NI |
| DFS | Age | ≥55 vs <55 years | 0.482 | 0.282–0.823 | 0.008 | - | - | NI |
| DFS | PR status | Positive vs negative | 0.61 | 0.353–1.055 | 0.077 | - | - | NI |
| DFS | HER2 status | Positive vs negative | 1.563 | 0.904–2.702 | 0.11 | - | - | NI |

**Supplementary Table S3.** Interobserver agreement for IHC high/low classification of ZBTB11, FBXO28, and MST1 in breast cancer tissues.

| **Marker** | **High/Low Agreement (%)** | **Cohen’s kappa** |
| --- | --- | --- |
| ZBTB11 | 92.2% | 0.841 |
| FBXO28 | 93.3% | 0.867 |
| MST1 | 90.6% | 0.811 |

High/Low agreement (%) represents the percentage of samples for which two independent pathologists assigned the same high/low classification. Cohen’s kappa indicates interobserver agreement after accounting for chance. High expression was defined as IRS ≥ 7, low expression as IRS < 7.

Supplementary Table S4. **List of antibodies used in this study**

| **Name** | **Catalog** | **Application** |
| --- | --- | --- |
| ZBTB11 | Sigma-Aldrich: HPA015328 | WB, IF, IHC |
| FBXO28 | Proteintech | WB, IHC |
| FBXO28 | Santa Cruz: sc-376851 | IF, CO-IP |
| MST1 | Proteintech: 22245-1-AP | WB, IHC, IF, CO-IP |
| E-cadherin | Proteintech: 20874-1-AP | WB, IF, IHC |
| N-cadherin | Proteintech: 22018-1-AP | WB |
| Vimentin | Proteintech: 10366-1-AP | WB, IF, IHC |
| Slug | Proteintech: 12129-1-AP | WB, IF |
| p-MST1(Ser183) | Immunoway: YP0695 | WB |
| MST2 | Proteintech: 12097-1-AP | WB, CO-IP |
| LATS1 | Affinity: AF7669 | WB, CO-IP |
| LATS2 | Proteintech: 20276-1-AP | WB, CO-IP |
| p-LATS1(Ser909) | Biodragon: BD-PP1382 | WB |
| YAP | Affinity: DF3182 | WB, IHC |
| p-YAP(Ser127) | Proteintech: 80694-2-RR | WB |
| TAZ | Affinity: DF4653 | WB |
| p-TAZ(Ser89) | Biodragon: BD-PP1526 | WB |
| FLAG | Proteintech: 66008-4-Ig | WB, CO-IP, ChIP |
| HA | Proteintech: 51064-2-AP | WB, CO-IP |
| GST | Proteintech: 66001-2-Ig | WB |
| Ubiquitin | Santa Cruz: sc-8017 | WB |
| Lamin B1 | Proteintech: 66095-1-Ig | WB |
| GAPDH | Proteintech: 60004-1-Ig | WB |
| Goat Anti-Mouse | Proteintech: SA00001-1 | WB |
| Goat Anti-Rabbit | Proteintech: SA00001-2 | WB |

**Supplementary Table S5.** shRNA sequences used in this study

| **Name** | **Sequence** |
| --- | --- |
| shZBTB11#1 | GCTCGAACACTCCGTAAACAT |
| shZBTB11#2 | GGTGTAAAGCCACATGCATGC |
| shZBTB11#3 | GCACATGAACAAACATCTTGG |
| shFBXO28#1 | GAGAATGTTGAATCAGGGATT |
| shFBXO28#2 | GAGAGTCAGAAAGGAGAAACC |
| shFBXO28#3 | GTTATGTCCTACGACGAAATT |
| shMST1#1 | CCAGAGCTATGGTCAGATAAC |
| shMST1#2 | GCCCTCATGTAGTCAAATATT |
| shMST1#3 | CCGGCCAGATTGTTGCTATTA |

**Supplementary Table S6.** Primer sequences used in this study

| **Name** | **Primer Sequence** | **Application** |
| --- | --- | --- |
| ZBTB11 | F: GGGCACCGAGGGCAATGTC | RT–qPCR |
|  | R: CCGCCGCTGGTAATACAGAGTC |  |
| FBXO28 | F: TGCTGCTGTTGAAACAAGGC | RT–qPCR |
|  | R: GCAGACGGTCCTGGAACTTT |  |
| MST1 | F: TCACAAGTACACGCCCACTC | RT–qPCR |
|  | R: GATTTGATGCCGCAGCTCTG |  |
| GAPDH | F: CACCCACTCCTCCACCTTTGAC | RT–qPCR |
|  | R: GTCCACCACCCTGTTGCTGTAG |  |
| MTPN | F: GAGTTCATGTGGGCCCTGAA | RT–qPCR |
|  | R: TGCCCACAATCTGCTGCATA |  |
| DDX21 | F: TGCTCAGTCCTTGCATGGAG | RT–qPCR |
|  | R: GGATGTCTAACCCACGTGCA |  |
| FAM91A1 | F: CAAAGCAAACCTCTGGTGCC | RT–qPCR |
|  | R: TTTCTGCAAAGGCCATGTGC |  |
| TIMM23 | F: AGGATGTTGCATGACAGGGG | RT–qPCR |
|  | R: AAAGTGCCCCTTGCCTAGTC |  |
| ZMPSTE24 | F: ACCCCGCAATGAGGAAGAAG | RT–qPCR |
|  | R: CTTCCAGTGCCCCAGTTCAT |  |
| WDR36 | F: CTCCTGATTGTGGTGGGTCC | RT–qPCR |
|  | R: ATCACGCTTTCTGTCCAGCA |  |
| ARPP19 | F: CCCTTGGTCTCTGCAACCAT | RT–qPCR |
|  | R: AACCTCCAGGCTTTTGTCCC |  |
| GSPT1 | F: TCTGCCCAGGCTATAATGCG | RT–qPCR |
|  | R: GTTTGACAAAACGGGGTCGG |  |
| NOC3L | F: ACCCAAAGGAAAAGCGACCA | RT–qPCR |
|  | R: TTCGCATGAACAGGCTCACT |  |
| ECHDC1 | F: TTCAAGGTTGGGCATTGGGT | RT–qPCR |
|  | R: GCCCATCTCTTTGTGGACGA |  |
| LSG1 | F: ACACCTGGTCACACAAAGCA | RT–qPCR |
|  | R: CATCACCAAGCCAGGACAGT |  |
| CSNK1G3 | F: CAAATGCACCCATCACAGCC | RT–qPCR |
|  | R: TGGCGCTGTATGGTTTTCCT |  |
| TAF13 | F: ATGGCTTTGGGGATGACCAG | RT–qPCR |
|  | R: GGCAAACTTCCTTGGGTCCT |  |
| RIOK1 | F: AATCCAAGGCTCGGGAGTTG | RT–qPCR |
|  | R: CACGGACTGAGACACGTCAA |  |
| RSL1D1 | F: GAAGGAACAGACCCCAGAGC | RT–qPCR |
|  | R: CCAGCTGTGGGATTTCGTCT |  |
| ALYREF | F: GGCCAAAACAACTTCCCGAC | RT–qPCR |
|  | R: CCAGATTGGACACCAGCAGT |  |
| POMP | F: GGAATTCAAGGCAGTGCAGC | RT–qPCR |
|  | R: CCACCATCAAGTGTGGCTCT |  |
| TSC2 | F: GCTGAACATCATCGAACGGC | RT–qPCR |
|  | R: CGTGGAACTCGTTCTGGTCA |  |
| PPIL2 | F: GCGACCTGACACCAAAAACC | RT–qPCR |
|  | R: TCGTCTTTGAAGGGCTTCCC |  |
| TMA16 | F: TGAAAAGGCCTTGCGTCTCA | RT–qPCR |
|  | R: AATCTGCTCCAGCTCACTGC |  |
| FBXW11 | F: TGGTACGCACTGATCCCCTA | RT–qPCR |
|  | R: ATTTGGAGGGCCATCTGTGG |  |
| PNPLA6 | F: TCCATCGGGTCTTCCAGGAT | RT–qPCR |
|  | R: CATGGCTGAGGCGGTGATAT |  |
| ZDHHC7 | F: GTGGTCAACGGGGTCATCTT | RT–qPCR |
|  | R: CACCACAAATGCCCACAAGG |  |
| TXN2 | F: AGAGAAGATGGTGGCCAAGC | RT–qPCR |
|  | R: CACCACGTCCCCATTCTTCA |  |
| SEC14L1 | F: GGCCTATCAGCTCATGGACC | RT–qPCR |
|  | R: GTGTCTCAGGGTAGTTGGCC |  |
| NAT14 | F: GCGGGAGATGAGGGAAGATG | RT–qPCR |
|  | R: GAAGGAAGCCAGGACAAAGC |  |
| MAGED1 | F: GCCATCCAGATCTCAGAGGC | RT–qPCR |
|  | R: ATCTCATTGGCAGTTGGGGG |  |
| MAF1 | F: GCCTTAGCTGGGTGGTGAAT | RT–qPCR |
|  | R: ATGTCACATTCAGCCAGGCA |  |
| GANAB | F: CCCAATGGCCTTGTATGGGT | RT–qPCR |
|  | R: CCAGGTCTCTGCAGCATTGA |  |
| MAGEF1 | F: AACAGTTTGACCGCAAGCAC | RT–qPCR |
|  | R: TCCCTGGCGCTATTACCTCT |  |
| RPS14 | F: CGTGTGACTGGTGGGATGAA | RT–qPCR |
|  | R: GGCCCGGAGTTTGATGTGTA |  |
| CCN2 | F: GTTACCAATGACAACGCCTCCTG | RT–qPCR |
|  | R: TTGCCCTTCTTAATGTTCTCTTCCAG |  |
| CCN1 | F: TGGAGCCTCGCATCCTATACAAC | RT–qPCR |
|  | R: GTCATTGGTAACTCGTGTGGAGATAC |  |
| DDOST | F: TGCGGGATTGAGTTTGACGA | RT–qPCR |
|  | R: AGCAGGTTCTCAGTGTCAGC |  |
| FBXO28 | F: TGAGTCTCTCTCCGCCTGTT | ChIP–qPCR |
|  | R: AAGGGGAACAGGGACACTTC |  |

**Supplementary Date Files**

**Supplementary Data S1.** Differentially expressed genes between primary and metastatic breast cancer tissues.

(Provided as a separate Excel file)

**Supplementary Data S2.** Differentially expressed genes identified upon ZBTB11 knockdown in MDA-MB-231 cells.

(Provided as a separate Excel file)

**Supplementary Data S3.** Predicted ZBTB11 target genes integrated from four independent databases (FIMO_JASPAR, PWMEnrich_JASPAR, GTRD, and ChIP-Atlas)

(Provided as a separate Excel file)

**Supplementary Data S4.** Candidate FBXO28-interacting proteins identified by HA-FBXO28 immunoprecipitation and LC-MS/MS analysis in MDA-MB-231 cells.

(Provided as a separate Excel file)

**Supplementary Data S5.** IHC IRS scores and high/low classification for ZBTB11, FBXO28, and MST1 in 180 breast cancer samples.

(Provided as a separate Excel file)

**Supplementary Data S6.** Available original Western blot source images corresponding to immunoblots presented in the main and supplementary figures, including target-protein strip/region source images retained from the original experiments. Target proteins, cell lines, experimental groups, expected molecular weights, molecular weight marker information, and loading controls are annotated where applicable.

(Provided as a separate PDF file)
