# Supplementary material for: ZBTB11 Promotes Breast Cancer Progression by Activating FBXO28‐Mediated MST1 Degradation and Suppressing Hippo Signaling
Source: Adv Sci (Weinh). 2026 Jul 20:e76618. Online ahead of print. doi: 10.1002/advs.76618 (PMC13383149; doi:10.1002/advs.76618)

Figure 1

Available original Western blot source images

I

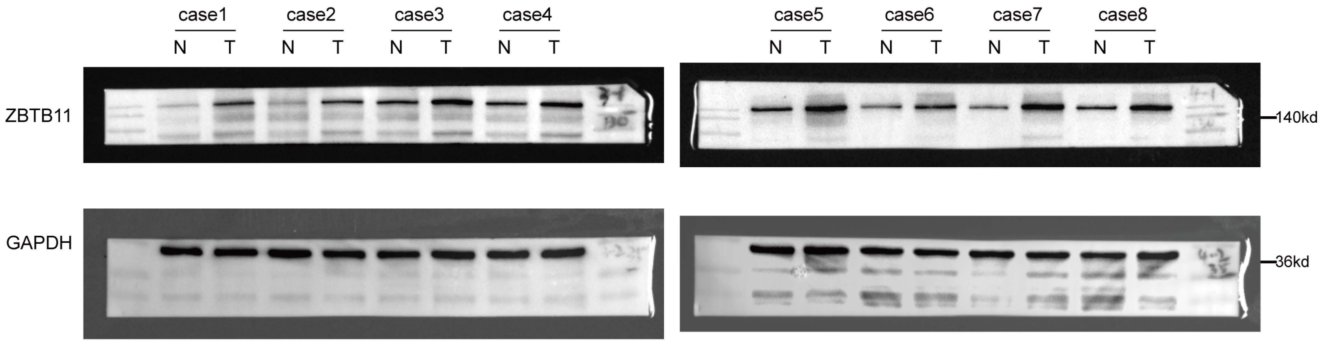

Figure 3

Available original Western blot source images

A B

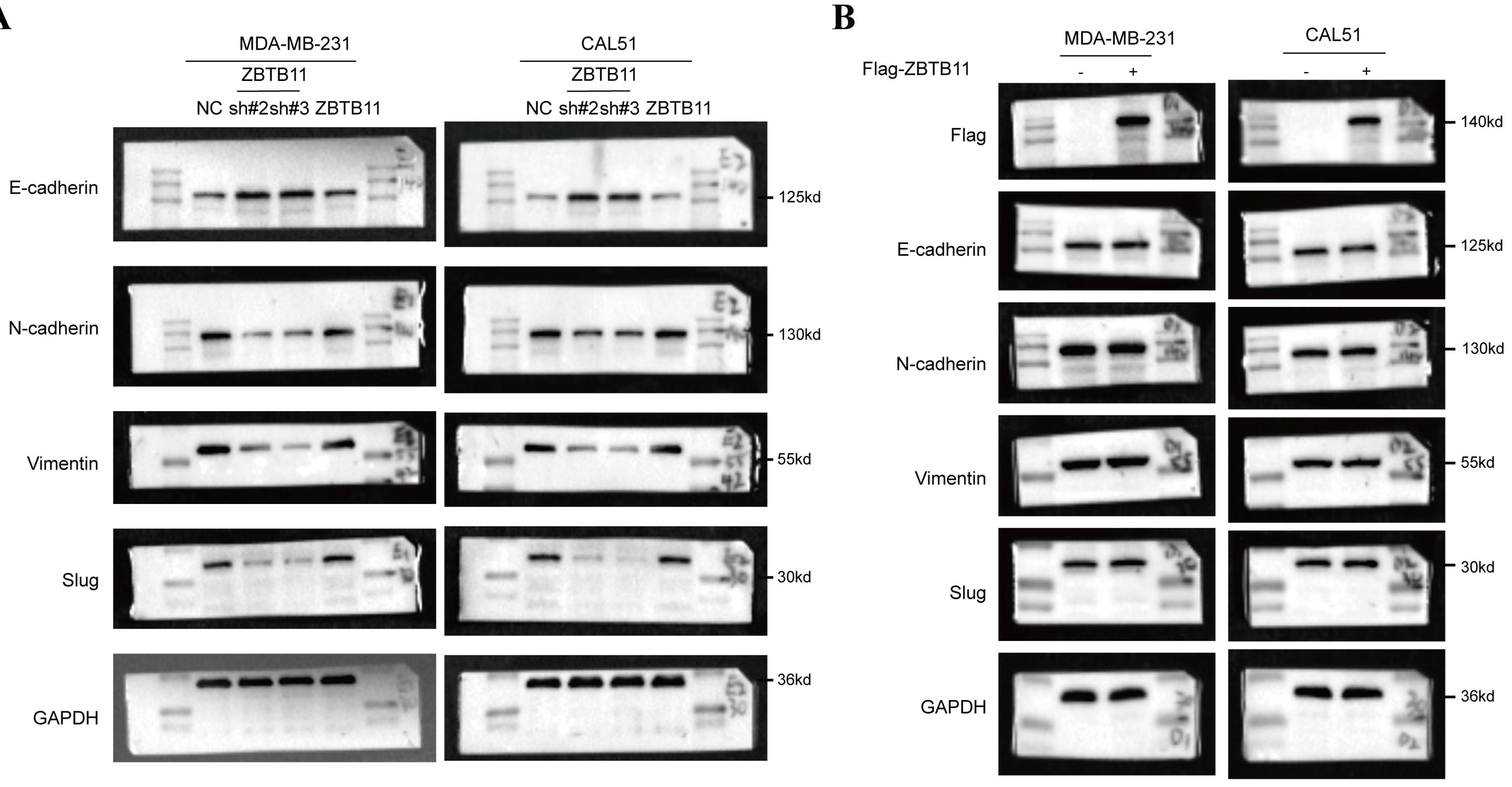

Figure 4

K

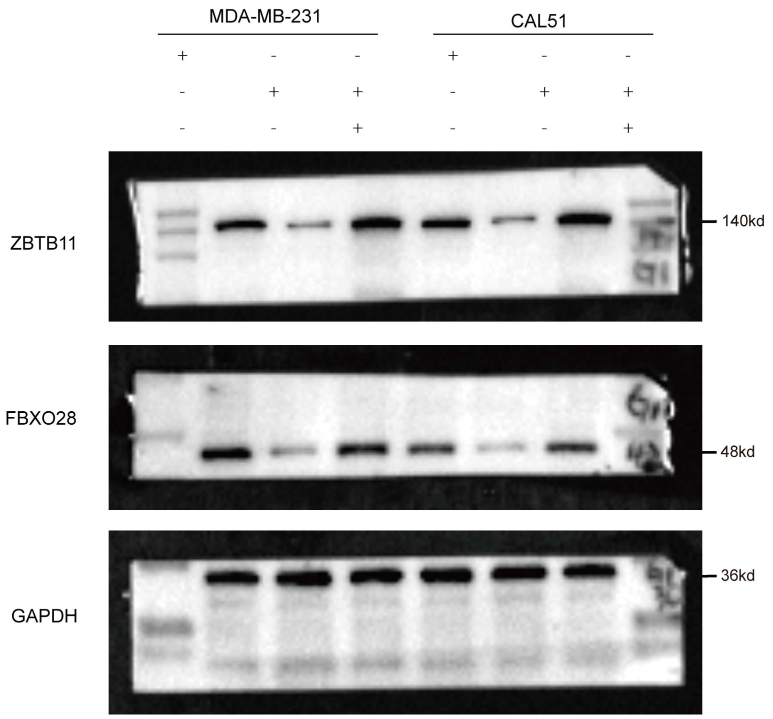
MDA-MB-231
CAL51
+ - - + - -
- + + - + +
- - + - - +
ZBTB11
140kd
FBXO28
48kd
GAPDH
36kd

N

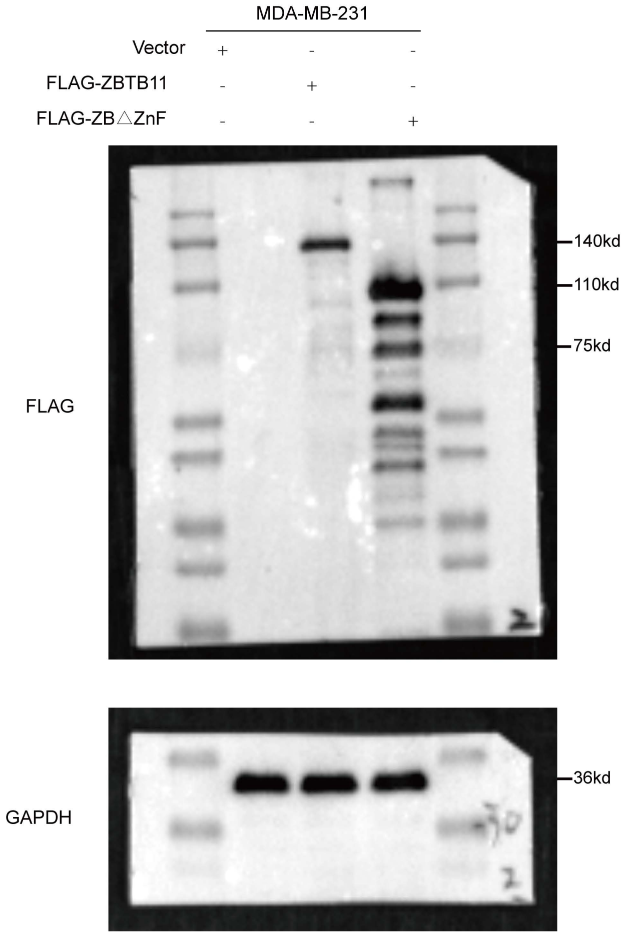
MDA-MB-231
Vector + - -
FLAG-ZBTB11 - + -
FLAG-ZB△ZnF - - +
FLAG
140kd
110kd
75kd
GAPDH
36kd

Figure 5

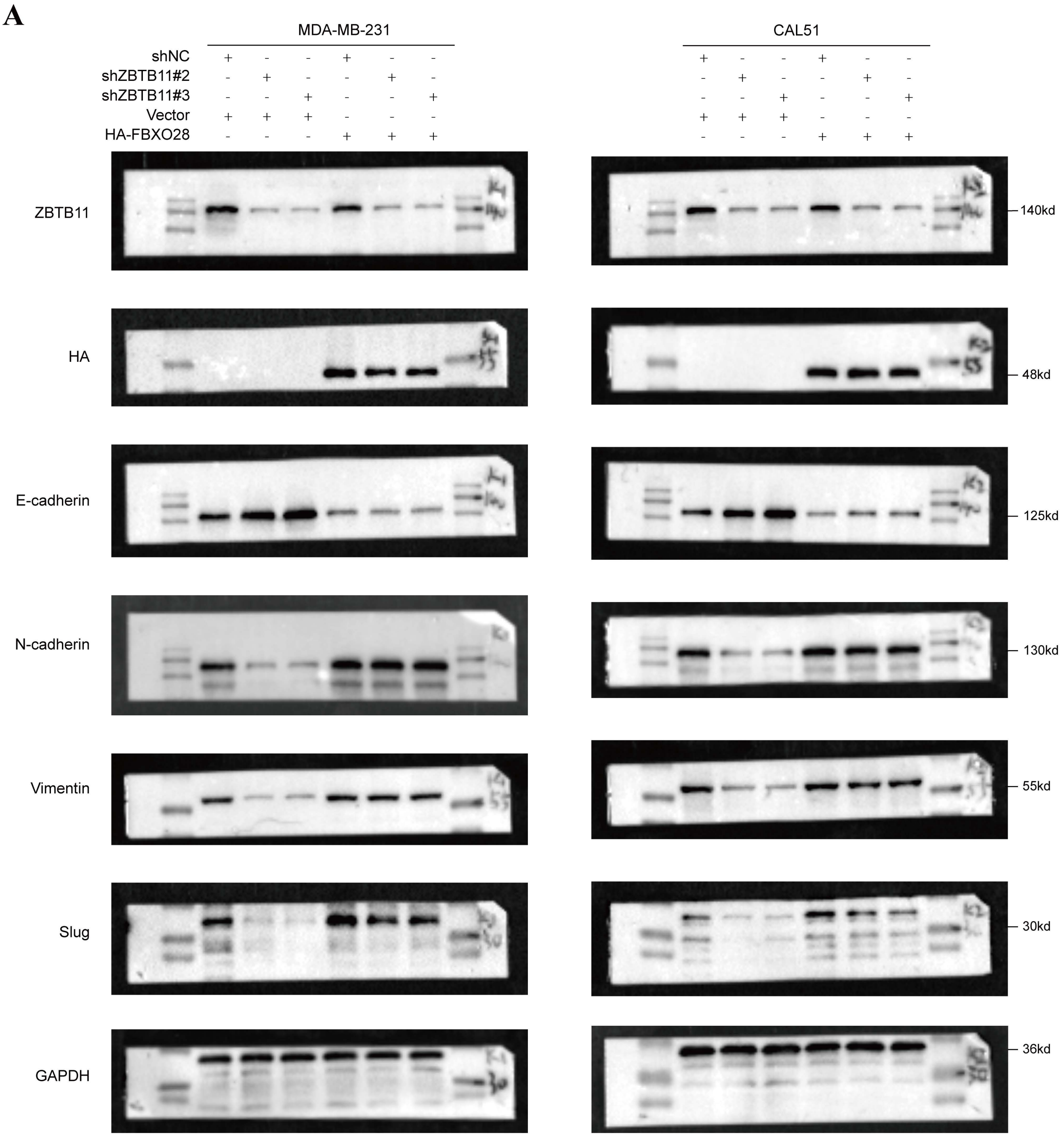
A
MDA-MB-231
CAL51
shNC
shZBTB11#2
shZBTB11#3
Vector
HA-FBXO28
ZBTB11
HA
E-cadherin
N-cadherin
Vimentin
Slug
GAPDH
140kd
48kd
125kd
130kd
55kd
30kd
36kd

Figure 6

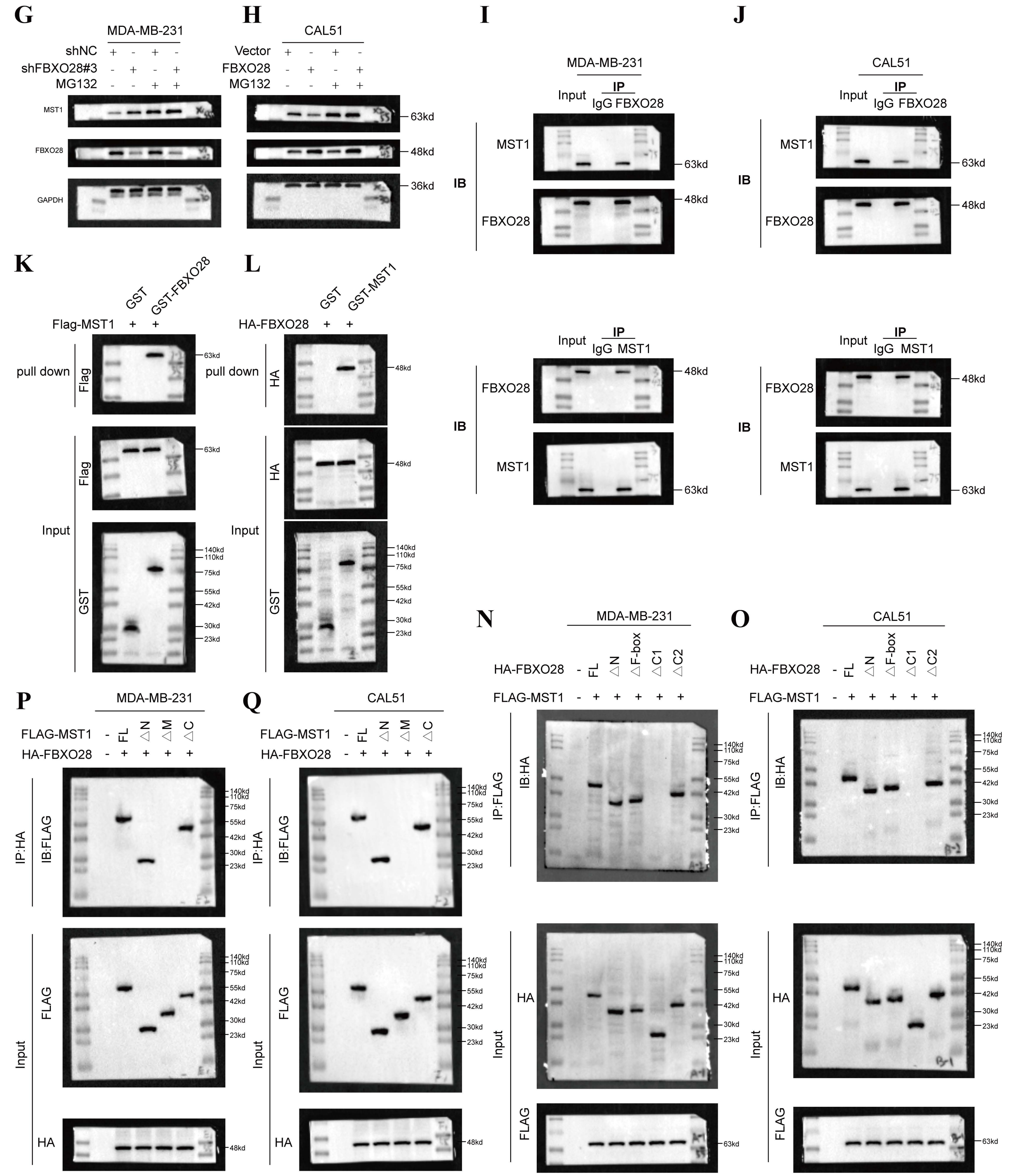
G
MDA-MB-231
shNC + - + -
shFBXO28#3 - + - +
MG132 - - + +
MST1
FBXO28
GAPDH
H
CAL51
Vector + - + -
FBXO28 - + - +
MG132 - - + +
63kd
48kd
36kd
I
MDA-MB-231
Input
IP
IgG FBXO28
IB
MST1
FBXO28
63kd
48kd
Input
IP
IgG MST1
FBXO28
MST1
48kd
63kd
J
CAL51
Input
IP
IgG FBXO28
IB
MST1
FBXO28
63kd
48kd
Input
IP
IgG MST1
FBXO28
MST1
48kd
63kd
K
GST
GST-FBXO28
Flag-MST1 + +
pull down
Flag
Input
GST
63kd
140kd
110kd
75kd
55kd
42kd
30kd
23kd
L
GST
GST-MST1
HA-FBXO28 + +
pull down
HA
Input
GST
48kd
140kd
110kd
75kd
55kd
42kd
30kd
23kd
N
MDA-MB-231
HA-FBXO28 - FL △N △F-box △C1 △C2
FLAG-MST1 - + + + + +
IP:FLAG
IB:HA
Input
HA
FLAG
140kd
110kd
75kd
55kd
42kd
30kd
23kd
63kd
O
CAL51
HA-FBXO28 - FL △N △F-box △C1 △C2
FLAG-MST1 - + + + + +
IP:FLAG
IB:HA
Input
HA
FLAG
140kd
110kd
75kd
55kd
42kd
30kd
23kd
63kd
P
MDA-MB-231
FLAG-MST1 - FL △N △M △C
HA-FBXO28 - + + + +
IP:HA
IB:FLAG
Input
FLAG
HA
140kd
110kd
75kd
55kd
42kd
30kd
23kd
48kd
Q
CAL51
FLAG-MST1 - FL △N △M △C
HA-FBXO28 - + + + +
IP:HA
IB:FLAG
Input
FLAG
HA
140kd
110kd
75kd
55kd
42kd
30kd
23kd
48kd

Figure 7

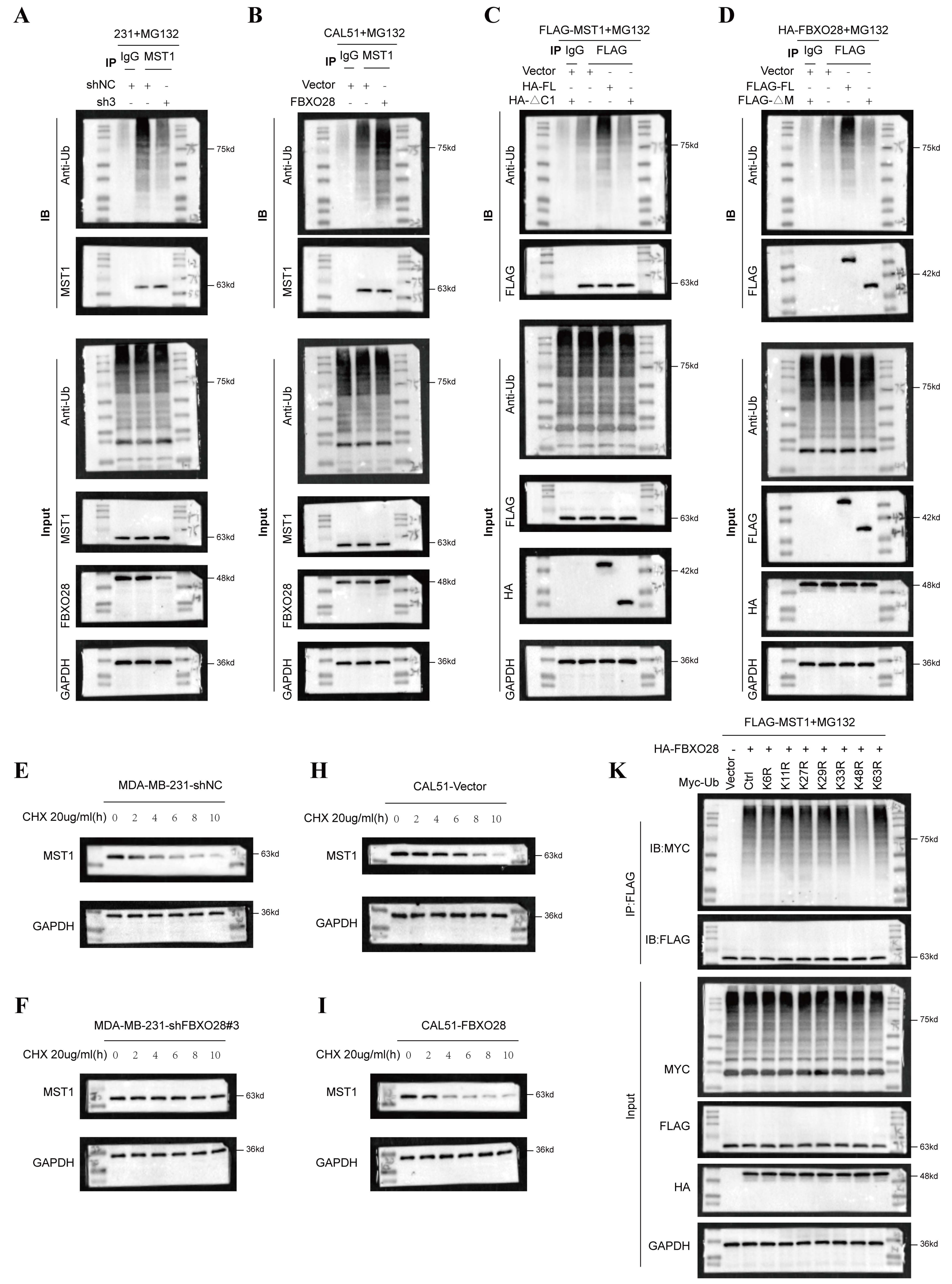

A
231+MG132
IP IgG MST1
shNC + + -
sh3 - - +
IB
Anti-Ub
MST1
Input
Anti-Ub
MST1
FBXO28
GAPDH
75kd
63kd
48kd
36kd
B
CAL51+MG132
IP IgG MST1
Vector + + -
FBXO28 - - +
IB
Anti-Ub
MST1
Input
Anti-Ub
MST1
FBXO28
GAPDH
75kd
63kd
48kd
36kd
C
FLAG-MST1+MG132
IP IgG FLAG
Vector + + - -
HA-FL - - + -
HA-△C1 + - - +
IB
Anti-Ub
FLAG
Input
Anti-Ub
FLAG
HA
GAPDH
75kd
63kd
42kd
36kd
D
HA-FBXO28+MG132
IP IgG FLAG
Vector + + - -
FLAG-FL - - + -
FLAG-△M + - - +
IB
Anti-Ub
FLAG
Input
Anti-Ub
FLAG
HA
GAPDH
75kd
42kd
48kd
36kd
E
MDA-MB-231-shNC
CHX 20ug/ml(h) 0 2 4 6 8 10
MST1
GAPDH
63kd
36kd
H
CAL51-Vector
CHX 20ug/ml(h) 0 2 4 6 8 10
MST1
GAPDH
63kd
36kd
F
MDA-MB-231-shFBXO28#3
CHX 20ug/ml(h) 0 2 4 6 8 10
MST1
GAPDH
63kd
36kd
I
CAL51-FBXO28
CHX 20ug/ml(h) 0 2 4 6 8 10
MST1
GAPDH
63kd
36kd
K
FLAG-MST1+MG132
HA-FBXO28 - + + + + + + + +
Myc-Ub Vector Ctrl K6R K11R K27R K29R K33R K48R K63R
IP:FLAG
IB:MYC
IB:FLAG
Input
MYC
FLAG
HA
GAPDH
75kd
63kd
48kd
36kd

Figure 8

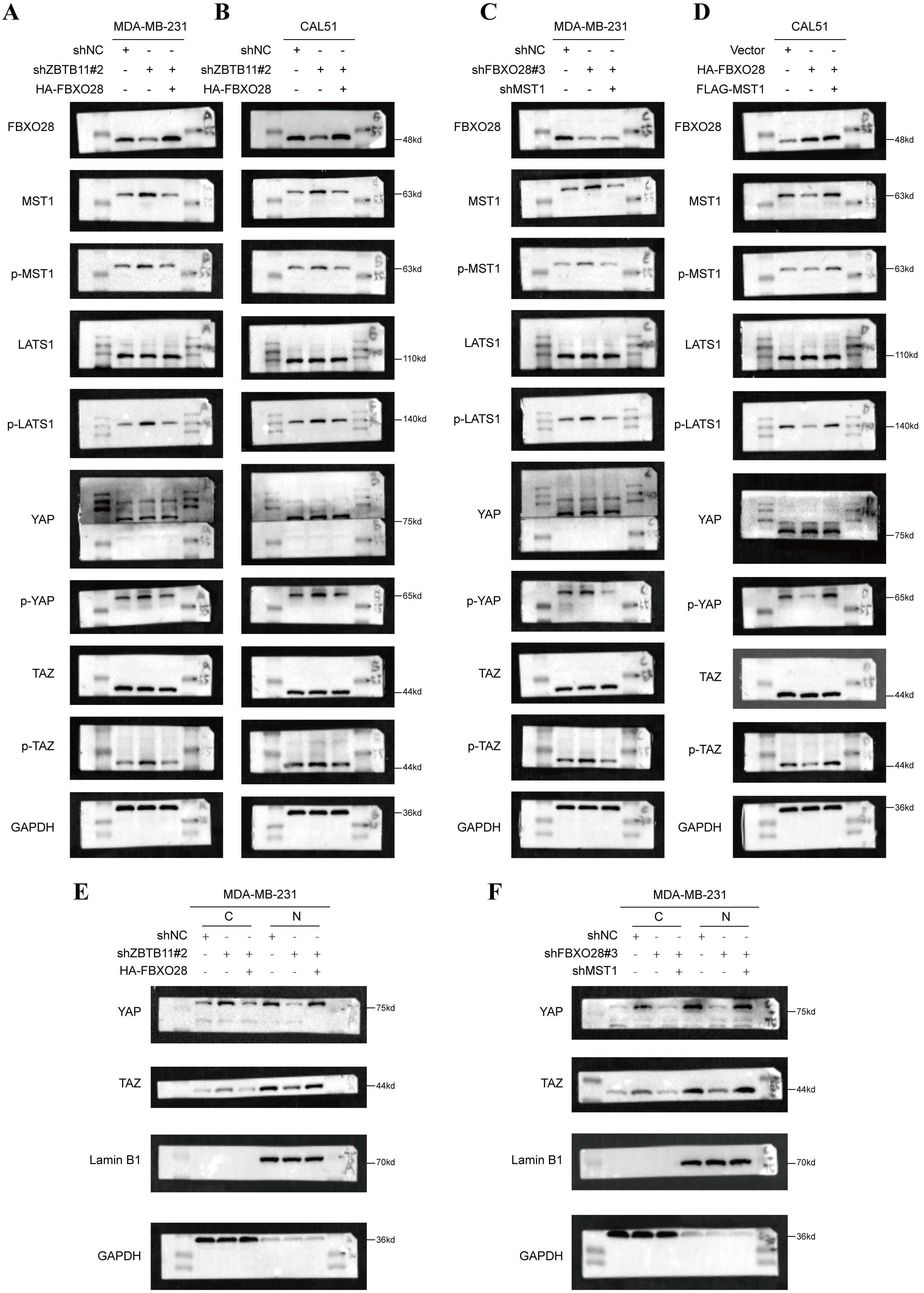

A
MDA-MB-231
shNC + - -
shZBTB11#2 - + +
HA-FBXO28 - - +
FBXO28
MST1
p-MST1
LATS1
p-LATS1
YAP
p-YAP
TAZ
p-TAZ
GAPDH
B
CAL51
shNC + - -
shZBTB11#2 - + +
HA-FBXO28 - - +
48kd
63kd
63kd
110kd
140kd
75kd
65kd
44kd
44kd
36kd
C
MDA-MB-231
shNC + - -
shFBXO28#3 - + +
shMST1 - - +
FBXO28
MST1
p-MST1
LATS1
p-LATS1
YAP
p-YAP
TAZ
p-TAZ
GAPDH
D
CAL51
Vector + - -
HA-FBXO28 - + +
FLAG-MST1 - - +
FBXO28
MST1
p-MST1
LATS1
p-LATS1
YAP
p-YAP
TAZ
p-TAZ
GAPDH
48kd
63kd
63kd
110kd
140kd
75kd
65kd
44kd
44kd
36kd
E
MDA-MB-231
C
N
shNC + - - + - -
shZBTB11#2 - + + - + +
HA-FBXO28 - - + - - +
YAP
TAZ
Lamin B1
GAPDH
75kd
44kd
70kd
36kd
F
MDA-MB-231
C
N
shNC + - - + - -
shFBXO28#3 - + + - + +
shMST1 - - + - - +
YAP
TAZ
Lamin B1
GAPDH
75kd
44kd
70kd
36kd

Supplementary Figure S1

B

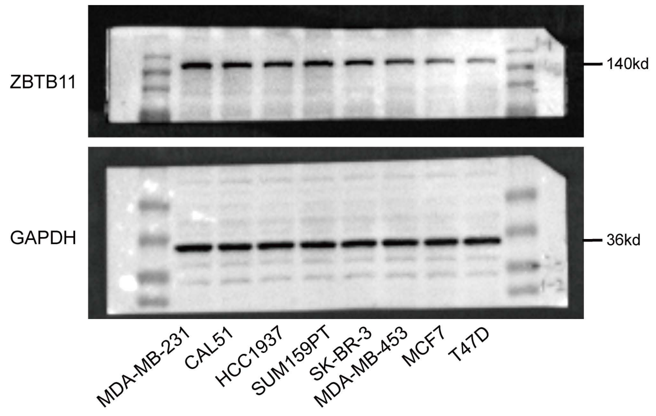

E

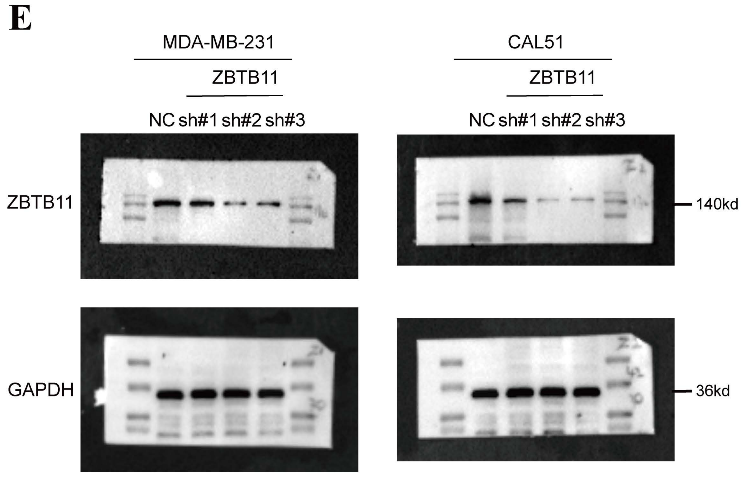

H

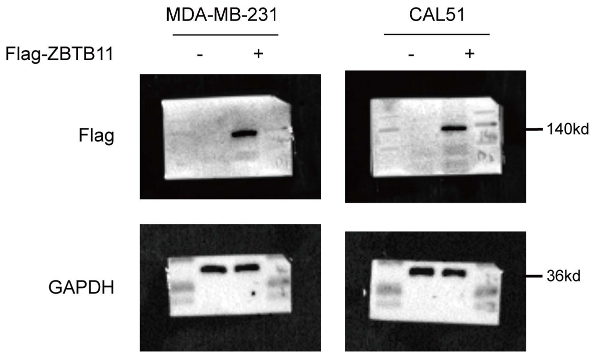

P

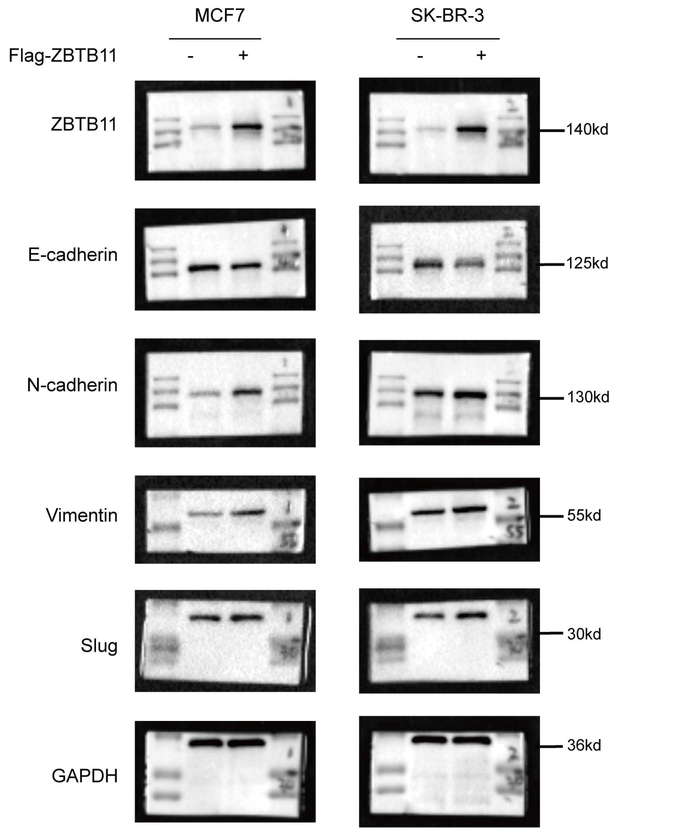

I

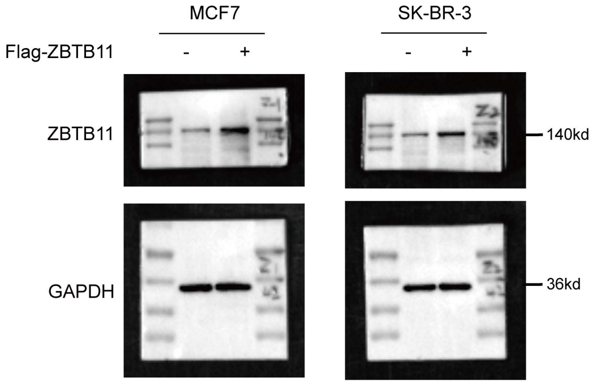

Supplementary Figure S2

C

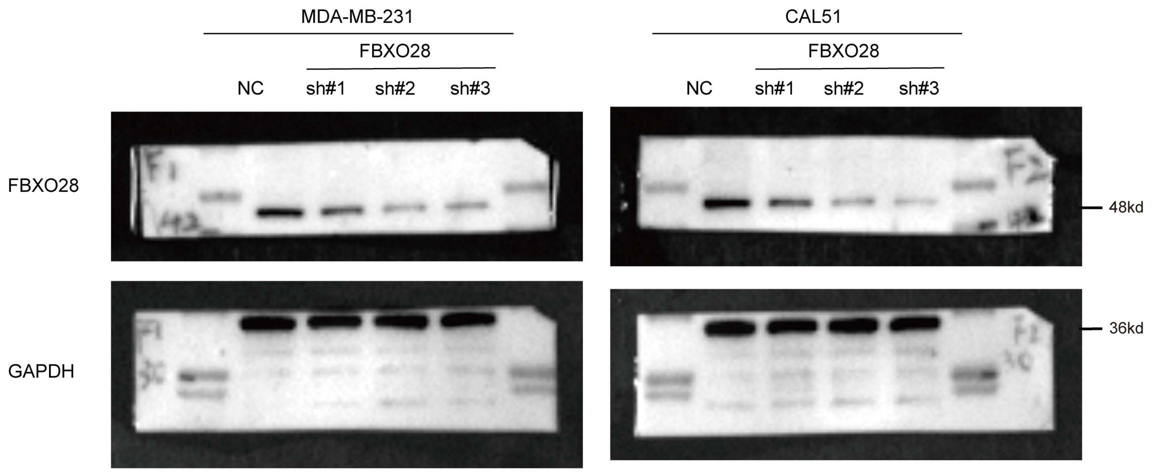

O

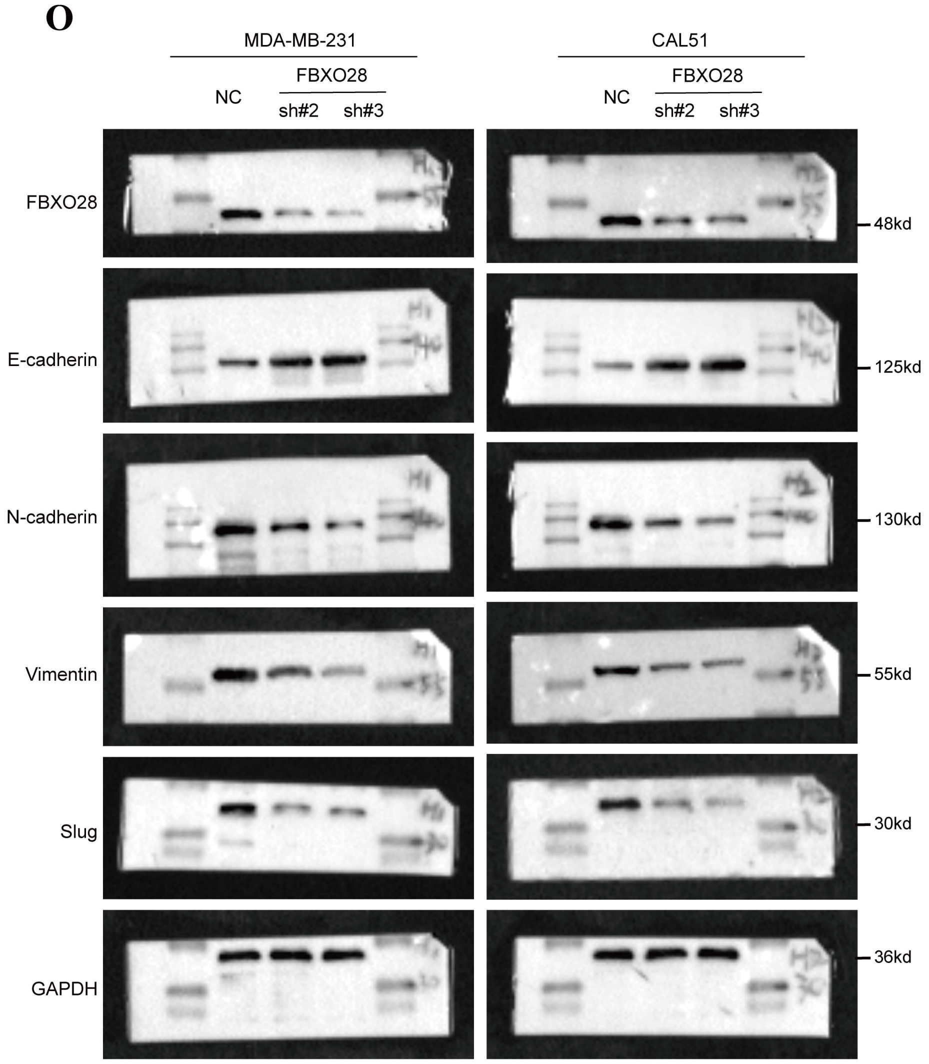

P

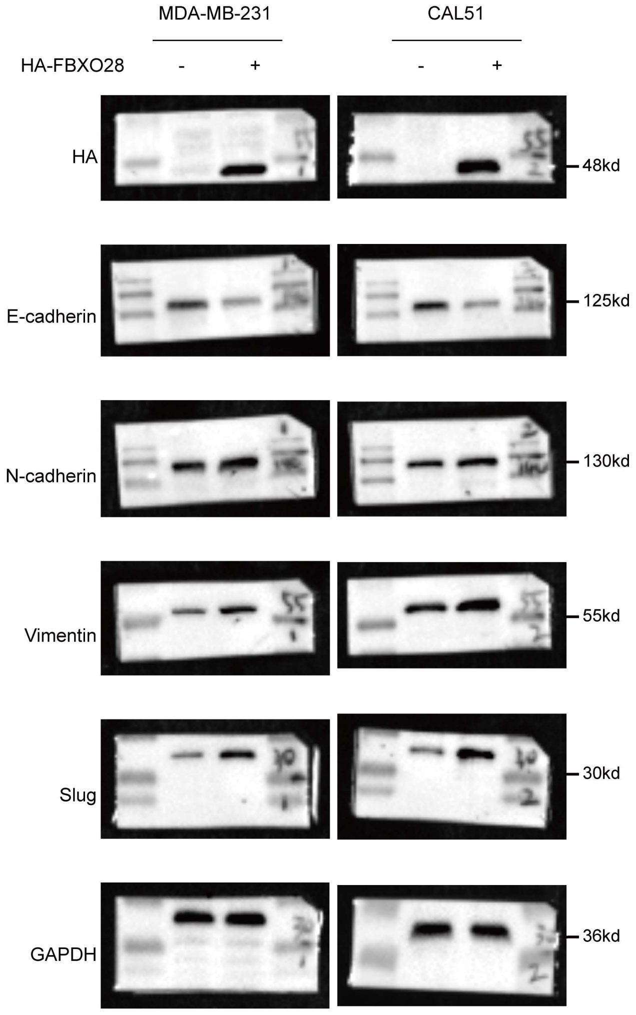

Supplementary Figure S3

A

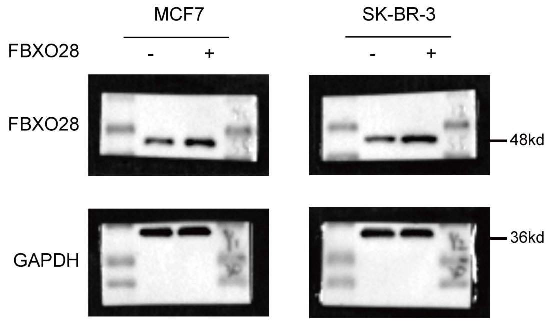

G

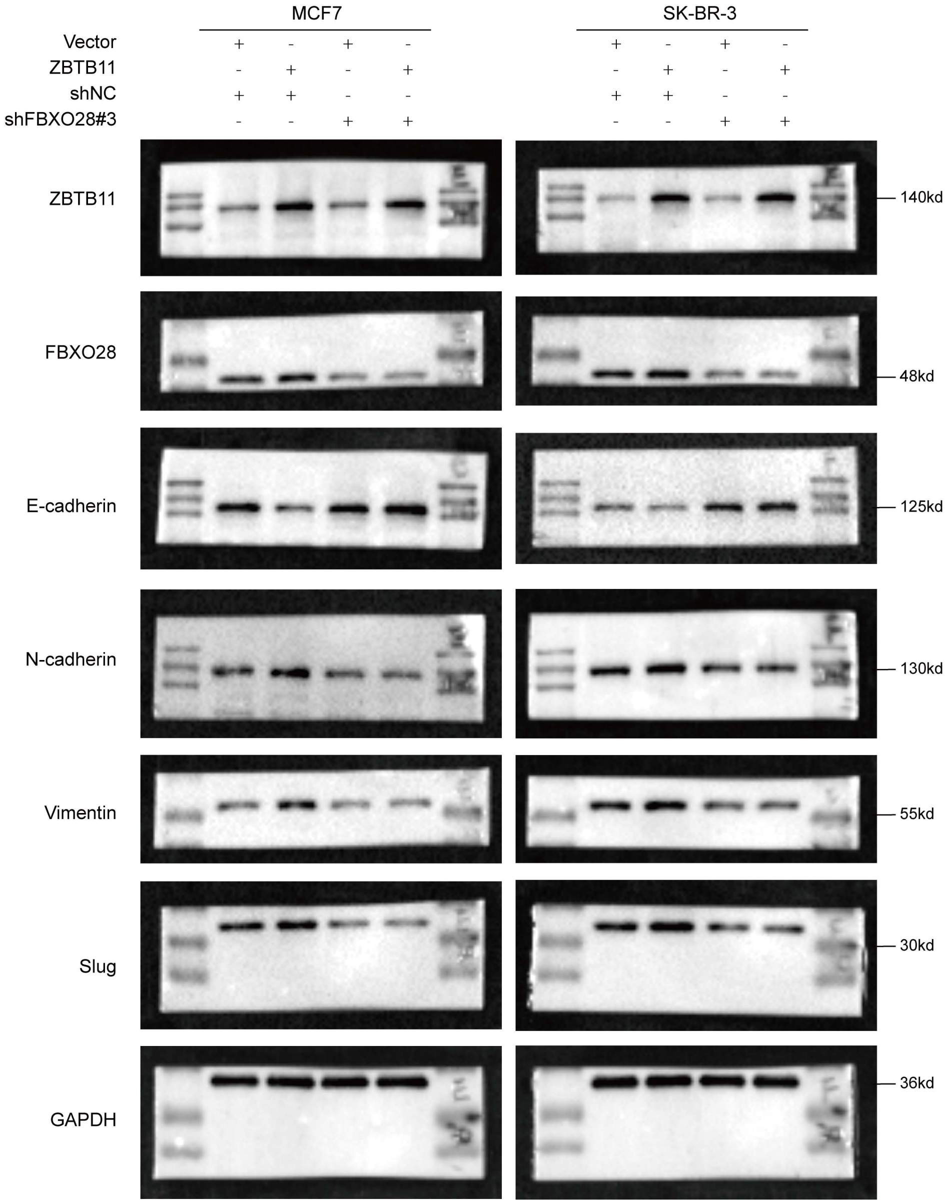

Supplementary Figure S4

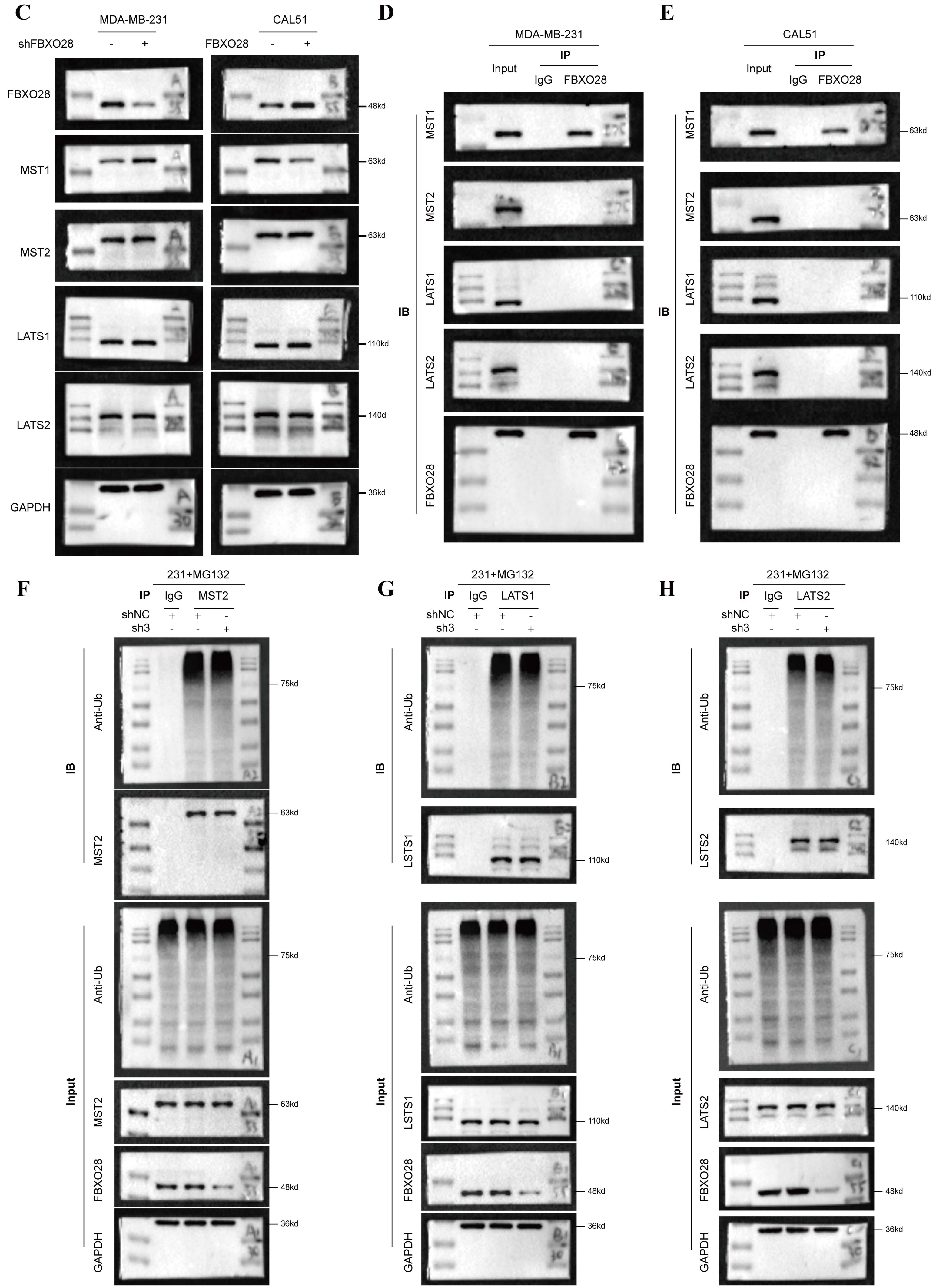

Supplementary Figure S4

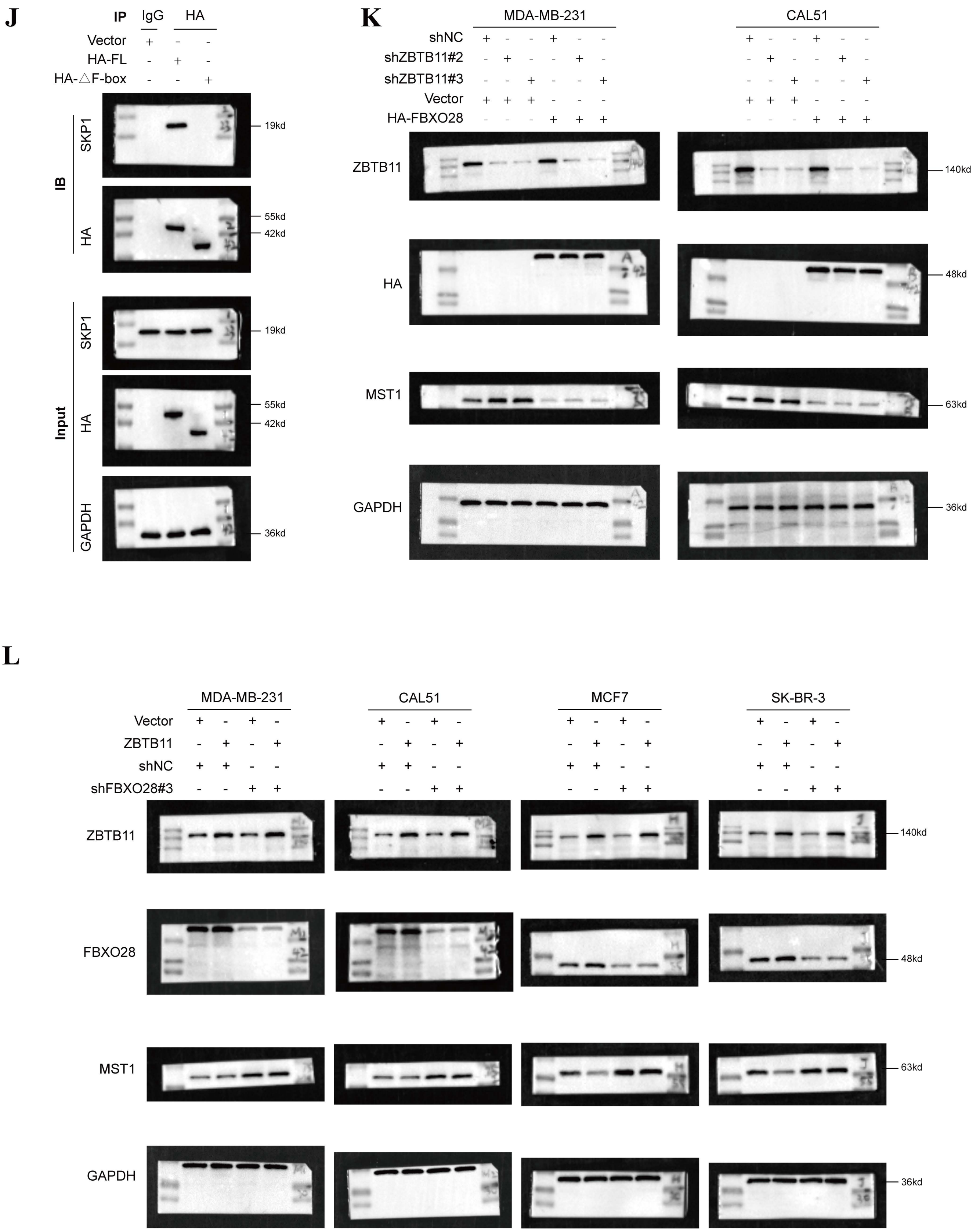

Supplementary Figure S5

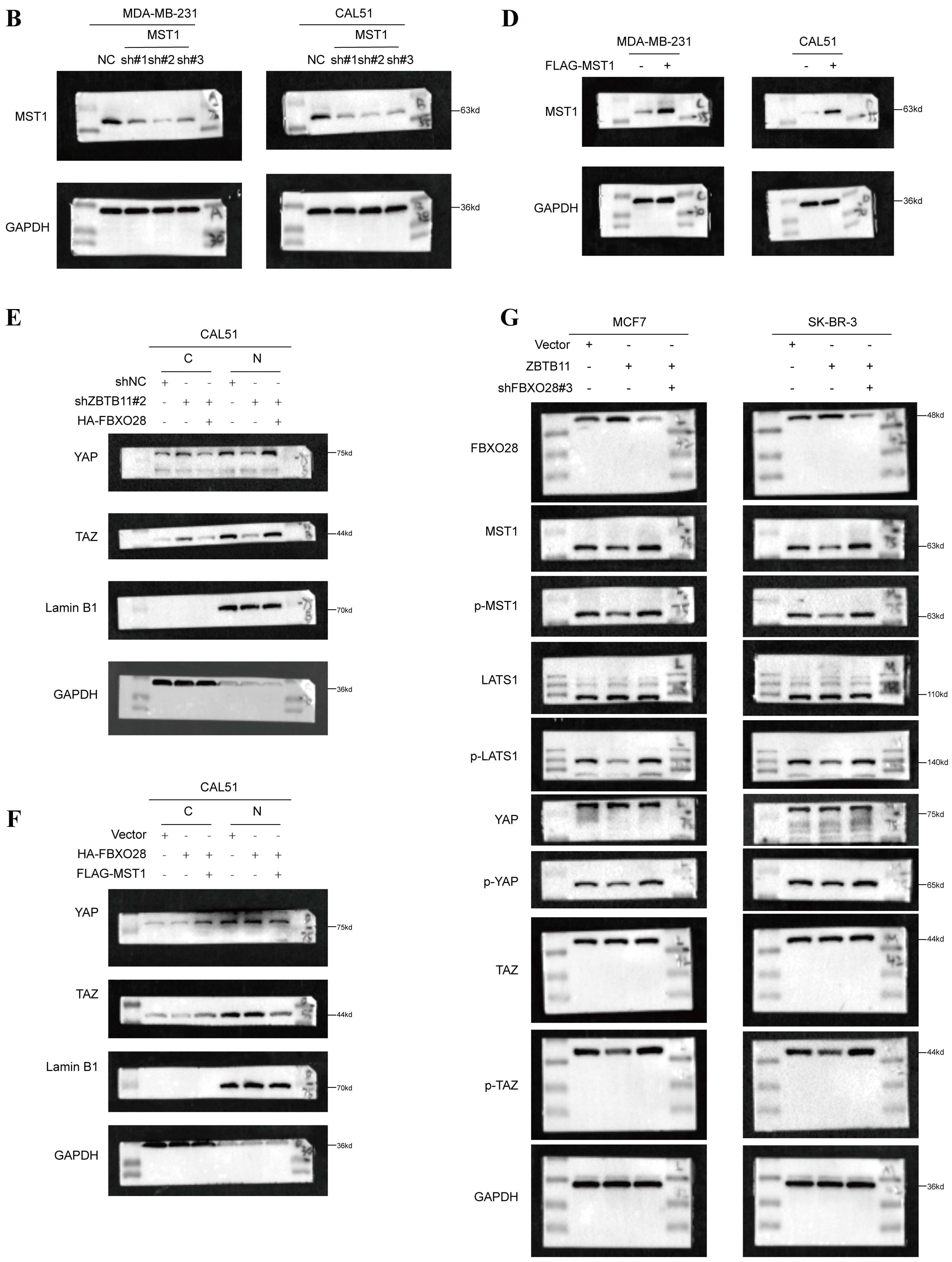

Supplement: Supplementary file 2 — Supporting File 2: advs76618‐sup‐0002‐data.zip. [file ADVS-9999-e76618-s001.zip › advs76618-sup-0002-data/Supplementary_Data_S6.pdf]
